# Metal-Ion Optical Fingerprinting Sensor Selection via an Analyte Classification and Feature Selection Algorithm

**DOI:** 10.1021/acs.analchem.4c06762

**Published:** 2025-03-27

**Authors:** Gabriel Petresky, Michael Faran, Verena Wulf, Gili Bisker

**Affiliations:** † Department of Biomedical Engineering, Faculty of Engineering, Tel Aviv University, Tel Aviv 6997801, Israel; ‡ Center for Physics and Chemistry of Living Systems, Tel Aviv University, Tel Aviv 6997801, Israel; § Center for Nanoscience and Nanotechnology, Tel Aviv University, Tel Aviv 6997801, Israel; ∥ Center for Light-Matter Interaction, Tel Aviv University, Tel Aviv 6997801, Israel

## Abstract

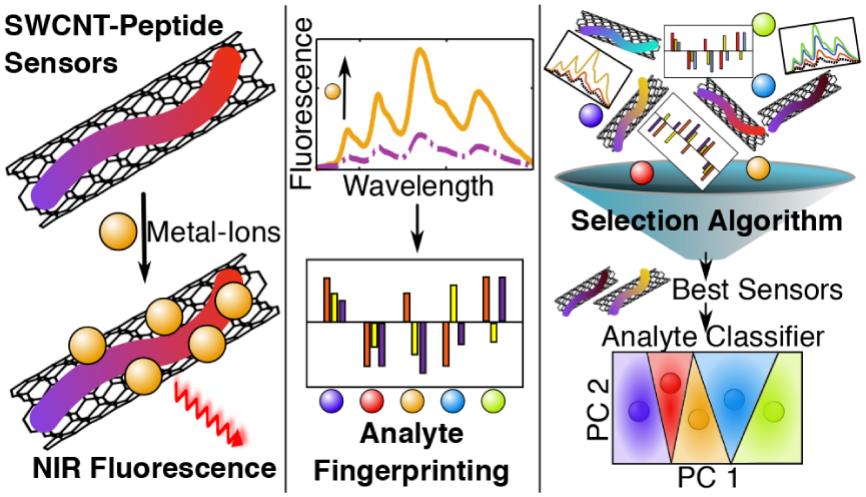

Accurate analyte classification remains a significant challenge in sensor technologies. We present the Analyte Classification and Feature Selection Algorithm (ACFSA), a computational tool designed to identify optimal sensor combinations from unique fingerprint patterns for analyte classification. We applied the ACFSA to a library of peptide-corona-functionalized single-walled carbon nanotubes (SWCNTs), developed as a near-infrared fluorescent, semiselective fingerprinting sensor set for detecting heavy metal ions. Inspired by natural metal-ion complexation sites, each SWCNT sensor in this library features a unique peptide sequence containing various amino acids for metal binding, revealing diverse optical response patterns to the various metal ions tested. The sensor library was further diversified using different SWCNT chiralities and photochemical modifications of the peptide coronae. The ACFSA was applied to the screening data of the fluorescence response of the 30 resulting SWCNT-peptide sensors to five metal-ion analytes. Through iterative dimensionality reduction and rational sensor selection, the algorithm identified the optimal fingerprinting sensors as a minimal two-sensor set with a 0.02% classification error. The final output of the ACFSA is thus an analyte classifier that serves as a unique analyte fingerprint pattern for the selected sensors. The developed peptide-SWCNT system serves as an effective proof-of-concept, illustrating the potential of our platform as a generally applicable tool for fingerprinting analytes and optimal sensor set selection in other sensor–analyte screening experiments.

Developing biomedical or biochemical sensors relies on achieving specific and selective analyte recognition.^1^ Typically, molecular recognition is facilitated by macromolecules forming three-dimensional binding pockets, providing unique binding interaction with the analyte,^2^ e.g., via hydrogen bonds, π–π stacking, or electronic interactions for metal complexation.^3−6^ Natural recognition units include, for example, aptamers,^7^ antibodies,^8^ or the active site of enzymes.^9^ Recent efforts in developing sensor recognition agents have demonstrated that synthetic (bio)-macromolecules and polymers, which form a molecular corona around the surface of nanoparticle sensors, adapt conformations that can enhance selectivity to certain analytes.^10−14^

Various polymers, as well as synthetic nucleic acid or peptide sequences, have been reported to form selective coronae for nanosensors,^15^ offering greater stability and reduced biodegradation compared to natural biomolecules.^8^ Further, using molecules without *a priori* inherent selectivity to the analytes allows for an extensive pool of potential sensor candidates for various analytes.^16−21^ Despite recent efforts for computational modeling of structure and functionality,^10,22,23^ as well as advancements in directed evolution,^24−26^ identifying a corona phase for selective sensing for a certain analyte still requires an extensive screening process. Although these sensors show high selectivity, matching the specificity of natural binding sites like aptamers or antibodies remains challenging.^27^

To enhance sensing accuracy, a fingerprinting approach using multiple sensors with distinct corona phases can be employed instead of relying on a single sensor.^18,20,28^ A fingerprint platform comprises multiple sensors that generate unique optical or electronic response patterns for each analyte. For an efficient and cost-effective platform, minimizing the sensor count while maintaining high identification accuracy is ideal. Achieving this requires screening a large pool of potential sensors, as a broader selection increases the likelihood of identifying an optimal minimal sensor set.

During this optimization process, the response of all potential sensors to all analytes must be recorded through a comprehensive screening process, resulting in a multidimensional data set, from which the optimal set of sensors must be selected via complex data analysis techniques. Thus, fingerprinting different analytes with multiple sensors can essentially be considered a multidimensional data classification task. One common approach to handling multidimensional data is principal component analysis (PCA),^29^ which transforms the data into a reduced dimensionality space.^20^ For multiple sensor applications, PCA ideally produces distinct response-data clusters for each analyte,^30^ which can then be used to generate an analyte classifier using methods like Bayesian optimal decision lines,^31^ or Voronoi tessellation diagrams.^32^ By comparing new measurement data against the resulting classifier, unknown analytes can be accurately identified.^33^

Many sensor platforms used in biomedicine rely on optical nanoparticles, enabling noninvasive and real-time analyte detection.^34^ A prime example of such optical nanosensors is single-walled carbon nanotubes (SWCNTs), which are carbon nanomaterials that can be thought of as graphene sheets rolled up into tubes.^35^ Their roll-up vector determines the diameter, as well as the chemical and electronic properties of the resulting SWCNT (*n,m*)-chirality.^35−37^ Semiconducting SWCNT chiralities allow for fluorescence emission in the near-infrared (NIR) wavelength range and fluorescence excitation in the visible to NIR range.^38^ Due to their fluorescence emission in the transparency window of biological tissue, they find widespread application in fluorescence sensing and imaging in biomedicine and bioengineering.^39−41^ The concept of functionalized SWCNTs as optical sensors has been developed for various applications, including the detection of enzymes,^42 −47^ RNA, lipids, and proteins;^11,48−50^ small molecules,^49,51−54^ pathogens,^16,20^ and reactive oxygen species,^55,56^ as well as multiple biomedical imaging and sensing applications.^38,57−61^

The SWCNTs’ optical properties also depend on the surface functionalization, which can be purely noncovalent or, include covalent modifications via the introduction of defect sites.^62^ The former maintains the sp^2^ lattice structure and is typically performed using macromolecules such as single-stranded DNA,^63^ surfactants,^64^ amphiphilic polymers,^45,65−67^ proteins,^68^ and suitable peptides^16,69^ that can bind the graphene lattice of the SWCNTs via hydrophobic interactions or π–π stacking.

When an analyte binds to the SWCNT corona phase, it can alter the polarity and dielectric environment, affecting the SWCNTs’ fluorescence emission wavelength and/or intensity.,^58,62,70^ Furthermore, studies have shown that individual (*n,m*)-chiralities can respond differently to a specific analyte, even when functionalized with the same corona phase.^71^ Thus, the fluorescence modulations of the SWCNTs have been found to be chirality-dependent,^72,73^ which enables SWCNT mixtures to function as multiple optical sensors, identifiable by their specific excitation and emission wavelengths – an advantage over other optical nanosensors.

The development of transition metal-ion sensors is crucial due to the significant effects of these metals on the environment and human health.^74^ Elevated levels of lead, copper, or chromium in living organisms can lead to enzymatic dysfunction and contribute to diseases like cancer and neurodegenerative disorders.^75^ Most metal-ion detectors rely on chelating molecules that form metal-specific binding sites,^5,76−78^ but their synthesis and stability pose challenges.^5^ Notably, previous studies have demonstrated the modulation of SWCNT fluorescence by divalent metal ions through DNA conformational changes on the nanotube surface.^79−81^ Building on these insights, the ability to enrich the sensing toolbox and identify an optimal, minimal set of sensors from a multidimensional data set while minimizing sensor redundancy could significantly enhance the scalability of fingerprinting platforms for metal-ion detection. Previously, we functionalized SWCNTs with fluorenylmethoxycarbonyl (Fmoc)-tyrosine, polymerized into a melanin-like material that presents quinone and catechol groups that are known to have metal chelation properties.^3,82^ While this system produced an optical response, it could not distinguish between different metals.^3^

In proteins and enzymes with metal-ion reaction centers, metal-binding sides are formed by the interplay of different amino acids, each with specific side-chain functional groups, such as amine groups in lysine or the carboxyl groups in glutamic acid.^83,84^ Similarly, SWCNTs functionalized with peptide coronas could enhance metal-ion recognition or classification. Modifying peptide sequences and corona chemistry, and using various SWCNT chiralities, would enable the construction of an extensive sensor library, from which an optimal fingerprinting set can be selected through data processing algorithms.

This work presents an optical fingerprinting sensor platform tested on transition metal-ions, where sensors are selected through an algorithmic iterative process, utilizing fluorescent SWCNTs functionalized with diverse peptide corona phases. Five Fmoc-peptides (Fmoc-FFFFYXYXY) were designed, each consisting of a phenylalanine chain (FFFF) and an alternating sequence of tyrosine (Y) and a variable amino acid (X). While the Fmoc-FFFF chain facilitates binding to the SWCNTs by π–π stacking,^85^ the alternating sequence YXYXY contains tyrosine to provide the same functionality exploited in our previous work,^3^ and functional groups such as guanidino, carboxyl, amine, and thiol, provided by four varying amino acids (arginine, glutamic acid, lysine, cysteine). Glycine, which lacks a side chain, was also included for comparison and for enriching the sensor library. Together, these functional groups, along with tyrosine and amide bonds, provide a range of potential sites for metal complexation (Scheme 1a). To further diversify the peptide library, the SWCNT-Fmoc-peptides underwent photochemical oxidization, yielding ten SWCNT-Fmoc-peptide sensors, including the oxidized and nonoxidized forms. By monitoring three chiralities in each SWCNT-peptide sample at their respective fluorescence excitation and emission wavelengths, we established a library of 30 potential sensors. The fluorescence intensity changes upon exposure to five metal ions – copper (Cu^2+^), nickel (Ni^2+^), chromium (Cr^3+^), lead (Pb^2+^), and mercury (Hg^2+^) – demonstrated that both the peptide corona phase and the SWCNT chirality influence the sensor response, resulting in a rich, multidimensional data set (Scheme 1b). To identify an optimal fingerprinting sensor set, we developed a selection and classification algorithm, namely Analyte Classification and Feature Selection Algorithm (ACFSA), which iteratively selects sensors via PCA-based dimensionality reduction, *k*-means clustering, and Chi-Squared feature selection (Scheme 1c). The iterative selection scheme runs until only one sensor remains or when a predefined accuracy threshold is met. This flexible methodology can be applied to other corona phase sensor–analyte screening experiments, facilitating the identification of the most effective fingerprinting sensor systems for analyte classification with higher detection certainty.

**Scheme 1:**
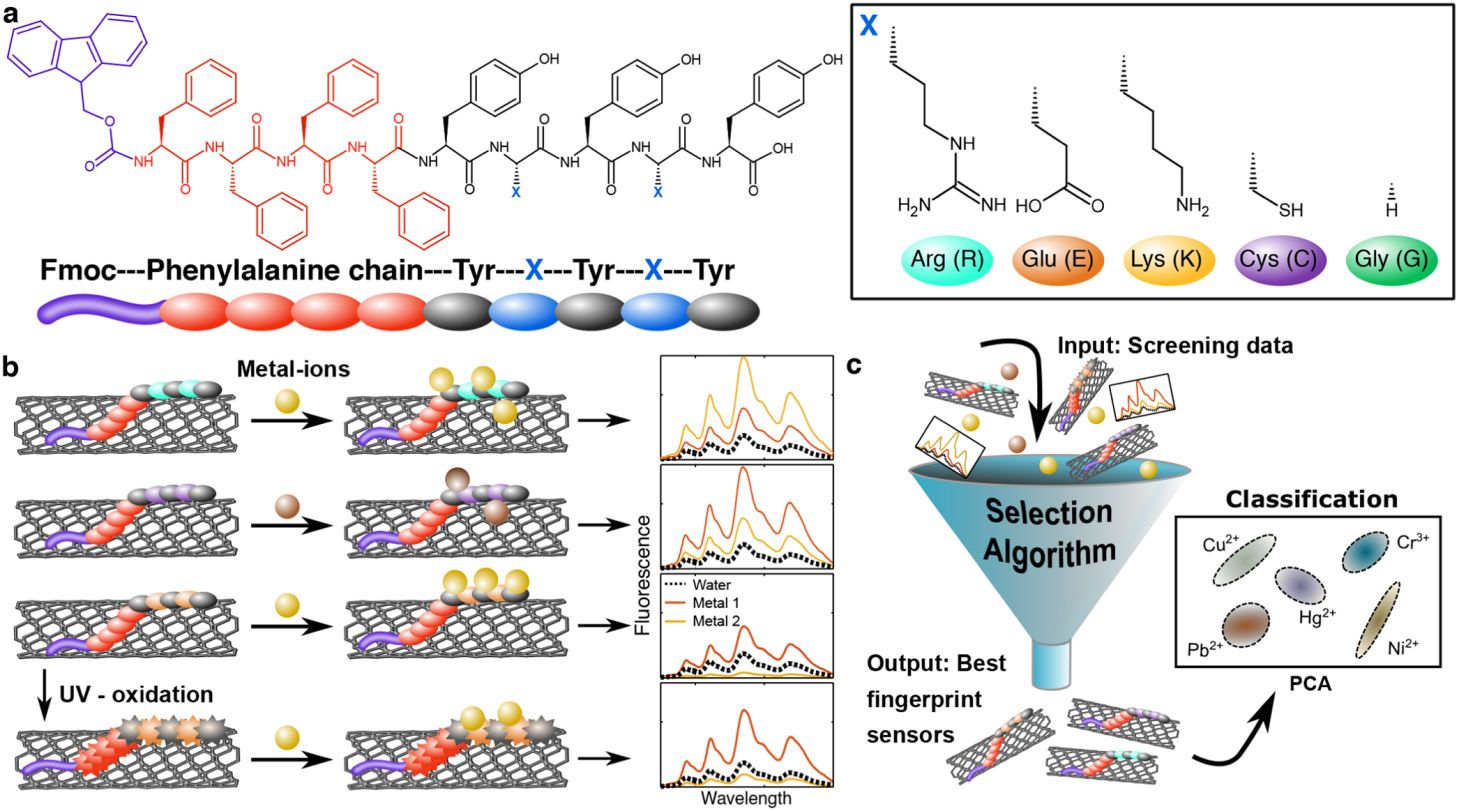
SWCNTs Suspended by Fmoc-Peptides as Fingerprinting Sensors (a) Corona phase peptide sequence and the varying amino acids side chains. (b) Fmoc-peptide and oxidized Fmoc-peptide functionalized SWCNTs interact with metal-ions, leading to different fluorescence responses. (c) The ACFSA algorithm finds an optimal sensor set for analyte classification

## Experimental Section

### Suspension of SWCNT with Fmoc-Peptides

Fmoc-peptides (15 mg, ABclonal) were dissolved in 1 mL of water, with solubility adjustments using NaOH or DMSO as needed (see the Supporting Information for additional details). HiPCO SWCNTs (2 mg, NanoIntegris) were dispersed in these solutions via tip sonication (4 W, 60 min, on ice). The suspensions were centrifuged twice (21,130 rcf, 1.5 h), filtered (Amicon, MWCO 100 kDa), and washed to remove excess peptides and equilibrate pH. The concentrations of the SWCNT-Fmoc-peptides suspension were determined using the extinction coefficient ε_632 nm_ = 0.036 L mg^–1^ cm^–1^,^86^ and found to be between 100 mg L^–1^ and 300 mg L^–1^.

### UV Oxidation

The SWCNT-peptide suspensions (10 mL, 50 mg L^–1^) were transferred into an 8 cm diameter glass Petri dish, and irradiated by λ = 254 nm UV light (Vilber, 6W) from above at a 4 cm distance for 90 min. Peptide oxidation was characterized via fluorescence (λ_ex_ = 280 nm and λ_em_ = 300–600 nm; and λ_ex_ = 320 nm and λ_em_ = 350–600 nm) using a plate reader (SPARK, Tecan).

### Transmission Electron Microscopy

SWCNTs-peptide suspensions (30 μL at 10 mg L^–1^) were dropped onto a carbon-coated grid and dried. The samples were stained by 30 μL of 2% (w/v) uranyl acetate solution, and then imaged using a JEM-1400plus TEM (JEOL, Japan) operating at 80 kV. Images were recorded using the SIS Megaview III camera and iTEM, the TEM imaging platform (Olympus).

### NIR Fluorescence Spectroscopy

Spectra were acquired using an inverted microscope (Olympus IX73) with a supercontinuum laser (NKT-photonics, 20 mW) with a bandwidth filter (Super-K varia) and spectrograph (HRS-300, Teledyne Princeton Instruments). Excitation–emission maps covered 500–840 nm (2 nm steps). For measuring the fluorescence response with the metals, the SWCNT-peptide suspensions (1 mg L^–1^) were incubated with metal-ions (Cu^2+^, Ni^2+^, Cr^3+^, Pb^2+^, Hg^2+^) at 300 μM for 15 min. NIR fluorescence was measured at excitation wavelengths 570, 660, and 730 nm, targeting SWCNT chiralities (6,5), (7,5), and (9,4) (see the Supporting Information for additional details).

### Sensor Response Analysis and Analyte Classification and Feature Selection Algorithm (ACFSA)

The peaks of the respective chiralities were fitted with a Lorentzian distribution function (MATLAB) and the relative intensity changes of the sensors to the different metal analytes were analyzed via the algorithm. A detailed description of the algorithm and a discussion of its performance is given in Section SIII.

## Results and Discussion

### Sensor Synthesis and Characterization

To explore the potential of fluorenylmethoxycarbonyl (Fmoc)-protected peptides as effective corona phases of SWCNTs for transition metal-ion sensing, we utilized the peptide sequence Fmoc-FFFFYXYXY, consisting of phenylalanine (Phe, F), tyrosine (Tyr, Y), and a variable amino acid (X), which is either arginine (Arg, R), glutamic acid (Glu, E), lysine (Lys, K), cysteine (Cys, C) or glycine (Gly, G) (Scheme 1a and Figure S1). These peptides were designed to provide functional groups through tyrosine and the variable amino acids, *i.e*., guanidine-, amine-, carboxyl-, hydroxyl-, and thiol-groups, to form a corona phase that can complex metal-ions.^3,83,87^ Glycine was included as an amino acid without an additional side chain. Further, the Fmoc-FFFF-tail facilitated the attachment of the Fmoc-peptide to the SWCNTs and their dispersion in water. Notably, without the additional phenylalanine-chain, the Fmoc-peptides showed very low efficiency as SWCNT dispersants (Figure S2).

**Figure 1:**
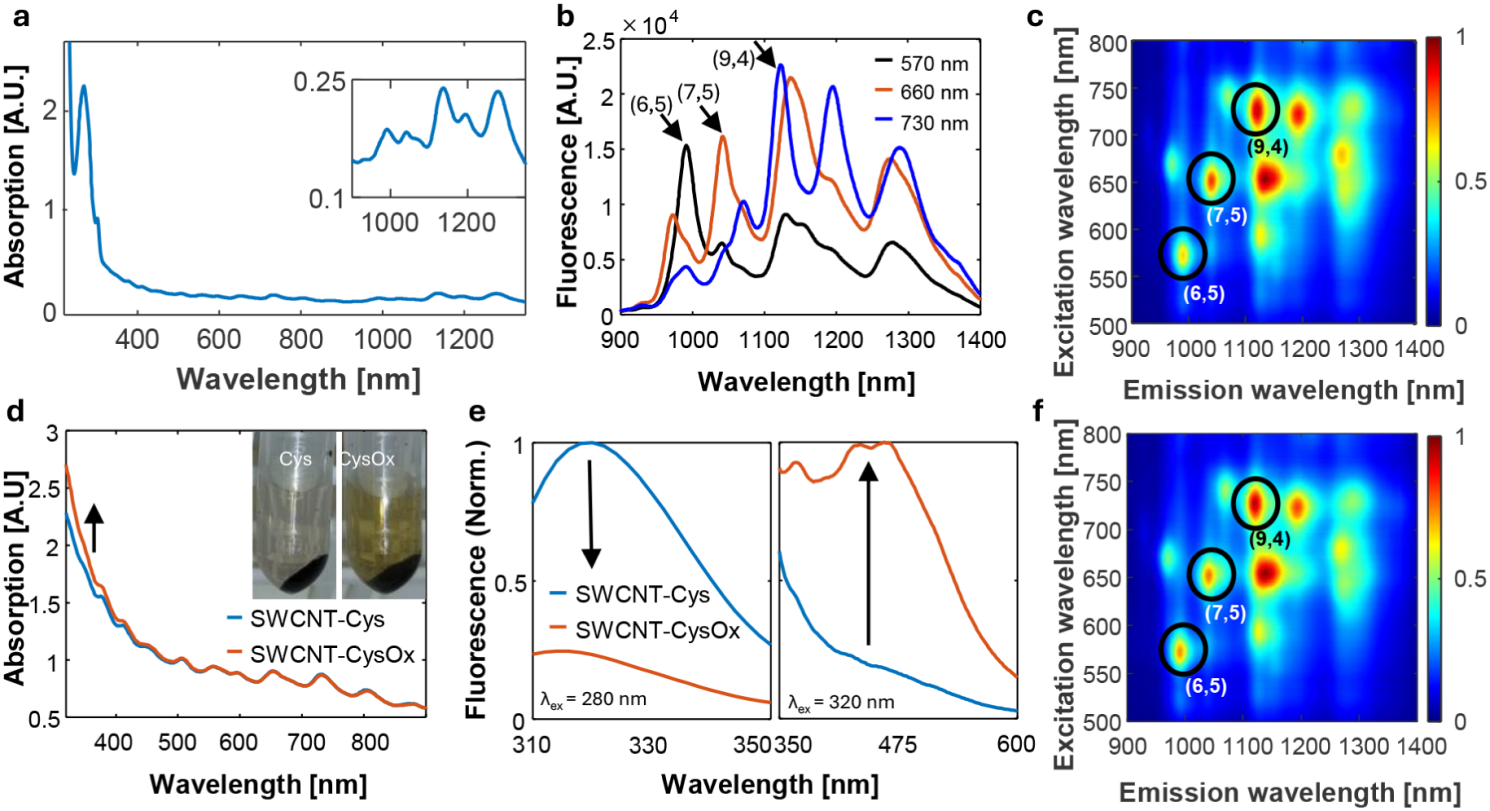
Functionalization, oxidation, and characterization of SWCNT-peptides. (a) UV–vis–NIR absorption spectrum of SWCNT-Cys, diluted 1:20 in water. Inset: NIR absorption of the SWCNTs-Cys. (b) Fluorescence emission spectra of SWCNT-Cys at three different excitation wavelengths: 570 nm (black), 660 nm (orange), and 730 nm (blue). Emission peaks of the three chiralities measured at these excitation wavelengths are marked with arrows corresponding to the (6,5), (7,5), and (9,4) chiralities, respectively. (c) Normalized excitation–emission map of SWCNT-Cys. The three chiralities shown in (b) are highlighted in circles. (d) Absorption spectra of SWCNT-Cys before (blue) and after UV-oxidation to SWCNT-CysOx (orange). Inset: Fmoc-peptides in solution before and after oxidation, following the precipitation of the SWCNTs with DMSO. (e) Normalized fluorescence emission of the peptides before (blue) and after (orange) UV-oxidation of SWCNT-Cys measured at excitation wavelengths of 280 nm (left) and 320 nm (right). (f) Normalized excitation–emission map of SWCNT-CysOx.

Figure 1a shows the absorption spectrum of SWCNTs successfully functionalized with Fmoc-FFFFYCYCY *via* direct tip sonication (SWCNT-Cys). The spectra of SWCNT-Cys along with the remaining Fmoc-peptide functionalized SWCNTs (SWCNT-Arg, SWCNT-Glu, SWCNT-Lys, and SWCNT-Gly) are shown in the Supporting Information (Figure S3). For all Fmoc-peptides, defined peaks corresponding to the SWCNTs’ E_11_ and E_22_ electronic transitions are clearly observed, indicating successful suspensions. Additionally, the peak at around 270 nm corresponds to the Fmoc-group, confirming the presence of the Fmoc-peptides in the corona phase of the SWCNTs after filtering the samples from excess Fmoc-peptides. However, the SWCNT surface coverage by the Fmoc-peptides varies depending on the peptide sequence, as evident from the different UV absorption of the Fmoc-group (Figure S3). SWCNT-Arg, for example, contains the least amount of Fmoc-peptide per milligram of SWCNTs, while SWCNT-Cys has the highest amount. These variations in the SWCNT surface coverage suggest different affinities of each peptide to the SWCNT surface and potential electrostatic repulsion between the Fmoc-peptides molecules. Nevertheless, all five SWCNT-peptide suspensions were stable in water for several weeks.

HiPCO SWCNTs combine several different chiralities within a single SWCNT sample, with each chirality characterized by its band gap energy, resulting in distinct fluorescence excitation and emission wavelengths. Further, it has been observed that each chirality can exhibit a distinct fluorescence response to analytes binding to their corona, influenced by the different curvature and surface coverage of the SWCNTs.,^36,71,72^ Consequently, every SWCNT-chirality in our SWCNT-peptide sample can be considered an independent sensor. For our measurements, we selected three different chiralities, namely the (6,5), (7,5), and (9,4) chiralities, which are excited at λ_ex_ = 570, 660, and 730 nm, respectively, and show fluorescence emission at around λ_em_ = 995, 1050, and 1130 nm, respectively (Figures 1b, c and S4). While various chiralities could be considered, we selected three that provide maximal spectral separation and minimal peak overlap, ensuring reliable and distinguishable fluorescence responses, making them well-suited for robust sensor analysis.

To further extend our SWCNT-peptide sensor library, we chose to photochemically oxidize the tyrosine-containing peptide corona of the SWCNTs *via* UV irradiation. Tyrosine and tyrosine-containing peptides that undergo photochemical oxidation are known to form dimers of dityrosine and, in some cases, to further oxidize to melanin-like derivatives.^88,89^ The resulting variations of the SWCNT corona are expected to affect analyte recognition^11^ through the modification of the corona phase morphology and the formation of metal-chelating catechol and quinone groups.^90^ In a previous study, Fmoc-tyrosine that underwent enzymatic oxidative polymerization *via* tyrosinase to a melanin-like material demonstrated improved stability as a SWCNT dispersant and enhanced performance in metal sensing attributed to the formation of metal-scavenging functional groups like catechols and quinones.^3^ Nevertheless, due to the more complex peptide structure in the current study, we chose photochemical oxidation instead of enzymatic oxidation.

For the oxidation of the Fmoc-peptide corona of the SWCNTs, we exposed the suspensions to UV light (254 nm) and monitored the reaction via absorption and fluorescence spectroscopy. Compared to SWCNT-Cys, the oxidized suspension after UV irradiation, onward referred to by the suffix Ox, SWCNT-CysOx, showed a slight increase in absorption below 500 nm, consistent with the observable browning of the Fmoc-peptide solution due to tyrosine oxidation also reported in previous works (Figure 1d).^3,90^ Additionally, the fluorescence emission of the peptide in the SWCNT-Cys suspension before and after UV-oxidation exhibited a decrease in the fluorescence intensity of tyrosine (λ_ex_ = 280 nm, λ_em_ = 320 nm) and an increase in the fluorescence intensity at λ_em_ = 420 nm, associated with the formation of dityrosine and its oxidized derivatives (Figure 1e).^89^ Similar results were obtained for the other suspensions, while for SWCNT-Arg, coloration was hardly observed, probably due to a lower amount of Fmoc-peptides in the corona phase of this particular sensor compared to the other sensors (Figure S5). The excitation–emission map of SWCNT-CysOx confirms that the fluorescence properties of the SWCNTs are maintained after UV-oxidation (Figures 1f and S4). Additional Raman spectroscopy analysis (Figure S6) comparing the spectra of each sensor before and after oxidation revealed no significant changes in the D band peak (around 1350 cm^–1^), confirming that the sp^2^ structure of the nanotubes remained intact. This suggests that the oxidation process was limited to the Fmoc-peptides, leaving the carbon nanotube chemical structure unaltered. As examples, TEM images of SWCNT-Lys and SWCNT-LysOx suspensions (Figure S7), and of SWCNT-Cys and SWCNT-CysOx suspensions (Figure S8) illustrate the SWCNT-peptide dispersion, as well as the changes in nanotube diameter and/or morphology following oxidation/polymerization.

Through the oxidation of the five Fmoc-peptides and the resulting chemically and structurally modified coronae, we obtain ten different SWCNT-peptide sensors. Further, by leveraging the different chiralities present in each SWCNT suspension, specifically focusing on the fluorescence response of three chiralities, (6,5), (7,5), and (9,4), we effectively increase the sensor library to a total of 30 distinct sensors.

### SWCNT-Peptide Metal-Ion Fingerprinting

**Figure 2:**
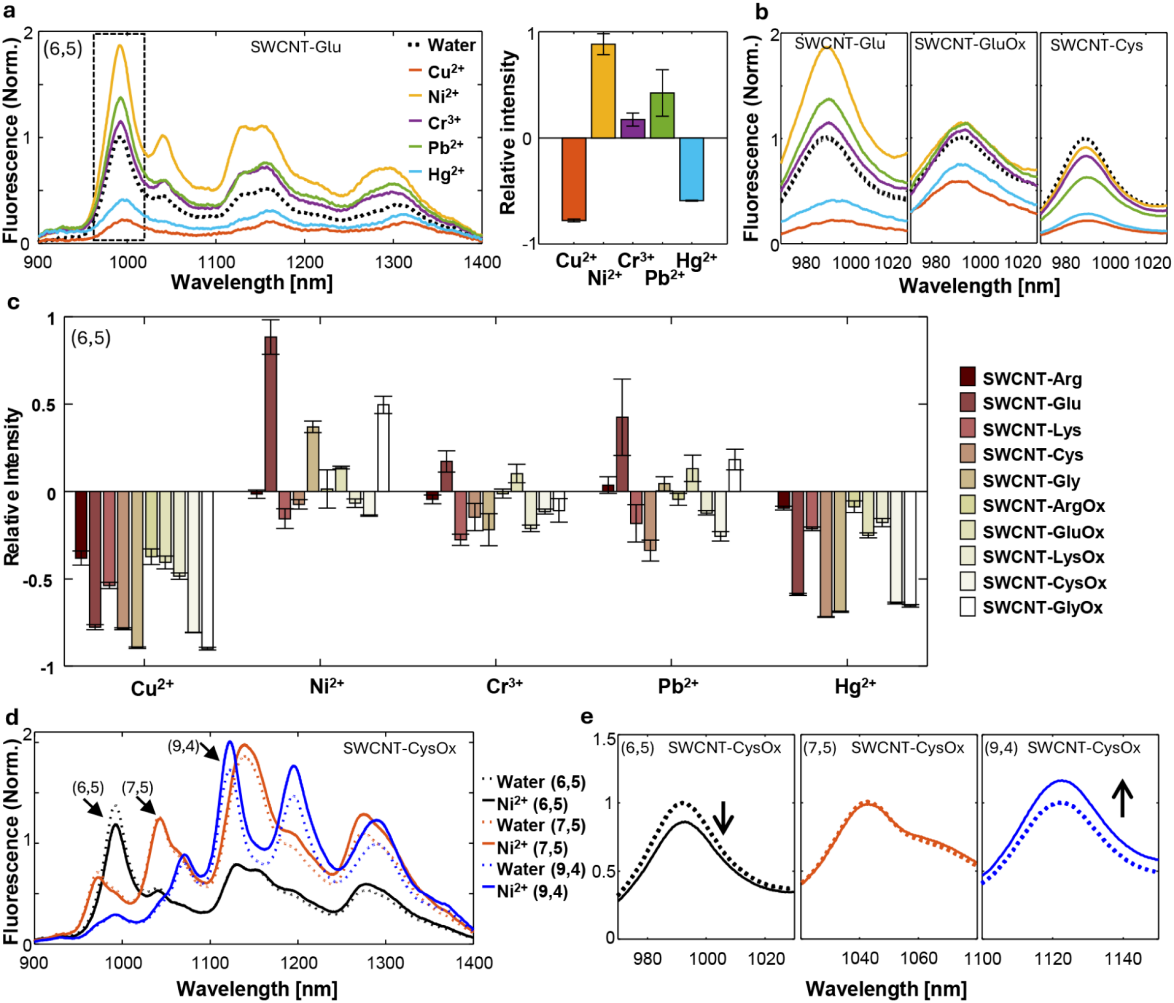
Fluorescence response of the SWCNT-peptide sensor in the presence of 300 μM of metal-ions. (a) Normalized fluorescence emission spectra of SWCNT-Glu, excited at 570 nm, before (dotted black line) and after the addition of metal-ions, Cu^2+^ (orange), Ni^2+^ (yellow), Cr^3+^ (purple), Pb^2+^ (green), and Hg^2+^ (blue). The dashed rectangle marks the peak of (6,5) chirality. The bar plot shows the relative fluorescence response for each metal-ion. *N* = 5. (b) Normalized fluorescence intensity of the (6,5) chirality of SWCNT-Glu, SWCNT-GluOx, and SWCNT-Cys before (dotted black line) and after the addition of metals-ions, Cu^2+^ (orange), Ni^2+^ (yellow), Cr^3+^ (purple), Pb^2+^ (green), and Hg^2+^ (blue). (c) Bar plot of the relative fluorescence response of the (6,5) chirality of all the SWCNT-peptide sensors in the presence of the metal-ions. Error bars represent the standard deviation of *N* = 5 measurements. (d) Normalized fluorescence emission spectra of SWCNT-CysOx in water (dotted lines) and after the addition of Ni^2+^ (continuous lines), measured at three excitation wavelengths: 570 nm (black), 660 nm (orange), and 730 nm (blue), corresponding to the excitation wavelengths of the (6,5), (7,5), and (9,4) chiralities, respectively. Arrows mark the peaks of the respective chiralities. (e) Normalized fluorescence intensity of the (6,5), (7,5), and (9,4) chirality of SWCNT-CysOx before (dotted lines) and after the addition of Ni^2+^ (continuous lines).

To develop a fluorescence fingerprinting sensor platform, each sensor’s response to each analyte must be characterized. By ensuring sufficient variability in fluorescence signals, a subset of sensors could be selected to generate a unique fingerprint for each analyte. To monitor the fluorescence response, each SWCNT-peptide sensor was exposed to the transition metal-ions Cu^2+^, Ni^2+^, Cr^3+^, Pb^2+^, and Hg^2+^ (300 μM)^3^ and measured in quintuplicates. The intensity changes were recorded at three different excitation wavelengths, 570, 660, and 730 nm, corresponding to the (6,5), (7,5), and (9,4) chiralities, respectively. Figure 2a shows the fluorescence emission spectra of one Fmoc-peptide sensor, SWCNT-Glu, excited at 570 nm, with the relative fluorescence response of the corresponding (6,5) chirality to the metal-ions. Notably, the sensor exhibited distinct fluorescence responses to each metal-ion: Ni^2+^, Cr^3+^, and Pb^2+^ induced varying degrees of intensity increase, with Ni^2+^ showing the highest increase, while Cu^2+^ and Hg^2+^ caused a decrease in intensity. The nature of the intensity change, whether an increase or decrease, remains an area of active research.^39^ Nevertheless, previous works reported that the fluorescence intensity decrease upon Cu^2+^ and Hg^2+^ might be attributed to quenching.^3,91^ Further, the modulation of the SWCNT fluorescence by metal ions might also be partly attributed to conformational changes in the corona phase of the nanotube, which could lead to changes in the accessible SWCNT surface area to solvent.^68,70,78,79,92^

Achieving a fingerprinting sensor system requires variations in the SWCNT-peptide sensor responses to the different analytes. Indeed, by comparing the fluorescence response of the (6,5) chirality of three sensors, SWCNT-Glu, SWCNT-GluOx, and SWCNT-Cys, we observed a corona-specific response toward the different metal analytes (Figure 2b). Additionally, the comparison between the responses of SWCNT-Glu and SWCNT-GluOx demonstrates that peptide oxidation leads to variations in fluorescence intensity response to metal addition, confirming the creation of independent sensors via chemical modifications of the peptide corona through the oxidation process. While the precise molecular structure of the peptides after oxidation may exhibit heterogeneous morphologies, making it challenging to fully characterize, oxidation is expected to lead to the formation of catechol and quinone groups.^90^ These functional groups have metal chelation properties, which may contribute to the fluorescence response mechanism of the SWCNT-peptide complexes.^90,93^ The relative fluorescence intensity changes of the (6,5) chirality of all ten SWCNT-peptide samples show different response patterns for each metal-ion, thereby generating an analyte-specific fingerprint (Figure 2c).

HiPCO SWCNT samples contain a mixture of different chiralities within a single suspension, where the differences in their optical properties enable us to treat each chirality as a single sensor probed by its distinct excitation and emission wavelengths.^71,72,94−97^ The fluorescence response of SWCNT-CysOx in the presence of Ni^2+^ measured at three different excitation wavelengths corresponding to the three chiralities (6,5), (7,5), and (9,4), exemplifies this point (Figure 2d), showing a turn-off response of the (6,5) chirality, no significant change of the (7,5) chirality, and a turn-on response for the (9,4) chirality (Figure 2e). These results demonstrate that each chirality of a SWCNT-peptide sample can have a distinct response to a particular analyte and, thus, can be regarded as an independent sensor. Figure S9 shows the bar plots for the relative fluorescence intensity changes of all the SWCNT-peptide sensors in response to the metal-ions for all three chiralities. To facilitate a clearer comparison between the fluorescence response of SWCNT-peptide sensors before and after oxidation, the data has been reordered in Figure S10, allowing for easier visual assessment of oxidation-induced changes. Additionally, in Figure S11, the data has been restructured to group the responses by sensor rather than by metal ion, emphasizing the variability in each sensor’s response across different metal ions.

In our sensing model, it is reasonable to expect that the variations in the fluorescence responses of the SWCNT-peptide sensors toward the analytes are due to the different binding affinities of metal-ions to the functional groups offered by the peptide-coronae or different wrapping conformations adopted by the corona phases. We observe that the sensor response is not clearly correlated with the Fmoc-peptide coverage per SWCNTs, suggesting that a higher peptide loading does not automatically produce a higher fluorescence response to metal-ions (Figure S12a). Furthermore, the zeta potential of the SWCNT-peptide sensors does not show a clear correlation with metal adsorption either (Figure S12b). Specifically, a negative zeta potential does not consistently lead to a higher fluorescence response by adsorbing more positively charged metal-ions. These findings suggest that the peptide structure variations play a key role in modulating fluorescence responses beyond simple electrostatic interactions or peptide surface coverage. Additionally, the observed variability in fluorescence responses across different SWCNT-peptide sensors reinforces the complexity of nonspecific sensing mechanisms and highlights the challenge of predicting sensor performance based solely on peptide sequence or theoretical considerations. This further emphasizes the need for experimental screening and data-driven approaches in sensor optimization for analyte classification.

### Sensor Set Optimization

**Figure 3:**
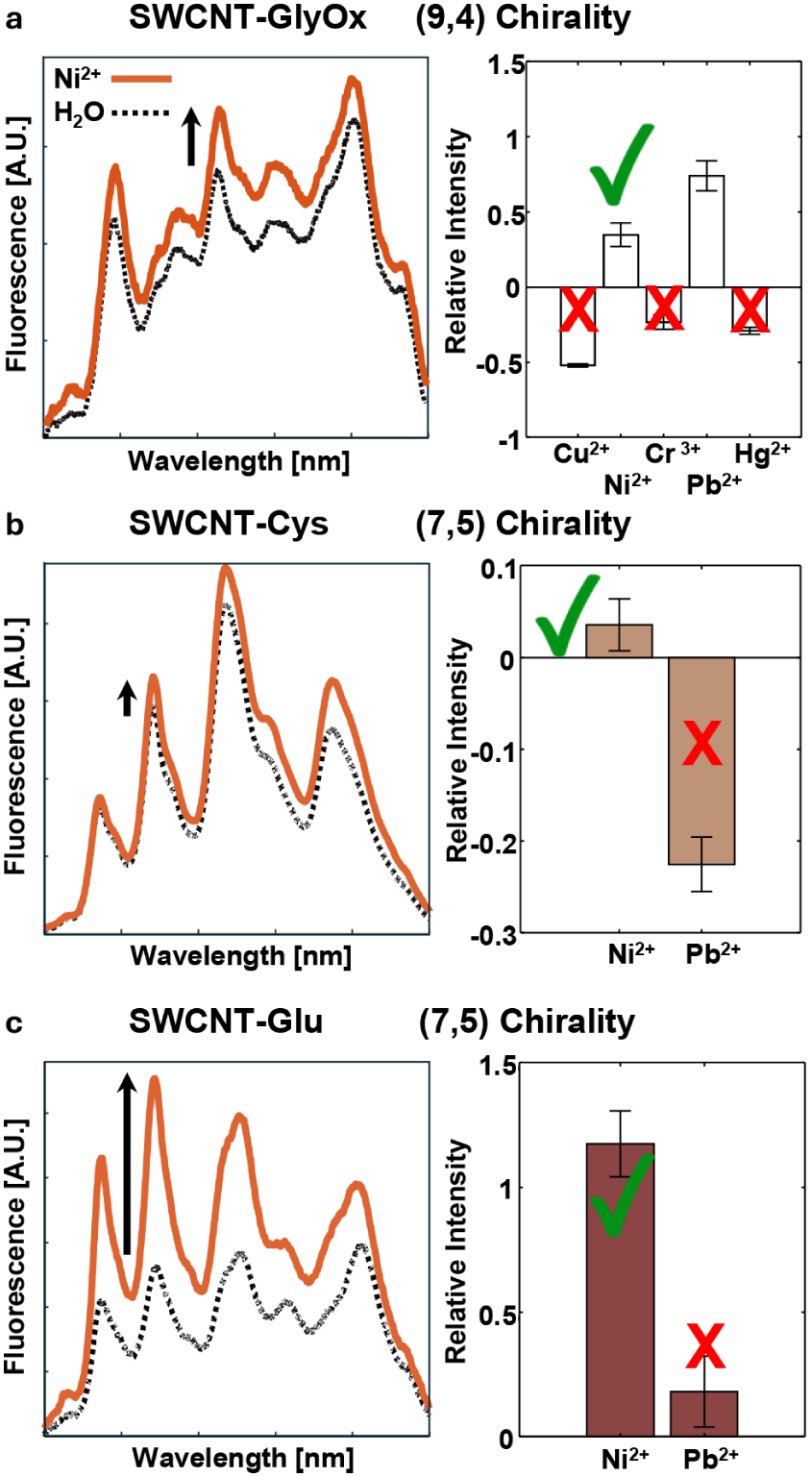
Example of an analyte identification procedure using several sensors. (a) The (9,4) chirality of SWCNT-GlyOx shows an intensity increase in response to the analyte, indicating Ni^2+^ or Pb^2+^. (b) A turn-on response of the (7,5) chirality of SWCNT-Cys excludes Pb^2+^. (c) Significant turn-on response of the (7,5) chirality of SWCNT-Glu further confirms Ni^2+^, in contrast to a minor turn-on response that would indicate Pb^2+^. All barplots *N* = 5.

To create a cost and time-efficient sensor platform, minimizing the number of sensors needed to identify a specific analyte is beneficial. For example, identifying Ni^2+^ does not require all 30 sensors, but rather, a subset of a small number of sensors can be sufficient to achieve a distinctive fingerprint. A turn-on response of the (9,4) chirality of SWCNT-GlyOx suggests the presence of either Ni^2+^ or Pb^2+^, as the other metal-ions would induce a decrease in the fluorescence intensity (Figure 3a). A subsequent experiment with the (7,5) chirality of SWCNT-Cys showing a small turn-on response in the fluorescence intensity would point toward Ni^2+^ since Pb^2+^ would have induced a fluorescence intensity decrease (Figure 3b). An additional experiment with the (7,5) chirality of SWCNT-Glu showing a significant turn-on response would confirm the presence of Ni^2+^ rather than Pb^2+^, as the latter would have caused only a slight increase in the emission intensity (Figure 3c). Therefore, to identify Ni^2+^ out of the five metal-ions, only two or three sensors would be sufficient. In our example, two sensors, SWCNT-GlyOx-(9,4) and SWCNT-Cys-(7,5), can identify the analyte, and a third sensor, SWCNT-Glu-(7,5), would simply increase the certainty of the sensing experiment.

**Figure 4:**
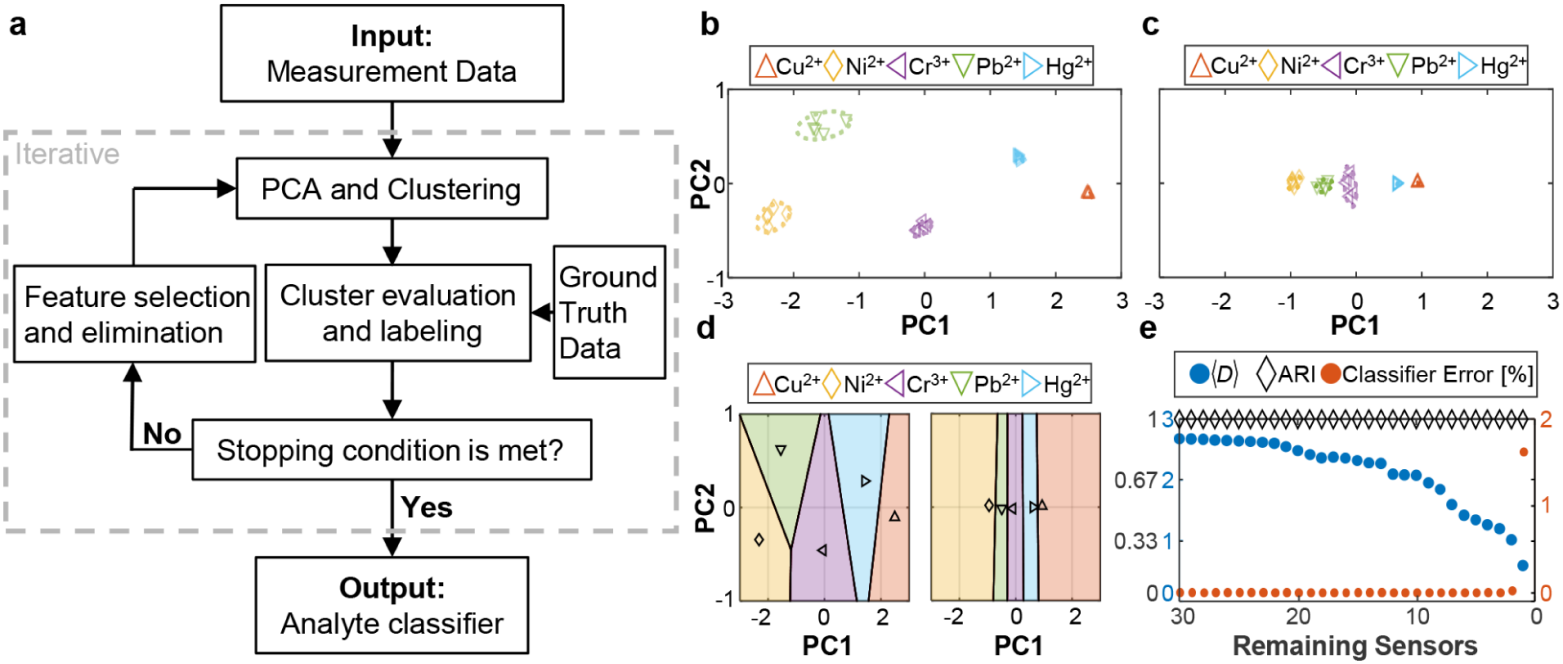
Analyte Classification and Feature Selection algorithm (ACFSA) for reducing the number of fingerprinting sensors and producing an analyte classification scheme. (a) Simplified flowchart of the algorithm. (b) 2D principal component representations of the data for all 30 sensors (colored shapes), including the 95% uncertainty ellipses of the clustering method (dashed lines). (c) 2D principal component representations of the data for the selected two sensors (colored shapes), including the 95% uncertainty ellipses of the clustering method (dashed lines). (d) The Voronoi classifier in the 2D principal components space for all 30 sensors (left) and for the selected two sensors (right) with the same color scheme as above. Points show the cluster center, and lines are defined by the intercluster distance. (e) The average intercluster distance, ⟨*D*⟩ (blue circles), the adjusted Rand index, ARI (black diamond), and the Voronoi classifier error (orange dots) for the remaining sensors.

Manually identifying an optimal sensor set for fingerprinting all five metal ions is complex and time-consuming. To streamline this process, we developed the Analyte Classification and Feature Selection Algorithm (ACFSA) that can perform the optimal sensor selection and results in an analyte classification pattern as a fingerprint that can be applied to identify analytes in similar measurement conditions (Figure 4a).

ACFSA is a feature selection algorithm where features correspond to sensors. The input data is the experimentally measured fluorescence response of the SWCNT-peptide sensors upon metal-ion addition. In our experiments, we measured the responses in 5 repetitions for the 30 SWCNT-peptide sensors and 5 different metal analytes (Figure S9). First, principal component analysis (PCA) reduces data dimensionality to two components, with PC1 and PC2 explaining 89% and 4.5% variability, respectively. Then, *k*-means clustering groups the data into five clusters, corresponding to the metal analytes, relying on the knowledge that the number of clusters *k* equals the number of analytes. The 95% confidence eclipses are computed under a Gaussian assumption for each cluster, with main axes also computed by the PCA algorithm (Figure 4b),^98^ and cluster labels were assigned using the Kuhn-Munkres algorithm.^99,100^ Further, the clustering performance of the algorithm was evaluated against the ground truth data using the adjusted Rand index (ARI), which measures the cluster similarity between the *k*-means and the ground truth data,^101^ where a perfect match would result in an ARI of 1, (100%), as observed in our results.

After clustering, the algorithm checks if a stopping condition is met, which can be a desired number of sensors or a desired clustering performance (ARI). If not, the algorithm proceeds to the feature (sensor) selection and elimination step, where one sensor per iteration is eliminated based on a Chi-squared feature ranking.^102^ The process repeats with the remaining sensors until the condition is satisfied. Figure 4c shows the PCA and clustering for the final two selected sensors. PC1 and PC2 of the selected sensors explain 99.5% and 0.5% of the data variability, respectively, and the data is separated into five clusters differentiated mainly by their PC1 values, while PC2 is nearly redundant. Despite using only two sensors, clustering achieves 100% ARI, confirming that this minimal set is sufficient for accurate classification.

Based on the selected sensors and their response pattern, an output analyte classifier was constructed utilizing the updated cluster centers to create a nearest neighbor Voronoi diagram.^33^Figure 4d shows the Voronoi analyte classifiers for the sensor sets, for all 30 sensors, and for the two remaining sensors after sensor selection, shown in Figure 4b and c. The classifier areas for each analyte in the diagram were determined by the intercluster distance *D* between the centers of the *k*-means clusters. To identify an unknown analyte, its sensor response should be transformed using PCA, retaining the top two components for comparison with the classifier. Notably, a higher sensor count increases intercluster distance in the Voronoi diagram, reducing the error of mismatching an analyte in the classifier.

To quantify the performance of the ACFSA and the expected classifier error, we monitored the ARI, the average intercluster distance ⟨*D*⟩, and the classifier error for the remaining sensor set after each iteration, starting from the entire 30 sensors set. The classification performance was quantified as a function of the number of surviving sensors, which experimentalists could use as a design principle given the desired accuracy (Figure 4e). A detailed description of the ACFSA is given in the Supporting Information, while further analysis of the results follows. Our data shows 100% ARI accuracy in each ACFSA iteration, as clustering aligns with ground truth and all data points fall within their respective cluster. This high accuracy stems from five-repetition measurements and a low ∼3% standard deviation per SWCNT-peptide sensor. To evaluate the robustness of our method in simulating real-world scenarios where unknown samples may be analyzed, we tested an artificial data set with 50 repetitions per measurement and a 4-fold increase in standard deviation compared to the original data for each sensor. This simulation, designed to mimic conditions with greater measurement error and variability, resulted in larger, overlapping clusters, with some ground truth data falling outside the 95% confidence ellipse of the clusters. Despite these challenges, the ARI accuracy remained high at around 90% across all sensors (with an expected decrease during the elimination process), demonstrating the potential of our classification approach even under more variable and uncertain conditions to classify unknown samples (Figure S13).

We also compared randomized feature selection with the Chi-squared method in the ACFSA (Figures S14 and S15). The randomized approach required six sensors for 100% ARI, while Chi-squared needed only two. This highlights the advantage of statistical selection, further validating the ACFSA’s effectiveness.

The sensor elimination reduces intercluster distance ⟨*D*⟩, increasing the likelihood of classification errors, which measure the overlap between each Gaussian distribution associated with a cluster and the neighboring Voronoi tiles. We quantified this by calculating ⟨*D*⟩ and the resulting classification error from the Voronoi classifiers’ data (Figure 4e), applying the ACFSA until only one sensor remained. The Pearson correlation (ρ = −0.5) confirmed a negative correlation between ⟨*D*⟩ and error. Thus, the intercluster distance is a useful metric for classification accuracy. With one sensor (SWCNT-Gly-(6,5)), the classification error was 1.6%, but with two sensors (SWCNT-Gly-(6,5) and SWCNT-GlyOx-(6,5)), it dropped to 0.02%, while ARI remained 100%.

For sensor selection, it is recommended to set a rational stopping condition. This condition, instead of a certain number of sensors, can be a minimum ARI, or a maximum classification error. To gain further understanding of the ACFSA on different data sets, we examined the algorithm for various subsets of our data, i.e., only oxidized for the three chiralities, only nonoxidized for the three chiralities, and single chirality SWCNT-peptide sensors, yielding a different surviving sensor set for each data subset when we apply a stopping condition of ARI = 100% and a classification error of <1% (Table S1 and Figures S16–S21). The lowest classification error (0.02%) was achieved using the full 30-sensor data set, while restricted initial sets resulted in higher errors and sometimes required more sensors, emphasizing the value of diverse screening data. This analysis highlights the trade-off between classification accuracy and experimental efficiency. While using all 30 sensors ensures the lowest error rate, it is not always practical due to increased complexity and resource demands. By optimizing for a minimal yet effective sensor set, we balance classification performance with experimental feasibility, demonstrating that a smaller, well-selected subset can achieve comparable accuracy with significantly reduced effort. Pearson correlation coefficients for the intercluster distance and classifier error are detailed in Table S2.

Analyzing the elimination process, we observed key trends in feature (sensor) selection. The Chi-squared single-feature elimination method ensures sensors are removed systematically rather than randomly. Instead, sensor measurements are uniformly discretized into ten values (predictor variables), which are compared against the cluster labels (response variable) using a contingency table. The Chi-squared test follows for each sensor, measuring the statistical dependency between its discretized values and the response variable, compared to the null hypothesis of independence. This sensor ranking reflects the relative importance of each predictor on the ACFSA resulting labels (Table S1, Figures 4e, and S16–S21). Nevertheless, the ARI can increase during elimination in some iterations (Figures S19d, S20d, and S21d), which may occur if one sensor data contains additional experimentally induced noise such that the two main PCA components do not necessarily lead to better data classification as the total data uncertainty increases. After this sensor is eliminated, the classification accuracy can improve.

As expected, a higher number of sensors in the fingerprinting set results in a higher intercluster distance of the classifiers and, therefore, in a smaller classification error. Clustering performance depends on the quality of the initial screening data, i.e., sufficient sensor response variability, sufficient repetitions, and low standard deviation of the measurement. A diverse sensor set improves these conditions, as shown by the different subsets of our sensor data. The ACFSA efficiently analyzes complete data sets within minutes, which makes it feasible for larger sets. A complexity analysis estimates that the ACFSA, with *O(n*^4^) complexity and assuming a single run for 30 features takes two minutes, can process approximately 300 sensors in 2 weeks (see the Time complexity analysis section in the SI). Given the experimental challenge of handling 300 sensors, the ACFSA remains a valuable tool for sensor selection.

We applied the ACFSA to a binary response set, considering only fluorescence trends, which are either turn-on or turn-off responses. In this framework, an increase in intensity was assigned a value of +1, whereas a decrease was assigned −1. We further assume that this response trend remains consistent across concentrations within the sensors’ dynamic range, thereby removing direct concentration dependence. This allows for classification based on relative fluorescence change direction rather than intensity magnitudes. The constructed PCA, in this case, thus removes concentration dependency (Figure S22a). The binary classifier successfully classified chromium, partly classified between nickel and lead due to some overlap, but was unable to distinguish copper from mercury due to their identical negative response profiles (Figure S22b). This approach demonstrates the classification potential, with expanded measurements across more concentrations potentially enabling full analyte classification (see more details in the Supporting Information).

### Sensor Robustness and Practical Considerations

To further explore concentration-dependent responses, we analyzed SWCNT-Gly-(6,5), the most effective classification sensor (Figure S23). We found detection limits of 3.0 × 10^–9^ M for copper, 7.2 × 10^–5^ M for chromium, 7.1 × 10^–7^ M for mercury, 8.1 × 10^–6^ M for lead, and 3.6 × 10^–5^ M for nickel. While detection limits varied, the fingerprinting patterns remained stable across concentrations, supporting the sensors’ utility for classification tasks across varying concentrations and broader data set applications. Importantly, to achieve quantification or classification of various concentration values, a complete data set covering the concentration range of interest would need to be systematically acquired and analyzed using the ACFSA algorithm. To test functionality in different environments, we analyzed SWCNT-Gly-(6,5) in serum and mineral water (Figure S24). Both showed distinct fingerprinting patterns, likely due to interactions with serum components or dissolved minerals. These results suggest that peptide-SWCNT sensors can adapt across matrices with matrix-specific adjustments. Extending the application of these sensors to additional environments with different interfering substances would require a dedicated set of fingerprinting experiments to generate environment-specific data sets. Ultimately, the same algorithm used for sensor selection in this study would be applied to the newly tested data sets, enabling an optimized identification of minimal sensor sets tailored to the respective conditions.

To assess batch-to-batch variability, we tested the SWCNT-Arg and SWCNT-Glu sensors across three peptide batches, including de novo sensor synthesis and fluorescence response measurements (Figure S25). While fluorescence intensity variations of up to 20% were observed, the overall fingerprinting pattern remained stable. This suggests that while batch-specific data sets may be required, the overall classification performance of the system is maintained across batches.

To assess the stability of the sensors over time, we conducted both short-term and long-term stability experiments. The fluorescence signal of all sensors was monitored under continuous laser irradiation for 9 h (Figure S26), demonstrating stable responses with only minor drifts observed in SWCNT-ArgOx. Additionally, we retested the same SWCNT-Gly sensor batch after 6 months, stored at 4 °C (Figure S27), confirming that while relative intensity variations of 10–20% were present, the overall fingerprinting pattern remained intact. Furthermore, the suspension showed no aggregation after these 6 months (Figure S28), indicating long-term colloidal stability. These results suggest that the sensors can be reliably used over extended periods without loss of functionality.

These analyses highlight the adaptability of the peptide-SWCNT sensor system for broader analyte classification and sensor selection across diverse conditions.

## Conclusion

We developed a comprehensive approach to synthesize Fmoc-peptide functionalized SWCNTs with varying amino acids in the peptide sequence, as the corona phase, to generate a near-infrared fluorescent sensor library for transition metal-ion fingerprinting and classification via a dedicated algorithmic framework. The Fmoc-peptides stabilized SWCNT suspensions in water while maintaining their NIR fluorescence. UV exposure induced photochemical polymerization and oxidation of the tyrosine side chains, leading to structural and chemical changes in the peptide corona, thus extending the SWCNT-peptide sensor library to 30 sensors. The fluorescence response patterns of three different chiralities, (6,5), (7,5), and (9,4), of the SWCNT-peptide sensors to transition metal-ions, Cu^2+^, Ni^2+^, Cr^3+^, Pb^2+^, and Hg^2+^, were used for constructing a fingerprint library for the five analytes.

To efficiently navigate this extensive library and identify the most effective set suitable as a fingerprinting sensor platform for metal-ion classification, we developed the Analyte Classification and Feature Selection Algorithm. The algorithm performs iterative sensor selection and elimination until a stopping condition is met, yielding an analyte classifier as output with a corresponding Voronoi diagram for analyte classification. The algorithm also provides information on the selection process, including the intercluster distance ⟨*D*⟩, ARI data, and Voronoi classifier error, which can be used to analyze the quality of any sensor set. Future work may improve the ACFSA scheme by considering other feature selection algorithms, such as genetic algorithms,^103,104^ or by including more than two leading components for the PCA step before clustering the data.

Starting from the full 30 sensors library and setting stopping conditions of a maximum 1% classification error and ARI of 100%, in a few minutes of runtime, the ACFSA selected the sensors SWCNT-Gly-(6,5) and SWCNT-GlyOx-(6,5), which achieved the classification of the five analytes, with a classification error of 0.02%. Manual sensor selection, in contrast, would require iteratively analyzing fluorescence patterns, discarding redundant sensors, and reevaluating classification accuracy – a significantly more time-consuming and impractical process that becomes exponentially more complex as the sensor library grows, highlighting the efficiency of the ACFSA framework in automating sensor selection with speed, accuracy, and scalability. Testing on artificially generated data sets with larger variance confirmed robustness, maintaining 90% ARI even under increased uncertainty.

Our results demonstrated concentration-independent classification for chromium, and partial classification for nickel and lead. Additionally, SWCNT-Gly, the last sensor to remain in the elimination process, maintained the fingerprint patterns across concentrations, meaning that the fluorescence response trend – whether an increase (turn-on) or a decrease (turn-off) in intensity – remained the same for each metal-ion at all tested concentrations. The limits of detection varied from the nanomolar range for copper to the micromolar range for nickel. These results underscore the potential for extending this approach to more comprehensive data sets that include a wide range of analyte concentrations.

The methodology and algorithm developed here can be generalized and applied to other corona phase sensor–analyte screening experiments. This capability offers a powerful tool for analyzing multidimensional data obtained from these experiments, optimizing sensor selection, and enhancing analyte classification’s overall accuracy and efficiency across various applications. The flexibility and adaptability of this approach pave the way for future advancements in fingerprinting sensor technology and its application to classifying diverse molecular analytes.

## Supporting Information Available

Supporting experimental section; molecular structure of the Fmoc-peptides; stability photograph; absorption and fluorescence spectra of the SWCNT-peptide suspensions; absorption and fluorescence spectra of SWCNT-peptide oxidation; Raman spectra of the SWCNTs-peptides before and after oxidation; TEM images; relative fluorescence intensity changes of the sensors with each metal at three different chiralities per metal and per peptide; correlation between the fluorescence response vs peptide load or Zeta potential; detailed explanation and performance analysis for all sensor subsets of the analyte classification and feature selection algorithm; ACFSA time complexity analysis; ACFSA implementation with simulated data. ACFSA with randomized feature selection; Pearson correlation coefficients for the intercluster distance with the classifier error ρ(⟨*D*⟩,error), and for the ARI with the classifier error table; ACFSA implementation with experimental data; PCA results for binary responses of the 30 sensors and five metal-ions; calibration curves and limit of detection for SWCNT-Gly sensor and the five metal-ions; relative fluorescence response of SWCNT-Gly to the metal-ions in serum and mineral water; sensor stability (PDF)

## References

[ref1] Morales M. A.; Halpern J. M. Guide to Selecting a Biorecognition Element for Biosensors. Bioconjugate Chem. 2018, 29 (10), 3231–3239. 10.1021/acs.bioconjchem.8b00592. PMC6416154 30216055

[ref2] Kim D. C.; Kang D. J. Molecular Recognition and Specific Interactions for Biosensing Applications. Sensors 2008, 8 (10), 6605–6641. 10.3390/s8106605. 27873889 PMC3707470

[ref3] Wulf V.; Bichachi E.; Hendler-Neumark A.; Massarano T.; Leshem A. B.; Lampel A.; Bisker G.; Hendler-Neumark A. Multicomponent System of Single-Walled Carbon Nanotubes Functionalized with a Melanin-Inspired Material for Optical Detection and Scavenging of Metals. Adv. Funct. Mater. 2022, 32 (49), 2209688 10.1002/adfm.202209688.

[ref4] Blake D. A.; Jones R. M.; Blake R. C.; Pavlov A. R.; Darwish I. A.; Yu H. Antibody-Based Sensors for Heavy Metal Ions. Biosens. Bioelectron. 2001, 16 (9–12), 799–809. 10.1016/S0956-5663(01)00223-8. 11679258

[ref5] Carter K. P.; Young A. M.; Palmer A. E. Fluorescent Sensors for Measuring Metal Ions in Living Systems. Chem. Rev. 2014, 114 (8), 4564–4601. 10.1021/cr400546e. 24588137 PMC4096685

[ref6] El-Safty S. A.; Prabhakaran D.; Ismail A. A.; Matsunaga H.; Mizukami F. Nanosensor Design Packages: A Smart and Compact Development for Metal Ions Sensing Responses. Adv. Funct. Mater. 2007, 17 (18), 3731–3745. 10.1002/adfm.200700447.

[ref7] Cho E. J.; Lee J.-W.; Ellington A. D. Applications of Aptamers as Sensors. Annu. Rev. Anal Chem. 2009, 2 (1), 241–264. 10.1146/annurev.anchem.1.031207.112851. 20636061

[ref8] Byrne B.; Stack E.; Gilmartin N.; O’Kennedy R. Antibody-Based Sensors: Principles, Problems and Potential for Detection of Pathogens and Associated Toxins. Sensors 2009, 9 (6), 4407–4445. 10.3390/s90604407. 22408533 PMC3291918

[ref9] Nguyen H. H.; Lee S. H.; Lee U. J.; Fermin C. D.; Kim M. Immobilized Enzymes in Biosensor Applications. Materials 2019, 12 (1), 121 10.3390/ma12010121. 30609693 PMC6337536

[ref10] Zhang J.; Landry M. P.; Barone P. W.; Kim J.-H.; Lin S.; Ulissi Z. W.; Lin D.; Mu B.; Boghossian A. A.; Hilmer A. J.; Rwei A.; Hinckley A. C.; Kruss S.; Shandell M. A.; Nair N.; Blake S.; Şen F.; Şen S.; Croy R. G.; Li D.; Yum K.; Ahn J.-H.; Jin H.; Heller D. A.; Essigmann J. M.; Blankschtein D.; Strano M. S. Molecular Recognition Using Corona Phase Complexes Made of Synthetic Polymers Adsorbed on Carbon Nanotubes. Nat. Nanotechnol. 2013, 8 (12), 959–968. 10.1038/nnano.2013.236. 24270641 PMC5051352

[ref11] Bisker G.; Dong J.; Park H. D.; Iverson N. M.; Ahn J.; Nelson J. T.; Landry M. P.; Kruss S.; Strano M. S. Protein-Targeted Corona Phase Molecular Recognition. Nat. Commun. 2016, 7 (1), 10241 10.1038/ncomms10241. 26742890 PMC4729864

[ref12] Denizli A. Molecular Imprinting for Nanosensors and Other Sensing Applications Elsevier 2021 1–417

[ref13] Adampourezare M.; Nikzad B.; Nasrollahzadeh S.; Asadpour-Zeynali K.; de la Guardia M.; Ezzati Nazhad Dolatabadi J.; Zhang F.; Mahdi Jafari S. Polysaccharide-Based Sensors and Nanosensors: A Review of Recent Progress and Challenges. Microchem. J. 2024, 204, 110944 10.1016/j.microc.2024.110944.

[ref14] Nocerino V.; Miranda B.; Tramontano C.; Chianese G.; Dardano P.; Rea I.; De Stefano L. Plasmonic Nanosensors: Design, Fabrication, and Applications in Biomedicine. Chemosensors 2022, 10 (5), 150 10.3390/chemosensors10050150.

[ref15] Zhang L.; Yang Y.; Tan J.; Yuan Q. Chemically Modified Nucleic Acid Biopolymers Used in Biosensing. Mater. Chem. Front. 2020, 4 (5), 1315–1327. 10.1039/D0QM00026D.

[ref16] Shumeiko V.; Zaken Y.; Hidas G.; Paltiel Y.; Bisker G.; Shoseyov O. Peptide-Encapsulated Single-Wall Carbon Nanotube-Based Near-Infrared Optical Nose for Bacteria Detection and Classification. IEEE Sens. J. 2022, 22 (7), 6277–6287. 10.1109/JSEN.2022.3152622.

[ref17] Zong C.; Fang L.; Song F.; Wang A.; Wan Y. Fluorescent Fingerprint Bacteria by Multi-Channel Magnetic Fluorescent Nanosensor. Sens. Actuators, B 2019, 289, 234–241. 10.1016/j.snb.2019.03.091.

[ref18] Amir D.; Hendler-Neumark A.; Wulf V.; Ehrlich R.; Bisker G. Oncometabolite Fingerprinting Using Fluorescent Single-Walled Carbon Nanotubes. Adv. Mater. Interfaces 2022, 9, 2101591 10.1002/admi.202101591.

[ref19] Kim M.; Chen C.; Wang P.; Mulvey J. J.; Yang Y.; Wun C.; Antman-Passig M.; Luo H.-B.; Cho S.; Long-Roche K.; Ramanathan L. V.; Jagota A.; Zheng M.; Wang Y.; Heller D. A. Detection of Ovarian Cancer via the Spectral Fingerprinting of Quantum-Defect-Modified Carbon Nanotubes in Serum by Machine Learning. Nat. Biomed. Eng. 2022, 6 (3), 267–275. 10.1038/s41551-022-00860-y. 35301449 PMC9108893

[ref20] Nißler R.; Bader O.; Dohmen M.; Walter S. G.; Noll C.; Selvaggio G.; Groß U.; Kruss S. Remote near Infrared Identification of Pathogens with Multiplexed Nanosensors. Nat. Commun. 2020, 11 (1), 5995 10.1038/s41467-020-19718-5. 33239609 PMC7689463

[ref21] Morrison K.; Tincher M.; Rothchild A.; Yehl K. Fingerprinting DNAzyme Cross-Reactivity for Pattern-Based Detection of Heavy Metals. Anal. Chem. 2024, 96 (29), 11780–11789. 10.1021/acs.analchem.4c01331. 39001810

[ref22] Ebrahim-Habibi M.-B.; Ghobeh M.; Mahyari F. A.; Rafii-Tabar H.; Sasanpour P. An Investigation into Non-Covalent Functionalization of a Single-Walled Carbon Nanotube and a Graphene Sheet with Protein G: A Combined Experimental and Molecular Dynamics Study. Sci. Rep. 2019, 9 (1), 1273 10.1038/s41598-018-37311-1. 30718580 PMC6362288

[ref23] Sultana N.; Dewey H. M.; Arellano A. G.; Budhathoki-Uprety J. Understanding the Molecular Assemblies of Single Walled Carbon Nanotubes and Tailoring Their Photoluminescence for the Next-Generation Optical Nanosensors. Chem. Mater. 2024, 36 (9), 4034–4053. 10.1021/acs.chemmater.4c00232.

[ref24] Lambert B. P.; Taheri A.; Wu S.-J.; Gillen A. J.; Kashaninejad M.; Boghossian A. A. Directed Evolution of Nanosensors for the Detection of Mycotoxins. bioRxiv 2023, 10.1101/2023.06.13.544576.

[ref25] An S.; Suh Y.; Kelich P.; Lee D.; Vukovic L.; Jeong S. Directed Evolution of Near-Infrared Serotonin Nanosensors with Machine Learning-Based Screening. Nanomaterials 2024, 14 (3), 247 10.3390/nano14030247. 38334518 PMC10856788

[ref26] Jeong S.; Yang D.; Beyene A. G.; Del Bonis-O’Donnell J. T.; Gest A. M. M.; Navarro N.; Sun X.; Landry M. P. High-Throughput Evolution of near-Infrared Serotonin Nanosensors. Sci. Adv. 2019, 5 (12), eaay3771 10.1126/sciadv.aay3771. 31897432 PMC6920020

[ref27] Conroy P. J.; Hearty S.; Leonard P.; O’Kennedy R. J. Antibody Production, Design and Use for Biosensor-Based Applications. Semin. Cell. Dev. Biol. 2009, 20 (1), 10–26. 10.1016/j.semcdb.2009.01.010. 19429487

[ref28] Yoon M.; Shin S.; Lee S.; Kang J.; Gong X.; Cho S.-Y. Scalable Photonic Nose Development through Corona Phase Molecular Recognition. ACS Sens. 2024, 9 (12), 6311–6319. 10.1021/acssensors.4c02327. 39630578

[ref29] Jolliffe I. T. Principal Component Analysis: A Beginner’s Guide — I. Introduction and Application. Weather 1990, 45 (10), 375–382. 10.1002/j.1477-8696.1990.tb05558.x.

[ref30] Bigdeli A.; Ghasemi F.; Golmohammadi H.; Abbasi-Moayed S.; Nejad M. A. F.; Fahimi-Kashani N.; Jafarinejad S.; Shahrajabian M.; Hormozi-Nezhad M. R. Nanoparticle-Based Optical Sensor Arrays. Nanoscale 2017, 9 (43), 16546–16563. 10.1039/C7NR03311G. 29083011

[ref31] Stork D. G.; Hart P. E.; D R. O. Pattern Classification; Wiley, 2000.

[ref32] Pourbahrami S.; Balafar M. A.; Khanli L. M.; Kakarash Z. A. A Survey of Neighborhood Construction Algorithms for Clustering and Classifying Data Points. Comput. Sci. Rev. 2020, 38, 100315 10.1016/j.cosrev.2020.100315.

[ref33] Okabe A.; Boots B.; Sugihara K. Nearest Neighbourhood Operations with Generalized Voronoi Diagrams: A Review. Int. J. Geogr. Inf. Syst. 1994, 8 (1), 43–71. 10.1080/02693799408901986.

[ref34] Coto-García A. M.; Sotelo-González E.; Fernández-Argüelles M. T.; Pereiro R.; Costa-Fernández J. M.; Sanz-Medel A. Nanoparticles as Fluorescent Labels for Optical Imaging and Sensing in Genomics and Proteomics. Anal. Bioanal. Chem. 2011, 399 (1), 29–42. 10.1007/s00216-010-4330-3. 21052647

[ref35] Ouyang M.; Huang J.-L.; Lieber C. M. Fundamental Electronic Properties and Applications of Single-Walled Carbon Nanotubes. Acc. Chem. Res. 2002, 35 (12), 1018–1025. 10.1021/ar0101685. 12484789

[ref36] Bachilo S. M.; Strano M. S.; Kittrell C.; Hauge R. H.; Smalley R. E.; Weisman R. B. Structure-Assigned Optical Spectra of Single-Walled Carbon Nanotubes. Science 2002, 298 (5602), 2361–2366. 10.1126/science.1078727. 12459549

[ref37] Kharlamova M. V.; Burdanova M. G.; Paukov M. I.; Kramberger C. Synthesis, Sorting, and Applications of Single-Chirality Single-Walled Carbon Nanotubes. Materials 2022, 15 (17), 5898 10.3390/ma15175898. 36079282 PMC9457432

[ref38] Jain A.; Homayoun A.; Bannister C. W.; Yum K. Single-walled Carbon Nanotubes as Near-infrared Optical Biosensors for Life Sciences and Biomedicine. Biotechnol. J. 2015, 10 (3), 447–459. 10.1002/biot.201400168. 25676253

[ref39] Nißler R.; Ackermann J.; Ma C.; Kruss S. Prospects of Fluorescent Single-Chirality Carbon Nanotube-Based Biosensors. Anal. Chem. 2022, 94 (28), 9941–9951. 10.1021/acs.analchem.2c01321. 35786856

[ref40] Zhang Y.; Guo J.; Tang Z.; Tang C.; Li Y.; Tao X.; Zhou B.; Chen W.; Guo L.; Tang K.; Liang T. Recent Developments and Trends of Biosensors Based on Carbon Nanotubes for Biomedical Diagnosis Applications: A Review. Biosens Bioelectron X 2024, 17, 100424 10.1016/j.biosx.2023.100424.

[ref41] Acharya R.; Patil T. V.; Dutta S. D.; Lee J.; Ganguly K.; Kim H.; Randhawa A.; Lim K.-T. Single-Walled Carbon Nanotube-Based Optical Nano/Biosensors for Biomedical Applications: Role in Bioimaging, Disease Diagnosis, and Biomarkers Detection. Adv. Mater. Technol. 2024, 9 (20), 2400279 10.1002/admt.202400279.

[ref42] Yaari Z.; Cheung J. M.; Baker H. A.; Frederiksen R. S.; Jena P. V.; Horoszko C. P.; Jiao F.; Scheuring S.; Luo M.; Heller D. A. Nanoreporter of an Enzymatic Suicide Inactivation Pathway. Nano Lett. 2020, 20 (11), 7819–7827. 10.1021/acs.nanolett.0c01858. 33119310 PMC8177003

[ref43] Kallmyer N. E.; Abdennadher M. S.; Agarwal S.; Baldwin-Kordick R.; Khor R. L.; Kooistra A. S.; Peterson E.; McDaniel M. D.; Reuel N. F. Inexpensive Near-Infrared Fluorimeters: Enabling Translation of NIR-Based Assays to the Field. Anal. Chem. 2021, 93 (11), 4800–4808. 10.1021/acs.analchem.0c03732. 33703890

[ref44] Dong J.; Lee M. A.; Rajan A. G.; Rahaman I.; Sun J. H.; Park M.; Salem D. P.; Strano M. S. A Synthetic Mimic of Phosphodiesterase Type 5 Based on Corona Phase Molecular Recognition of Single-Walled Carbon Nanotubes. Proc. Natl. Acad. Sci. U. S. A. 2020, 117 (43), 26616–26625. 10.1073/pnas.1920352117. 33055208 PMC7604511

[ref45] Wulf V.; Slor G.; Rathee P.; Amir R. J.; Bisker G. Dendron–Polymer Hybrids as Tailorable Responsive Coronae of Single-Walled Carbon Nanotubes. ACS Nano 2021, 15 (12), 20539–20549. 10.1021/acsnano.1c09125. 34878763

[ref46] Basu S.; Hendler-Neumark A.; Bisker G. Rationally Designed Functionalization of Single-Walled Carbon Nanotubes for Real-Time Monitoring of Cholinesterase Activity and Inhibition in Plasma. Small 2024, 20, 2309481 10.1002/smll.202309481. 38358018

[ref47] Basu S.; Hendler-Neumark A.; Bisker G. Monitoring Enzyme Activity Using Near-Infrared Fluorescent Single-Walled Carbon Nanotubes. ACS Sens. 2024, 9 (5), 2237–2253. 10.1021/acssensors.4c00377. 38669585 PMC11129355

[ref48] Williams R. M.; Harvey J. D.; Budhathoki-Uprety J.; Heller D. A. Glutathione-S-Transferase Fusion Protein Nanosensor. Nano Lett. 2020, 20 (10), 7287–7295. 10.1021/acs.nanolett.0c02691. 32955895 PMC8266418

[ref49] Ehrlich R.; Hendler-Neumark A.; Wulf V.; Amir D.; Bisker G. Optical Nanosensors for Real-Time Feedback on Insulin Secretion by Β-Cells. Small 2021, 17 (30), 2101660 10.1002/smll.202101660. 34197026

[ref50] Pinals R. L.; Ledesma F.; Yang D.; Navarro N.; Jeong S.; Pak J. E.; Kuo L.; Chuang Y.-C.; Cheng Y.-W.; Sun H.-Y.; Landry M. P. Rapid SARS-CoV-2 Spike Protein Detection by Carbon Nanotube-Based Near-Infrared Nanosensors. Nano Lett. 2021, 21 (5), 2272–2280. 10.1021/acs.nanolett.1c00118. 33635655 PMC10493163

[ref51] Gillen A. J.; Siefman D. J.; Wu S.-J.; Bourmaud C.; Lambert B.; Boghossian A. A. Templating Colloidal Sieves for Tuning Nanotube Surface Interactions and Optical Sensor Responses. J. Colloid Interface Sci. 2020, 565, 55–62. 10.1016/j.jcis.2019.12.058. 31931299

[ref52] Beyene A. G.; Delevich K.; Del Bonis-O’Donnell J. T.; Piekarski D. J.; Lin W. C.; Thomas A. W.; Yang S. J.; Kosillo P.; Yang D.; Prounis G. S.; Wilbrecht L.; Landry M. P. Imaging Striatal Dopamine Release Using a Nongenetically Encoded near Infrared Fluorescent Catecholamine Nanosensor. Sci. Adv. 2019, 5 (7), eaaw3108 10.1126/sciadv.aaw3108. 31309147 PMC6620097

[ref53] Dinarvand M.; Elizarova S.; Daniel J.; Kruss S. Imaging of Monoamine Neurotransmitters with Fluorescent Nanoscale Sensors. ChemPluschem 2020, 85 (7), 1465–1480. 10.1002/cplu.202000248. 32644301

[ref54] Lee M. A.; Wang S.; Jin X.; Bakh N. A.; Nguyen F. T.; Dong J.; Silmore K. S.; Gong X.; Pham C.; Jones K. K.; Muthupalani S.; Bisker G.; Son M.; Strano M. S. Implantable Nanosensors for Human Steroid Hormone Sensing In Vivo Using a Self-Templating Corona Phase Molecular Recognition. Adv. Healthcare Mater. 2020, 9 (21), 2000429 10.1002/adhm.202000429. 32940022

[ref55] Safaee M. M.; Gravely M.; Roxbury D. A Wearable Optical Microfibrous Biomaterial with Encapsulated Nanosensors Enables Wireless Monitoring of Oxidative Stress. Adv. Funct. Mater. 2021, 31 (13), 2006254 10.1002/adfm.202006254.

[ref56] Wu H.; Nißler R.; Morris V.; Herrmann N.; Hu P.; Jeon S.-J.; Kruss S.; Giraldo J. P. Monitoring Plant Health with Near-Infrared Fluorescent H _2_ O _2_ Nanosensors. Nano Lett. 2020, 20 (4), 2432–2442. 10.1021/acs.nanolett.9b05159. 32097014

[ref57] Hofferber E. M.; Stapleton J. A.; Iverson N. M. Review—Single Walled Carbon Nanotubes as Optical Sensors for Biological Applications. J. Electrochem. Soc. 2020, 167 (3), 037530 10.1149/1945-7111/ab64bf.

[ref58] Farrera C.; Torres Andón F.; Feliu N. Carbon Nanotubes as Optical Sensors in Biomedicine. ACS Nano 2017, 11 (11), 10637–10643. 10.1021/acsnano.7b06701. 29087693

[ref59] Hendler-Neumark A.; Wulf V.; Bisker G. In Vivo Imaging of Fluorescent Single-Walled Carbon Nanotubes within C. Elegans Nematodes in the near-Infrared Window. Mater. Today Bio. 2021, 12, 100175 10.1016/j.mtbio.2021.100175. PMC8649898 34927042

[ref60] Nandi S.; Caicedo K.; Cognet L. When Super-Resolution Localization Microscopy Meets Carbon Nanotubes. Nanomaterials 2022, 12 (9), 1433 10.3390/nano12091433. 35564142 PMC9105540

[ref61] Kleiner S.; Wulf V.; Bisker G. Single-Walled Carbon Nanotubes as near-Infrared Fluorescent Probes for Bio-Inspired Supramolecular Self-Assembled Hydrogels. J. Colloid Interface Sci. 2024, 670, 439–448. 10.1016/j.jcis.2024.05.098. 38772260

[ref62] Ackermann J.; Metternich J. T.; Herbertz S.; Kruss S. Biosensing with Fluorescent Carbon Nanotubes. Angew. Chem., Int. Ed. 2022, 61 (18), e202112372 10.1002/anie.202112372. PMC9313876 34978752

[ref63] De Los Santos Z. A.; Lin Z.; Zheng M. Optical Detection of Stereoselective Interactions with DNA-Wrapped Single-Wall Carbon Nanotubes. J. Am. Chem. Soc. 2021, 143 (49), 20628–20632. 10.1021/jacs.1c11372. 34843644

[ref64] Blanch A. J.; Lenehan C. E.; Quinton J. S. Optimizing Surfactant Concentrations for Dispersion of Single-Walled Carbon Nanotubes in Aqueous Solution. J. Phys. Chem. B 2010, 114 (30), 9805–9811. 10.1021/jp104113d. 20666522

[ref65] Gerstman E.; Hendler-Neumark A.; Wulf V.; Bisker G. Monitoring the Formation of Fibrin Clots as Part of the Coagulation Cascade Using Fluorescent Single-Walled Carbon Nanotubes. ACS Appl. Mater. Interfaces 2023, 15 (18), 21866–21876. 10.1021/acsami.3c00828. 37128896 PMC10176323

[ref66] Budhathoki-Uprety J.; Harvey J. D. D.; Isaac E.; Williams R. M. M.; Galassi T. V. V.; Langenbacher R. E. E.; Heller D. A. A. Polymer Cloaking Modulates the Carbon Nanotube Protein Corona and Delivery into Cancer Cells. J. Mater. Chem. B 2017, 5 (32), 6637–6644. 10.1039/C7TB00695K. 32264426

[ref67] Fernandes R. M. F.; Dai J.; Regev O.; Marques E. F.; Furó I. Block Copolymers as Dispersants for Single-Walled Carbon Nanotubes: Modes of Surface Attachment and Role of Block Polydispersity. Langmuir 2018, 34 (45), 13672–13679. 10.1021/acs.langmuir.8b02658. 30335395

[ref68] Antonucci A.; Kupis-Rozmysłowicz J.; Boghossian A. A. Noncovalent Protein and Peptide Functionalization of Single-Walled Carbon Nanotubes for Biodelivery and Optical Sensing Applications. ACS Appl. Mater. Interfaces 2017, 9 (13), 11321–11331. 10.1021/acsami.7b00810. 28299937

[ref69] Tsyboulski D. A.; Bakota E. L.; Witus L. S.; Rocha J.-D. R.; Hartgerink J. D.; Weisman R. B. Self-Assembling Peptide Coatings Designed for Highly Luminescent Suspension of Single-Walled Carbon Nanotubes. J. Am. Chem. Soc. 2008, 130 (50), 17134–17140. 10.1021/ja807224x. 19053447 PMC2639792

[ref70] Heller D. A.; Pratt G. W.; Zhang J.; Nair N.; Hansborough A. J.; Boghossian A. A.; Reuel N. F.; Barone P. W.; Strano M. S. Peptide Secondary Structure Modulates Single-Walled Carbon Nanotube Fluorescence as a Chaperone Sensor for Nitroaromatics. Proc. Natl. Acad. Sci. U. S. A. 2011, 108 (21), 8544–8549. 10.1073/pnas.1005512108. 21555544 PMC3102399

[ref71] Matsukawa Y.; Umemura K. Chirality Luminescent Properties of Single-Walled Carbon Nanotubes during Redox Reactions. Opt. Mater. 2021, 112, 110748 10.1016/j.optmat.2020.110748.

[ref72] Salem D. P.; Landry M. P.; Bisker G.; Ahn J.; Kruss S.; Strano M. S. Chirality Dependent Corona Phase Molecular Recognition of DNA-Wrapped Carbon Nanotubes. Carbon 2016, 97, 147–153. 10.1016/j.carbon.2015.08.075.

[ref73] Choi J. H.; Strano M. S. Solvatochromism in Single-Walled Carbon Nanotubes. Appl. Phys. Lett. 2007, 90 (22), 88–91. 10.1063/1.2745228.

[ref74] Järup L. Hazards of Heavy Metal Contamination. Br. Med. Bull. 2003, 68 (1), 167–182. 10.1093/bmb/ldg032. 14757716

[ref75] Barnham K. J.; Bush A. I. Biological Metals and Metal-Targeting Compounds in Major Neurodegenerative Diseases. Chem. Soc. Rev. 2014, 43, 6727 10.1039/C4CS00138A. 25099276

[ref76] Tofan L.; Wenkert R. Chelating Polymers with Valuable Sorption Potential for Development of Precious Metal Recycling Technologies. Rev. Chem. Eng. 2022, 38 (2), 167–183. 10.1515/revce-2019-0075.

[ref77] Guo S.-Y.; Hou P.-X.; Zhang F.; Liu C.; Cheng H.-M. Gas Sensors Based on Single-Wall Carbon Nanotubes. Molecules 2022, 27 (17), 5381 10.3390/molecules27175381. 36080149 PMC9458085

[ref78] Gong X.; Cho S.-Y.; Kuo S.; Ogunlade B.; Tso K.; Salem D. P.; Strano M. S. Divalent Metal Cation Optical Sensing Using Single-Walled Carbon Nanotube Corona Phase Molecular Recognition. Anal. Chem. 2022, 94 (47), 16393–16401. 10.1021/acs.analchem.2c03648. 36378652

[ref79] Heller D. A.; Jeng E. S.; Yeung T.-K.; Martinez B. M.; Moll A. E.; Gastala J. B.; Strano M. S. Optical Detection of DNA Conformational Polymorphism on Single-Walled Carbon Nanotubes. Science 2006, 311 (5760), 508–511. 10.1126/science.1120792. 16439657

[ref80] Jin H.; Jeng E. S.; Heller D. A.; Jena P. V.; Kirmse R.; Langowski J.; Strano M. S. Divalent Ion and Thermally Induced DNA Conformational Polymorphism on Single-Walled Carbon Nanotubes. Macromolecules 2007, 40 (18), 6731–6739. 10.1021/ma070608t.

[ref81] Gillen A. J.; Kupis-Rozmysłowicz J.; Gigli C.; Schuergers N.; Boghossian A. A. Xeno Nucleic Acid Nanosensors for Enhanced Stability Against Ion-Induced Perturbations. J. Phys. Chem. Lett. 2018, 9 (15), 4336–4343. 10.1021/acs.jpclett.8b01879. 30004705

[ref82] Andjelkovic M.; Vancamp J.; Demeulenaer B.; Depaemelaere G.; Socaciu C.; Verloo M.; Verhe R. Iron-Chelation Properties of Phenolic Acids Bearing Catechol and Galloyl Groups. Food Chem. 2006, 98 (1), 23–31. 10.1016/j.foodchem.2005.05.044.

[ref83] Irankunda R.; Camaño Echavarría J. A.; Paris C.; Stefan L.; Desobry S.; Selmeczi K.; Muhr L.; Canabady-Rochelle L. Metal-Chelating Peptides Separation Using Immobilized Metal Ion Affinity Chromatography: Experimental Methodology and Simulation. Separations 2022, 9 (11), 370 10.3390/separations9110370.

[ref84] Yang M.; Song W. J. Diverse Protein Assembly Driven by Metal and Chelating Amino Acids with Selectivity and Tunability. Nat. Commun. 2019, 10 (1), 5545 10.1038/s41467-019-13491-w. 31804480 PMC6895169

[ref85] Tournus F.; Latil S.; Heggie M. I.; Charlier J.-C. π-Stacking Interaction between Carbon Nanotubes and Organic Molecules. Phys. Rev. B 2005, 72 (7), 075431 10.1103/PhysRevB.72.075431.

[ref86] Kruss S.; Landry M. P.; Vander Ende E.; Lima B. M. A.; Reuel N. F.; Zhang J.; Nelson J.; Mu B.; Hilmer A.; Strano M. Neurotransmitter Detection Using Corona Phase Molecular Recognition on Fluorescent Single-Walled Carbon Nanotube Sensors. J. Am. Chem. Soc. 2014, 136 (2), 713–724. 10.1021/ja410433b. 24354436

[ref87] Joshua Ashaolu T.; Lee C. C.; Opeolu Ashaolu J.; Pourjafar H.; Jafari S. M. Metal-Binding Peptides and Their Potential to Enhance the Absorption and Bioavailability of Minerals. Food Chem. 2023, 428, 136678 10.1016/j.foodchem.2023.136678. 37418874

[ref88] Ren X.; Zou Q.; Yuan C.; Chang R.; Xing R.; Yan X. The Dominant Role of Oxygen in Modulating the Chemical Evolution Pathways of Tyrosine in Peptides: Dityrosine or Melanin. Angew. Chem., Int. Ed. 2019, 131 (18), 5930–5934. 10.1002/ange.201814575. 30666757

[ref89] Reid L. O.; Vignoni M.; Martins-Froment N.; Thomas A. H.; Dántola M. L. Photochemistry of Tyrosine Dimer: When an Oxidative Lesion of Proteins Is Able to Photoinduce Further Damage. Photochem. Photobiol. Sci. 2019, 18 (7), 1732–1741. 10.1039/c9pp00182d. 31070216

[ref90] Lampel A.; McPhee S. A.; Kassem S.; Sementa D.; Massarano T.; Aramini J. M.; He Y.; Ulijn R. V. Melanin-Inspired Chromophoric Microparticles Composed of Polymeric Peptide Pigments. Angew. Chem., Int. Ed. 2021, 60 (14), 7564–7569. 10.1002/anie.202015170. 33432673

[ref91] Settele S.; Schrage C. A.; Jung S.; Michel E.; Li H.; Flavel B. S.; Hashmi A. S. K.; Kruss S.; Zaumseil J. Ratiometric Fluorescent Sensing of Pyrophosphate with Sp^3^-Functionalized Single-Walled Carbon Nanotubes. Nat. Commun. 2024, 15 (1), 706 10.1038/s41467-024-45052-1. 38267487 PMC10808354

[ref92] Pan J.; Li F.; Choi J. H. Single-Walled Carbon Nanotubes as Optical Probes for Bio-Sensing and Imaging. J. Mater. Chem. B 2017, 5 (32), 6511–6522. 10.1039/C7TB00748E. 32264414

[ref93] Wulf V.; Bichachi E.; Hendler-Neumark A.; Massarano T.; Leshem A. B.; Lampel A.; Bisker G. Multicomponent System of Single-Walled Carbon Nanotubes Functionalized with a Melanin-Inspired Material for Optical Detection and Scavenging of Metals. Adv. Funct. Mater. 2022, 32 (49), 2209688 10.1002/adfm.202209688.

[ref94] Barone P. W.; Baik S.; Heller D. A.; Strano M. S. Near-Infrared Optical Sensors Based on Single-Walled Carbon Nanotubes. Nat. Mater. 2005, 4 (1), 86–92. 10.1038/nmat1276. 15592477

[ref95] O’Connell M. J.; Eibergen E. E.; Doorn S. K. Chiral Selectivity in the Charge-Transfer Bleaching of Single-Walled Carbon-Nanotube Spectra. Nat. Mater. 2005, 4 (5), 412–418. 10.1038/nmat1367. 15821741

[ref96] Satishkumar B. C.; Brown L. O.; Gao Y.; Wang C.-C.; Wang H.-L.; Doorn S. K. Reversible Fluorescence Quenching in Carbon Nanotubes for Biomolecular Sensing. Nat. Nanotechnol. 2007, 2 (9), 560–564. 10.1038/nnano.2007.261. 18654368

[ref97] O’Connell M. J.; Bachilo S. M.; Huffman C. B.; Moore V. C.; Strano M. S.; Haroz E. H.; Rialon K. L.; Boul P. J.; Noon W. H.; Kittrell C.; Ma J.; Hauge R. H.; Weisman R. B.; Smalley R. E. Band Gap Fluorescence from Individual Single-Walled Carbon Nanotubes. Science 2002, 297 (5581), 593–596. 10.1126/science.1072631. 12142535

[ref98] Mardia K. V.; Kent J. T.; Taylor C. C.; Multivariate Analysis, 2nd Edition. In Wiley Series in Probability and Statistics; John Wiley & Sons: Nashville, TN, 2024; Vol. 88.

[ref99] Kuhn H. W. The Hungarian Method for the Assignment Problem. Nav. Res. Logist. Q 1955, 2 (1–2), 83–97. 10.1002/nav.3800020109.

[ref100] Munkres J. Algorithms for the Assignment and Transportation Problems. J. Soc. Ind. Appl. Math. 1957, 5 (1), 32–38. 10.1137/0105003.

[ref101] Rand W. M. Objective Criteria for the Evaluation of Clustering Methods. J. Am. Stat. Assoc. 1971, 66 (336), 846–850. 10.1080/01621459.1971.10482356.

[ref102] Liu H.; Setiono R. Discretization Of Ordinal Attributes And Feature Selection DSpace software 1995 1–18

[ref103] Too J.; Abdullah A. R. A New and Fast Rival Genetic Algorithm for Feature Selection. J. Supercomput. 2021, 77 (3), 2844–2874. 10.1007/s11227-020-03378-9.

[ref104] Cai J.; Luo J.; Wang S.; Yang S. Feature Selection in Machine Learning: A New Perspective. Neurocomputing 2018, 300, 70–79. 10.1016/j.neucom.2017.11.077.

