# Supplementary material for: Metal-Ion Optical Fingerprinting Sensor Selection via an Analyte Classification and Feature Selection Algorithm
Source: Anal Chem. 2025 Mar 27;97(16):8821–32. doi: 10.1021/acs.analchem.4c06762 (PMC12044593; doi:10.1021/acs.analchem.4c06762)
Supplement: Supplementary file 1 — ac4c06762_si_001.pdf [file ac4c06762_si_001.pdf]

-Supporting Information-

Metal-ion Optical Fingerprinting Sensor Selection *via* an Analyte Classification and Feature Selection Algorithm

Gabriel Petresky,[a] Michael Faran,[a] Verena Wulf,[a] and Gili Bisker[*,a,b,c,d]*

[a]Department of Biomedical Engineering, Faculty of Engineering, Tel Aviv University, Tel Aviv 6997801, Israel

[b]Center for Physics and Chemistry of Living Systems, Tel Aviv University, Tel Aviv 6997801, Israel

[c]Center for Nanoscience and Nanotechnology, Tel Aviv University, Tel Aviv 6997801, Israel

[d]Center for Light-Matter Interaction, Tel Aviv University, Tel Aviv 6997801, Israel

*bisker@tauex.tau.ac.il

Table of Contents

SECTION I: SUPPORTING EXPERIMENTAL SECTION 5

PEPTIDE FUNCTIONALIZATION: 5

ABSORPTION AND FLUORESCENCE SPECTROSCOPY OF PEPTIDE OXIDATION. 5

NIR FLUORESCENCE SPECTROSCOPY OF SWCNTS. 5

NIR FLUORESCENCE RESPONSE TO METAL-IONS. 6

RAMAN SPECTROSCOPY: 6

NIR FLUORESCENCE RESPONSE IN SERUM: 6

NIR FLUORESCENCE RESPONSE IN MINERAL WATER: 6

SECTION II: ADDITIONAL SCHEMES AND FIGURES..............................7

Figure S1: Molecular structures of the Fmoc-peptides... 7

Figure S2: Effect of FFFF chain on SWCNT suspension stability....................................... 8

Figure S3: Absorption spectra of the SWCNT-peptide suspensions diluted 1:20 in water... 9

Figure S4: Excitation emission maps of the SWCNT-peptide suspensions before (top) and after (bottom) oxidation. 10

Figure S5: Absorption and fluorescence spectra of the SWCNT-peptides before and after oxidation. 11

Figure S6: Raman spectra of the SWCNTs-peptides, before (blue) and after (red) oxidation, ... 13

Figure S7: TEM of SWCNT-Lys.. 14

Figure S8: TEM of SWCNT-Cys.. 14

Figure S9: Relative fluorescence response of the SWCNT-peptide sensors to each metal at 300 µM for three different chiralities. 15

Figure S10: Relative fluorescence response of the SWCNT-peptide sensors to each metal at 300 µM for three different chiralities with native and oxidized sensors appearing next to each other.. 16

Figure S11: Relative fluorescence response of the SWCNT-peptide sensors to each metal at 300 µM for three different chiralities, ... 17

Figure S12: Correlation of the absolute relative fluorescence response of the SWCNT-peptide sensors to the peptide surface coverage or the zeta potential................................. 18

SECTION III: THE ANALYTE CLASSIFICATION AND FEATURE SELECTION ALGORITHM (ACFSA) 19

INTRODUCTION 19
1. THE ACFSA FLOWCHART 19
Scheme S1: Flowchart of the ACFSA process.. 21
1.1. INPUT 21
1.2. THE CLUSTERING ALGORITHM 22
1.3. THE CLASSIFICATION EVALUATION 22
1.4. NEAREST NEIGHBOR CLASSIFIER 23
1.5. BACKWARD FEATURE SELECTION ALGORITHM 24
1.6. NEAREST NEIGHBOR FINAL CLASSIFIER 24
1.7. 1D ACFSA 25
1.8. WORKING POINT SELECTION 25
2. TIME COMPLEXITY ANALYSIS 25
Dominant Term Analysis: 26
3. ACFSA IMPLEMENTATION WITH SIMULATED DATA 27
Figure S13: The ACFSA result for all the SWCNT-peptide sensor data for the artificial dataset, containing more samples and a standard deviation (STD) increased fourfold for each cluster... 27
4. ACFSA WITH RANDOMIZED FEATURE SELECTION 28
Figure S14: The ACFSA result for all the SWCNT-peptide sensors, with a random feature elimination scheme, instead of the Chi-squared method. 29
Figure S15: ARI versus the remaining iteration number for all sensors included configuration. 30
5. ACFSA IMPLEMENTATION WITH EXPERIMENTAL DATA 30
Table S1: ACFSA-selected sensor sets for different input data subsets of SWCNT-peptides. 31
Figure S16: The ACFSA results for all the SWCNT-peptide sensors................................ 33
Figure S17: The ACFSA results for the oxidized SWCNT-peptide sensors. 34
Figure S18: The ACFSA results for the non-oxidized SWCNT-peptide sensors............... 35
Figure S19: The ACFSA results for the (6,5) chirality SWCNT-peptide sensors.............. 36
Figure S20: The ACFSA results for the (7,5) chirality SWCNT-peptide sensors.............. 37
Figure S21: The ACFSA results for the (9,4) chirality SWCNT-peptide sensors. 38
6. PEARSON CORRELATION COEFFICIENTS FOR THE INTERCLUSTER DISTANCE WITH THE CLASSIFIER ERROR, AND FOR THE ARI WITH THE CLASSIFIER ERROR 39
Table S2: Pearson correlation coefficients for the intercluster distance with the classifier error $\rho(D, error)$, and for the ARI with the classifier error ($\rho(ARI, error)$). For all sensor

configurations, the ARI is 1 for all the iteration numbers, so the Pearson coefficient is not defined........ 39

7. THE ACFSA ALGORITHM ON A BINARY RESPONSE SET 40

Figure S22: Binary principal component analysis and classifier........ 41

SECTION IV: LIMIT OF DETECTION AND SENSOR RESPONSE IN SERUM AND MINERAL WATER........42

LIMIT OF DETECTION FOR SWCNT-GLY-(6,5)........ 42

Figure S23: Relative fluorescent response of the sensor SWCNT-Gly-(6,5) vs. a range of metal-ion concentrations........ 43

SENSOR PERFORMANCE IN MINERAL WATER AND SERUM 44

Figure S24: Relative fluorescence response of the sensor SWCNT-Gly-(6,5) to the five metal-ions........ 44

SECTION V: SENSOR STABILITY AND BATCH VARIATIONS........45

Figure S25: Batch-to-batch sensor variation........ 45

Figure S27: SWCNT-Gly response after 6 months. 47

Figure S28: Suspension stability after 6 months........ 48

REFERENCES........49

Section I: Supporting Experimental Section

Peptide functionalization:

For complete peptide dissolution, Fmoc-FFFFYEYEY and Fmoc-FFFFYGYGY required the addition of sodium hydroxide (0.5 M, approximately 50 µL). Fmoc-FFFFYRYRY was first dissolved in 50 µL of DMSO, then 950 µL of distilled water was added, whereas Fmoc-FFFFYCYCY was dissolved in 200 µL of DMSO, and then 800 µL of water and 20 µL of 0.5 M NaOH were added to complete dissolution. Fmoc-FFFFYKYKY was dissolved in water without any additions. For the suspension of Fmoc-YKYKY, we followed the same protocol as Fmoc-FFFFYKYKY with mass adjusted to maintain molarity. For the suspension of SWCNT-SDS, the SWCNTs were suspended in 2% SDS (Bio-lab Ltd.), sonicated, washed, and resuspended with 2% SDS. Suspensions were stored at concentrations of 50 to 200 mg L^{-1}. Before experimentation, the suspensions were freshly diluted to a working concentration of 1 mg L^{-1} and allowed to stabilize for 30 minutes.

Absorption and fluorescence spectroscopy of peptide oxidation.

The SWCNT-peptide fluorescence and absorption measurements following the peptide oxidation were recorded using a plate reader (Fusion Optics Reader Platform SPARK, Tecan). Fluorescence measurements were performed with two different filter sets, λ_{ex} = 280 nm and λ_{em} = 300–600 nm; and λ_{ex} = 320 nm and λ_{em} = 350–600 nm. To show the coloration of the Fmoc-peptides before and after oxidation, the SWCNT-peptides were lyophilized, and the peptides were then resuspended in DMSO while the SWCNTs remained in the precipitate.

NIR fluorescence spectroscopy of SWCNTs.

Fluorescence emission spectra were recorded in a 96-well plate mounted on an inverted microscope (Olympus IX73). A super-continuum white light laser (NKT-photonics, Super-K Extreme) with a bandwidth filter (NKT-photonics, Super-K varia, $\Delta\lambda$ = 20 nm) was coupled into the microscope as the excitation source with a laser intensity of 20 mW. Fluorescence emission was spectrally resolved using a spectrograph (Spectra Pro HRS-300, Teledyne Princeton Instruments) with a slit-width of 500 µm and a grating (150 g mm^{-1}). The fluorescence intensity spectrum was recorded by an InGaAs-detector (PylonIR, Teledyne Princeton Instruments).

Excitation-emission maps were recorded using an excitation wavelength range of 500 nm to 840 nm in 2 nm steps.

NIR fluorescence response to metal-ions.

Copper(II) chloride ($CuCl_2$), Nickel(II) chloride ($NiCl_2$), Chromium(III) chloride ($CrCl_3$), Lead(II) chloride ($PbCl_2$), and Mercury(II) chloride ($HgCl_2$) were purchased from Sigma-Aldrich. For the measurements, aliquots of 147 µL of 1 mg L^{-1} SWCNT-peptides in water were placed in a 96-well plate and treated with 3 µL metal-ion solution in water to obtain a final metal-ion concentration of 300 µM or with 3 µL water as a control. The NIR-fluorescence emission spectra of the SWCNT-peptides were measured after an incubation time of 15 min at an excitation wavelength of λ = 570 nm, 660 nm, and 730 nm, with an intensity of around 20 mW, corresponding to the excitation resonance of the (6,5), (7,5), and (9,4) chiralities of the SWCNTs, respectively.

Raman spectroscopy:

Raman spectra were acquired using a confocal micro-Raman (PL) spectrometer (LabRam HR Evolution). Samples of 20 mg L^{-1} of the ten SWCNTs-peptide suspensions were placed on a 1 cm path cuvette and excited with a 532 nm laser. Measurements were taken with a ×100 objective lens at a laser power of 100 mW for 3 seconds. Water was used as blank.

NIR fluorescence response in serum:

Aliquots of 147 µL solution containing 5% fetal bovine serum (FBS, Biowest, USA) and 1 mg L^{-1} of SWCNT-Gly sensor were mixed with 3 µL of 30 mM metal-ion chlorides, Cu^{2+}, Ni^{2+}, Cr^{3+}, Pb^{2+}, and Hg^{2+}.

NIR fluorescence response in mineral water:

Aliquots of 147 µL solution of commercial mineral water (Neviot, Israel) and 1 mg L^{-1} of SWCNT-Gly sensor were mixed with 3 µL of 15 mM metal-ion chlorides, Cu^{2+}, Ni^{2+}, Cr^{3+}, Pb^{2+}, and Hg^{2+}.

Section II: Additional Schemes and Figures

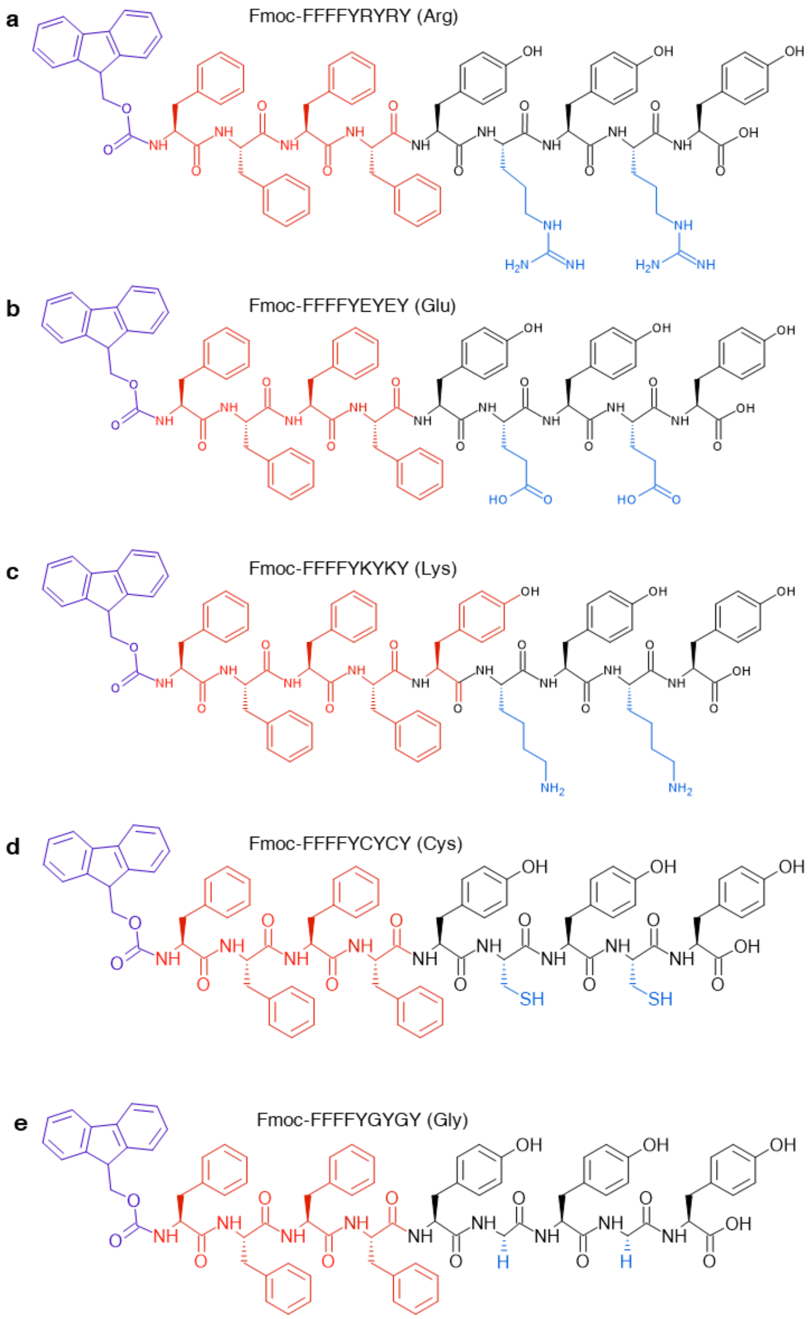

Figure S1: Molecular structures of the Fmoc-peptides. Fmoc-group (purple), phenylalanine (F) chain (red), tyrosine (black), variable amino acid (blue). a) Fmoc-FFFFYRYRY (R: Arginine), b) Fmoc-FFFFYEYEY (E: Glutamic acid), c) Fmoc-FFFFYKYKY (K: Lysine), d) Fmoc-FFFFYCYCY (C: Cysteine), e) Fmoc-FFFFYGYGY (G: Glycine).

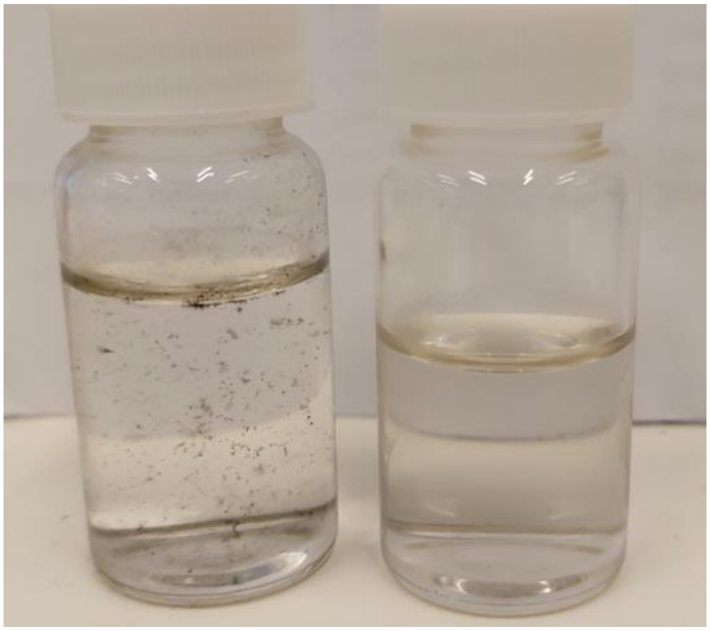

Figure S2: Effect of FFFF chain on SWCNT suspension stability. Left: Aggregated suspension of SWCNT-Fmoc-YKYKY (1 mg L^{-1}), indicating poor dispersion. Right: Stable suspension of SWCNT-Fmoc-FFFF-YKYKY (1 mg L^{-1}), demonstrating that the incorporation of the FFFF sequence enhances peptide attachment to SWCNTs, preventing aggregation and maintaining dispersion stability.

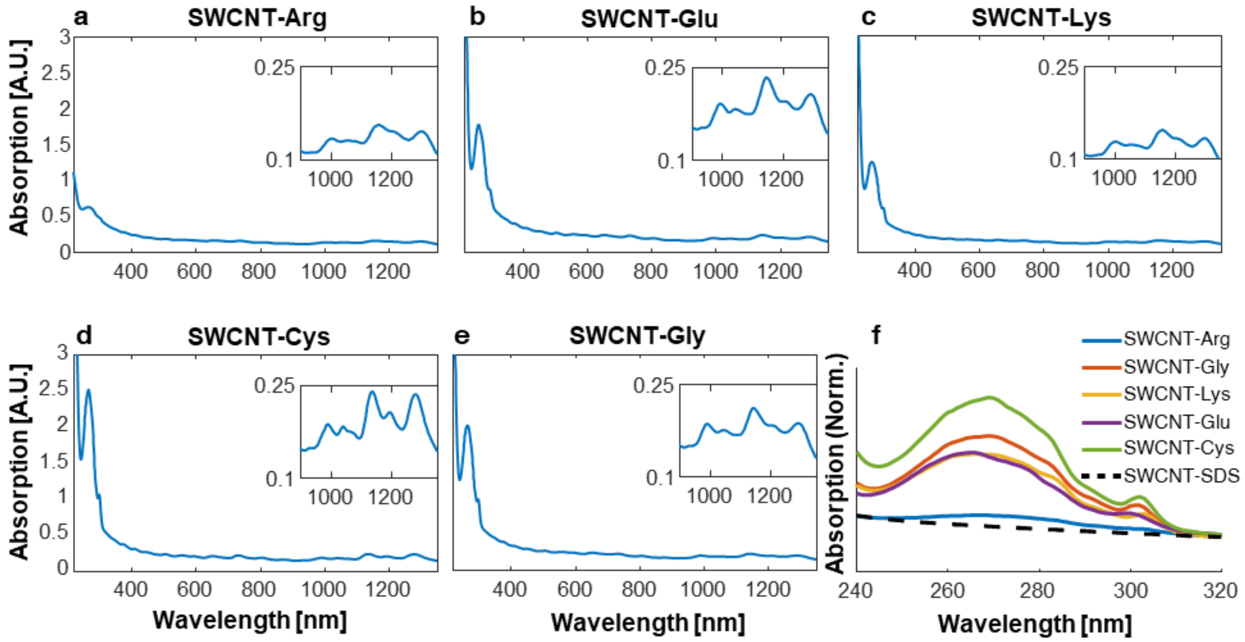

Figure S3: Absorption spectra of the SWCNT-peptide suspensions diluted 1:20 in water. a) SWCNT-Arg, b) SWCNT-Glu, c) SWCNT-Lys, d) SWCNT-Cys, e) SWCNT-Gly. Insets: NIR absorption of the SWCNTs. f) Comparison of the Fmoc-absorption peaks between 260-280 nm at a constant SWCNT concentration (through spectra normalization at 632 nm), of SWCNT-Arg (light blue), SWCNT-Glu (orange), SWCNT-Lys (yellow), SWCNT-Cys (purple), and SWCNT-Gly (green). The absorption relative to SWCNT-Arg, at 270 nm, subtracting the SWCNT-SDS background, provides an estimation of peptide functionalization load: SWCNT-Arg: 1.00, SWCNT-Gly: 9.09, SWCNT-Lys: 6.84, SWCNT-Glu: 7.02, and SWCNT-Cys: 12.20. Quantification estimate for peptide load was done using Fmoc-FFFFYEYEY as a reference, under the assumption of similar UV absorption and molecular weight for all peptides, yielding a load range of 294 mg L^{-1} for SWCNT-Arg up to 3580 mg L^{-1} for SWCNT-Cys, with an approximate SWCNT concentration of 100 mg L^{-1}.

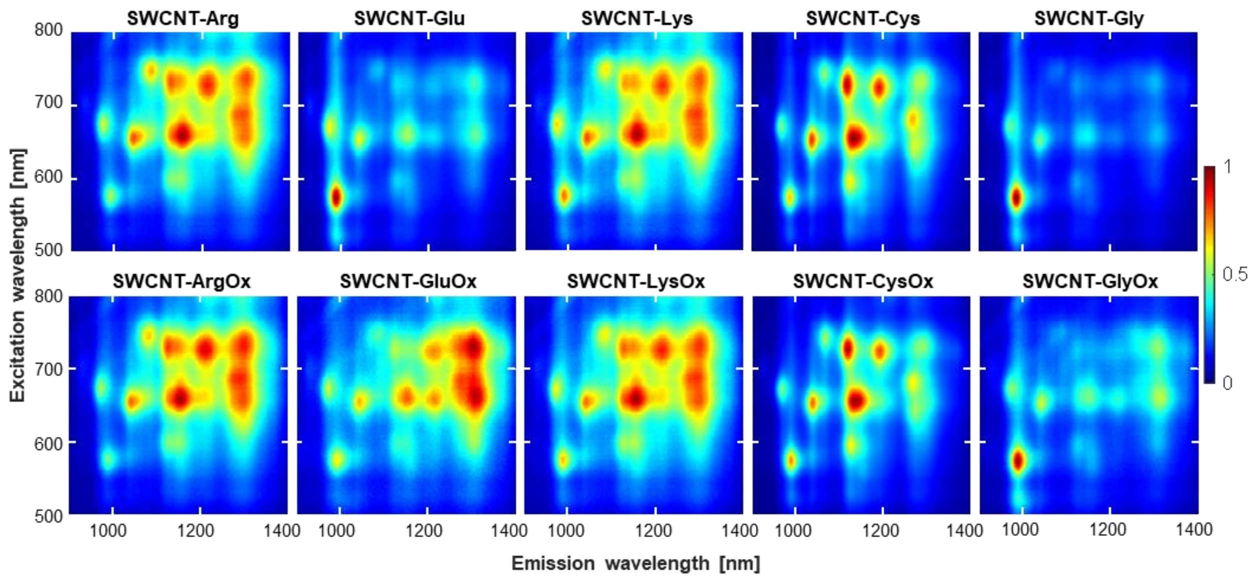

Figure S4: Excitation emission maps of the SWCNT-peptide suspensions before (top) and after (bottom) oxidation.

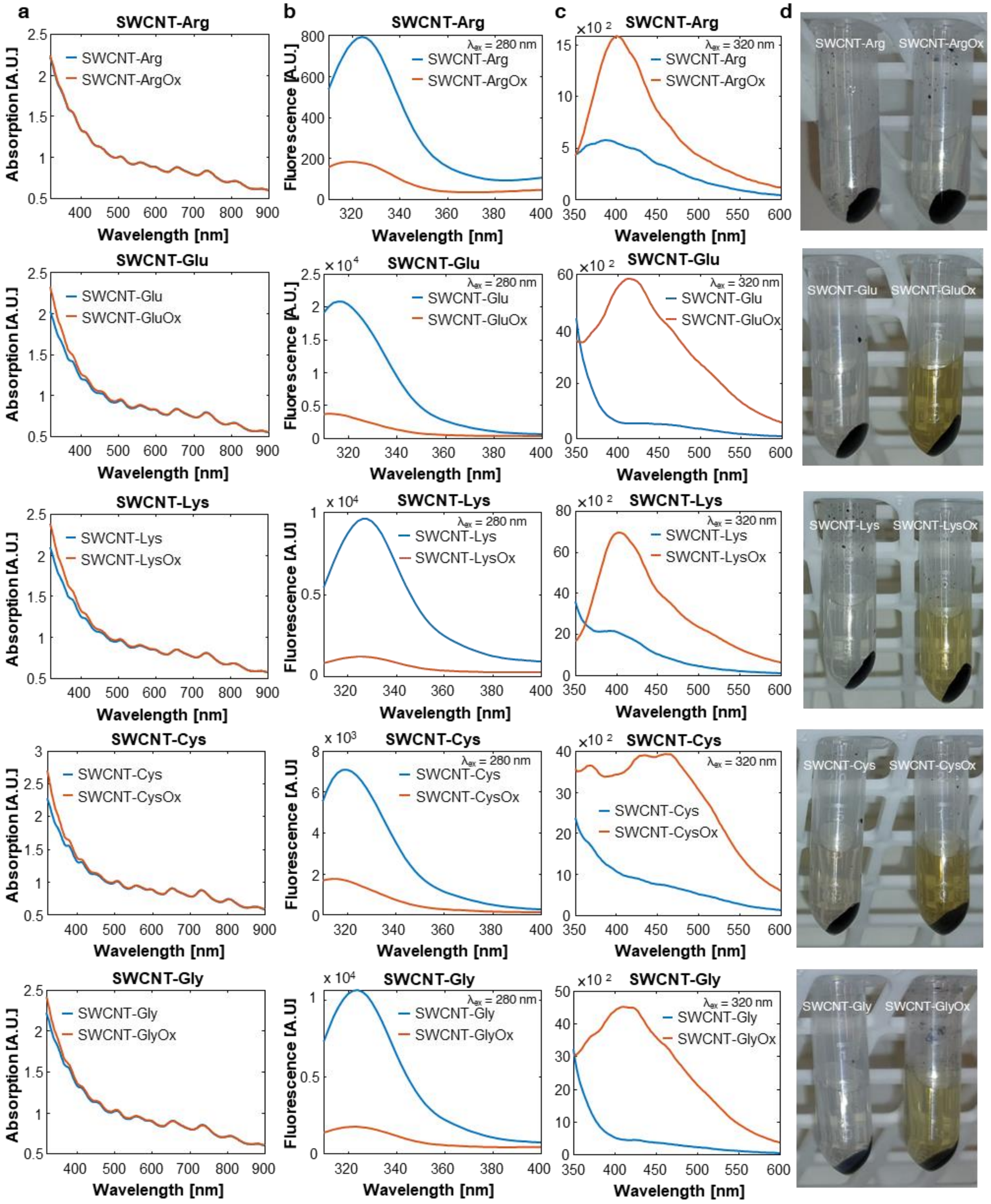

Figure S5: Absorption and fluorescence spectra of the SWCNT-peptides before and after oxidation. a) Absorption spectra of the SWCNT-peptide suspensions before (blue) and after (orange) oxidation. b) Decrease in fluorescence intensity under 280 nm excitation before (blue)

and after (orange) oxidation. c) Increase in fluorescence intensity under 320 nm excitation before (blue) and after (orange) oxidation. d) Coloration of the peptides extracted with DMSO from the SWCNT-peptide suspension before (left) and after (right) oxidation. From top to bottom: SWCNT-Arg, SWCNT-Glu, SWCNT-Lys, SWCNT-Cys, SWCNT-Gly.

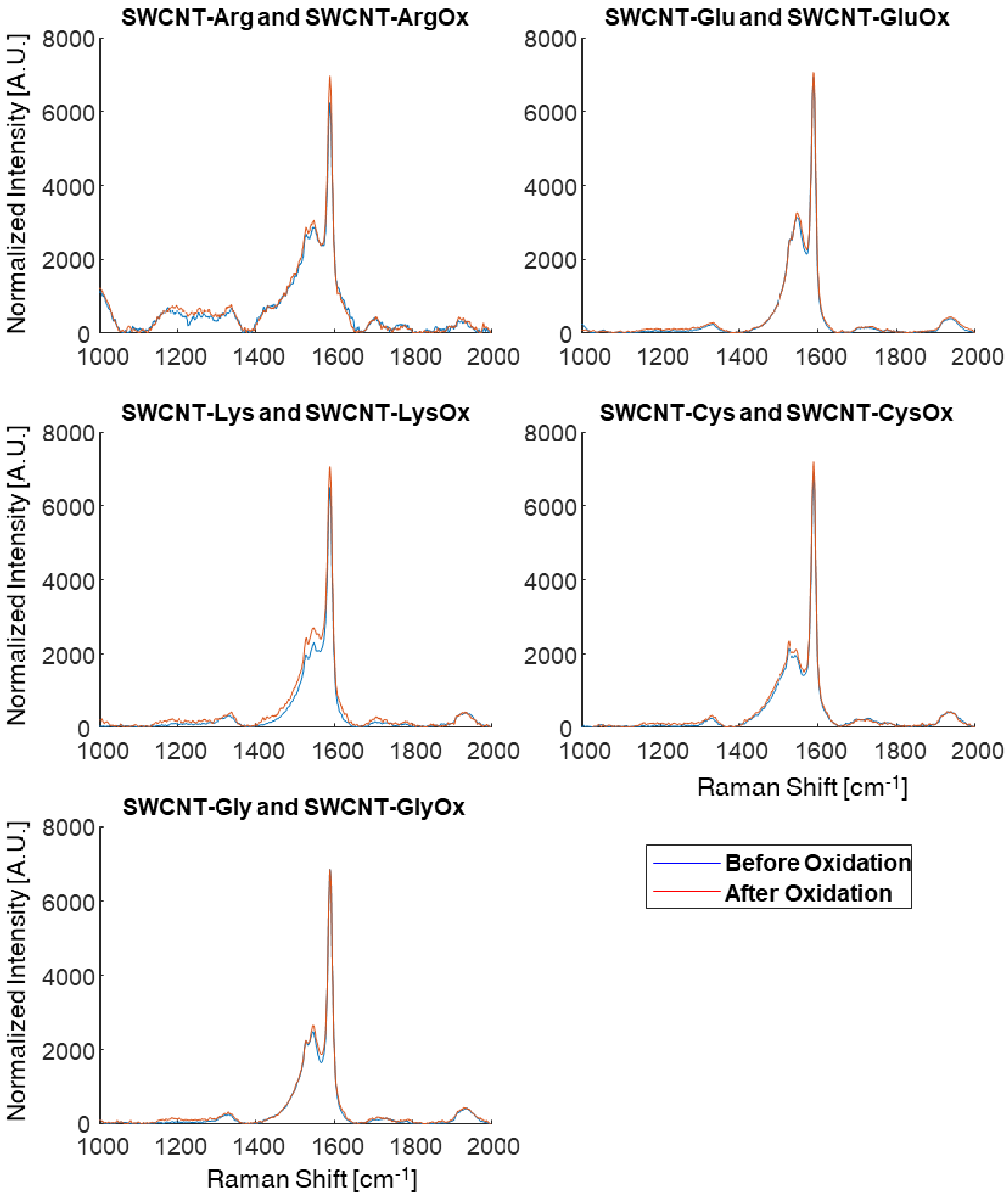

Figure S6: Raman spectra of the SWCNTs-peptides, before (blue) and after (red) oxidation, showing no significant changes suggesting conservation of the SWCNT lattice after UV exposure.

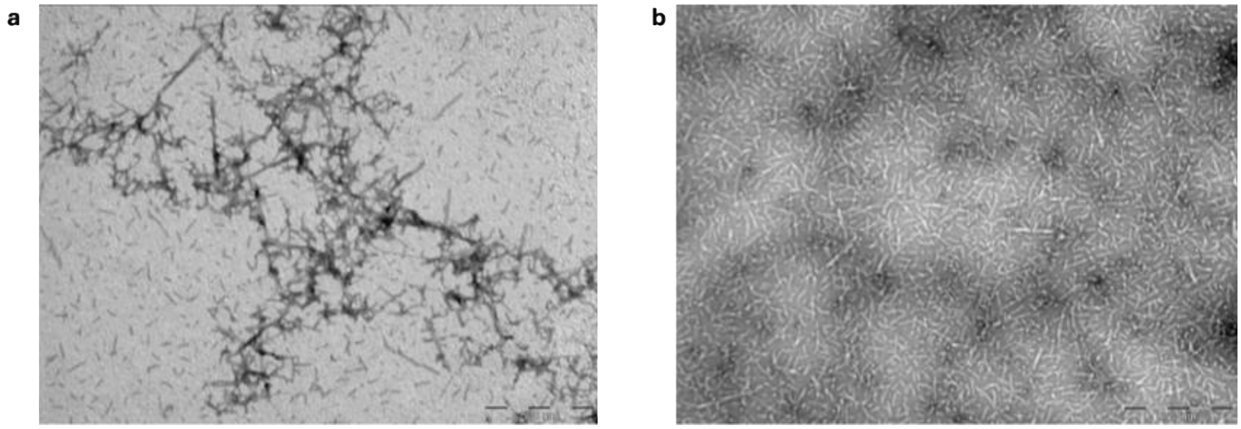

Figure S7: TEM of SWCNT-Lys. a) SWCNT-Lys suspension, with diameters ranging between 5 – 8 nm, and b) SWCNT-LysOx suspension following oxidization, with diameters ranging between 10 – 12 nm. Scale bar is 500 nm.

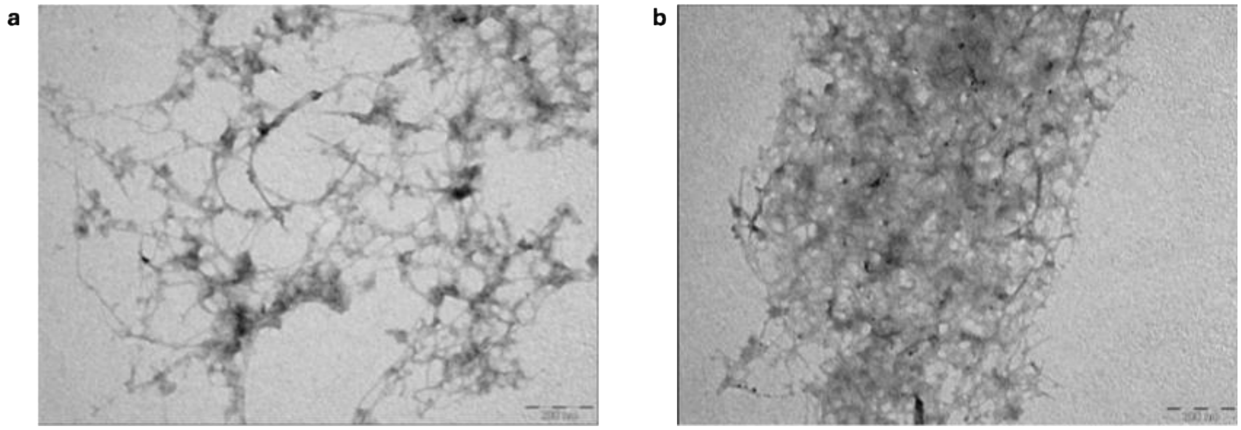

Figure S8: TEM of SWCNT-Cys. a) SWCNT-Cys suspension, with diameters ranging between 5 – 8 nm, and b) SWCNT-CysOx suspension following oxidization, with diameters ranging between 5 – 8 nm. Scale bar is 200 nm.

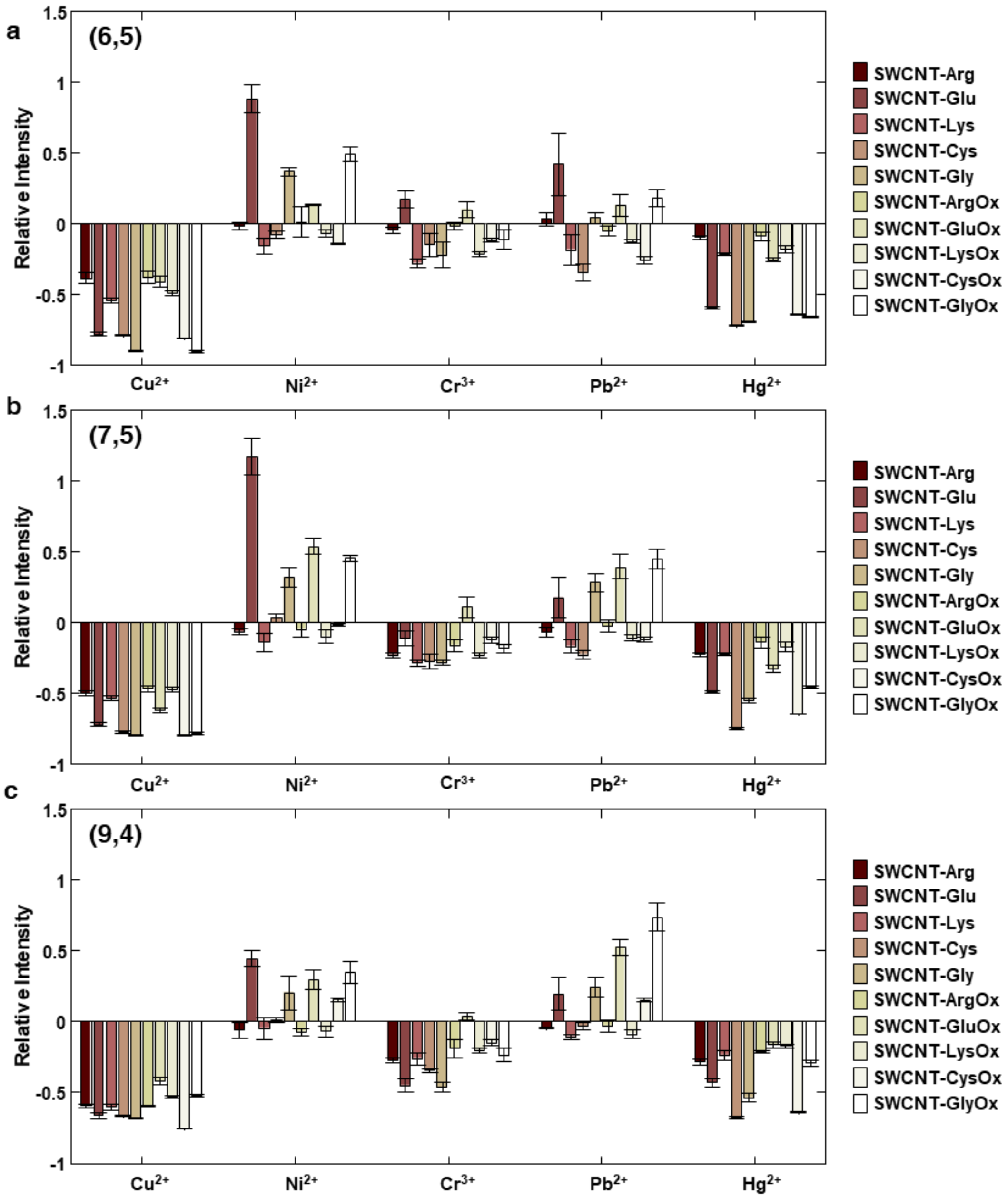

Figure S9: Relative fluorescence response of the SWCNT-peptide sensors to each metal at 300 μM for three different chiralities. a) (6,5), b) (7,5), and c) (9,4), excited at 570 nm, 660 nm, and 730 nm, respectively, resulting in 30 sensors in total. N=5.

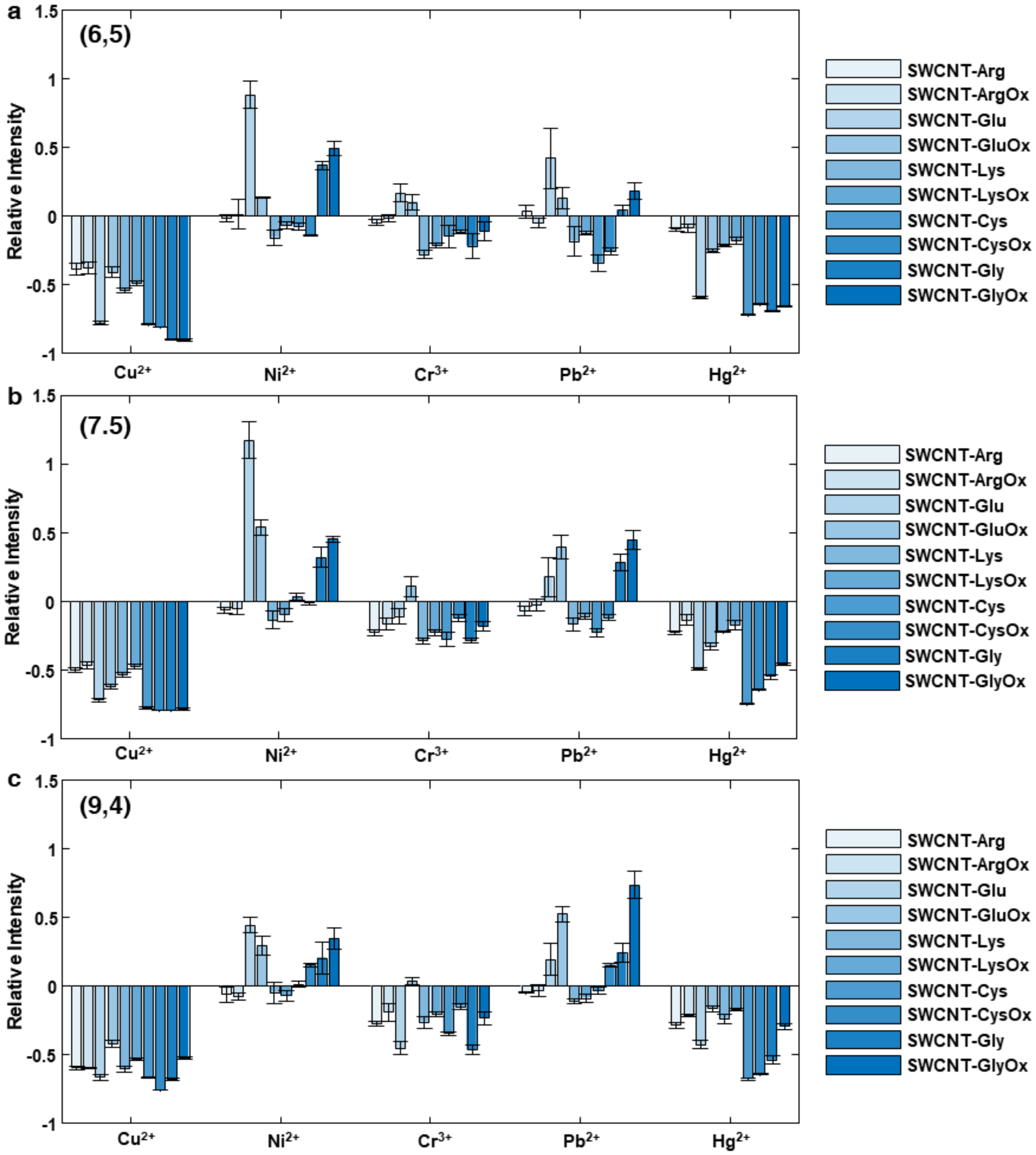

Figure S10: Relative fluorescence response of the SWCNT-peptide sensors to each metal at 300 μM for three different chiralities with native and oxidized sensors appearing next to each other. a) (6,5), b) (7,5), and c) (9,4), excited at 570 nm, 660 nm, and 730 nm, respectively. N=5.

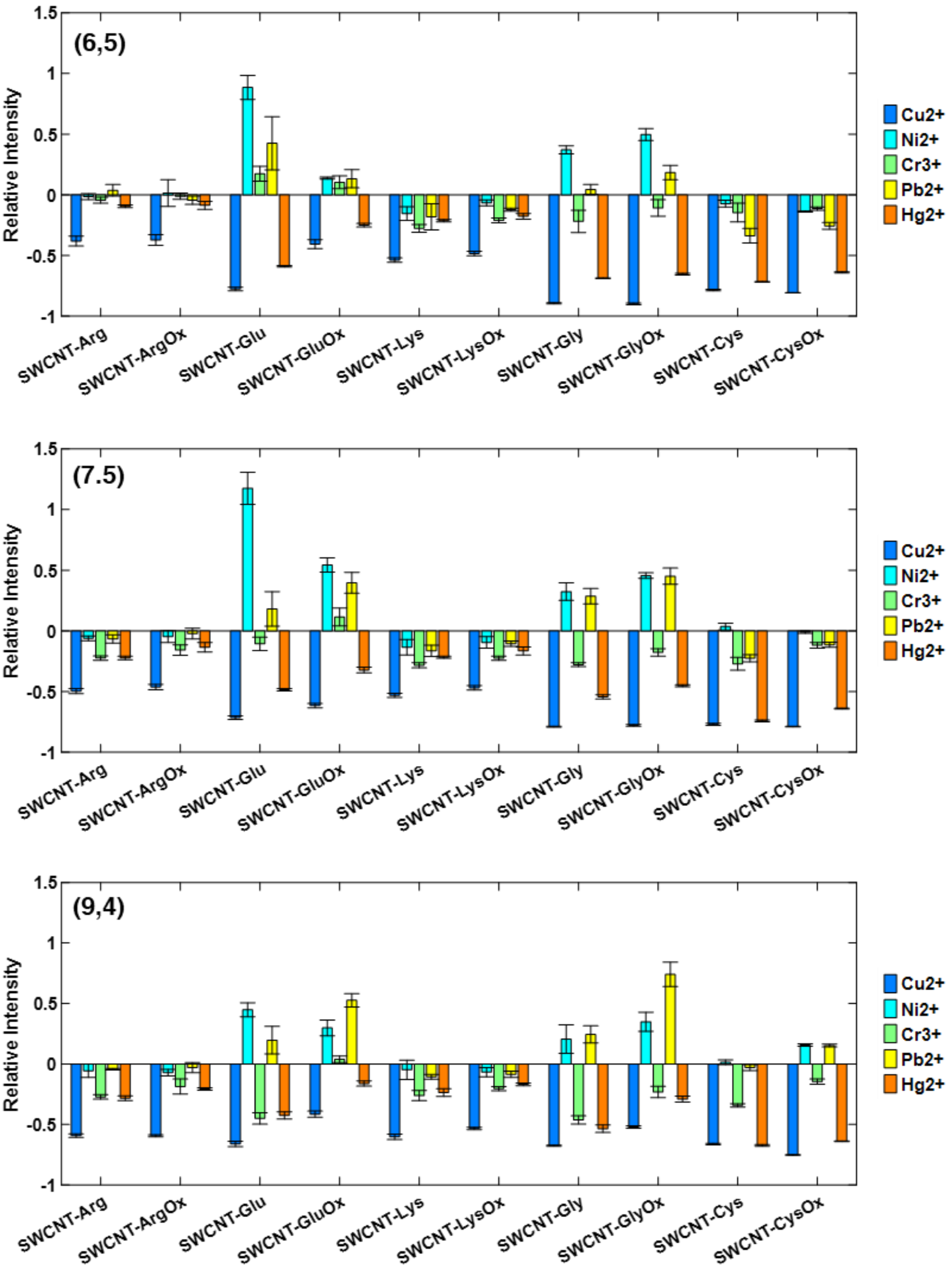

Figure S11: Relative fluorescence response of the SWCNT-peptide sensors to each metal at 300 μM for three different chiralities, a) (6,5), b) (7,5), and c) (9,4), excited at 570 nm, 660 nm, and 730 nm, respectively. N=5.

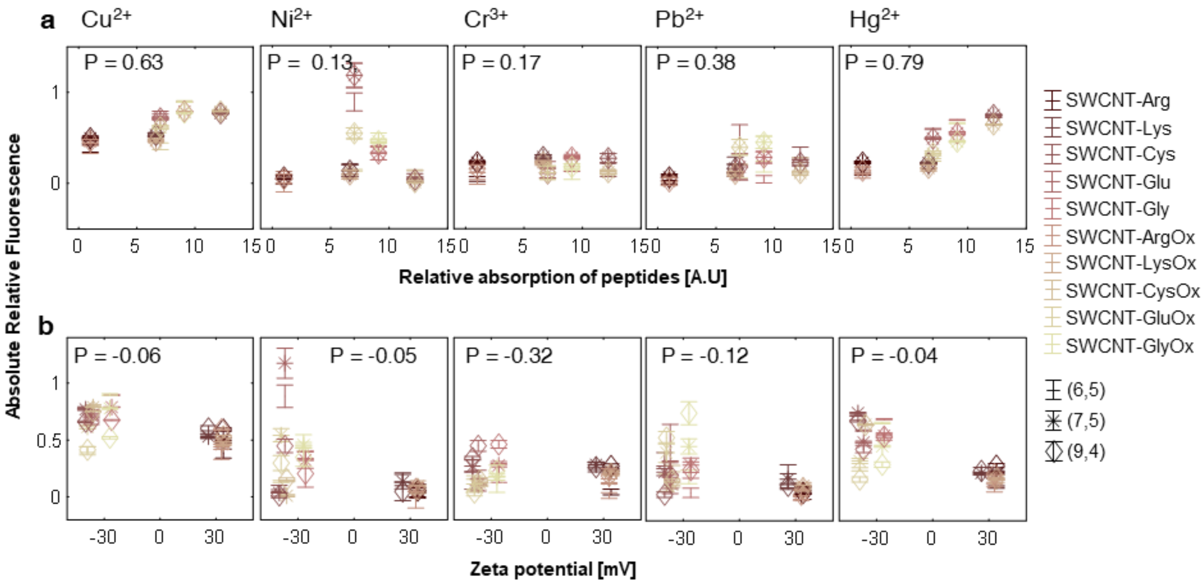

Figure S12: Correlation of the absolute relative fluorescence response of the SWCNT-peptide sensors to the peptide surface coverage or the zeta potential. a) Absolute relative fluorescence change and the Pearson correlation coefficient, P, of the 30 sensors upon adding metal-ions vs. the absorption of the Fmoc-group at 270 nm in the suspension, indicative of the peptide load on the SWCNT surface. N=3. b) Absolute relative fluorescence change and the Pearson correlation coefficient, *p*, of the 30 SWCNT-peptide sensors upon adding metal-ions vs. the zeta potential of the SWCNTs. N=3.

Section III: The Analyte Classification and Feature Selection Algorithm (ACFSA)

Introduction

We present a data-based scheme, namely, the Analyte Classification and Feature Selection (ACFSA), aiming to correctly classify a specific unknown metal analyte among those experimentally considered. We use this scheme to reduce the number of sensors required for the classification task, using a backward feature selection algorithm (BFSA).[1–3] Conducting classification-feature selection (FS) iterations aims to allow for careful and efficient planning of the required experimental effort to achieve high classification accuracy, without unnecessary redundancy.

The suggested method is unsupervised and data-based, ensuring high classification accuracy for analytes with concentrations matching those measured in the experiments. Nevertheless, as opposed to data-intensive classification and feature selection beneficial algorithms,[1–3] high classification accuracy is achieved by the ACFSA using solely five repetitions per sample. This renders the method invaluable for data-limited experiments.

We emphasize that the suggested classification scheme and the BFSA included in the ACFSA follow conventional heuristics to achieve valuable classification and feature selection capabilities. The ACFSA is general and thus can be beneficial as a combined wrapper and filter-response method for classification-aimed feature selection.[4,5]

1. The ACFSA Flowchart

The input data are taken from the experimental results presented in the main text, mainly the relative change in fluorescence intensity of each sensor when exposed to a metal-ion. The proposed scheme iteratively combines clustering and feature selection until a specified number of sensors, or a desired classification accuracy is reached. First, the input data is transformed using principal component analysis (PCA),[6] reducing the data dimensionality to 2. Second, clustering is performed on the PCA data using the *k*-means algorithm,[7,8] relying on prior knowledge of the number of clusters. Third, the cluster labels are matched and compared with the ground-truth data using the Kuhn-Munkres algorithm,[9,10] in a similar cluster comparison fashion as described in the work of

Faran et al.[11] Fourth, the clustering performance is evaluated against ground-truth data using the adjusted Rand index (ARI)[12] and is kept in the system memory. In the general case, this step is optional and depends on whether ground-truth clustering labels exist for the sake of comparison. In this work, it is presumed such ground-truth data are available. Fifth, a classifier is constructed based on the clusters, utilizing the cluster centers to create a nearest neighbor Voronoi tessellation.[13] Sixth, if the desired number of sensors has not yet been reached, the algorithm proceeds to feature elimination, removing one feature per iteration based on Chi-squared feature ranking.[14] Then, the previous steps are iterated with the remaining features as input data. In the final iteration, which depends on a stopping condition, such as the number of sensors or desired accuracy, a final classifier is constructed based on the selected features, once again utilizing the updated cluster centers to create a nearest neighbor Voronoi tessellation. Finally, the classification performance is calculated as a function of the number of sensors, which can be used to choose a working point by an experimentalist. Scheme S1 describes the flowchart of the suggested method, including all its different counterparts, and a detailed description of each of the AFSCA steps, and its implementation on the experimental data is provided afterward.

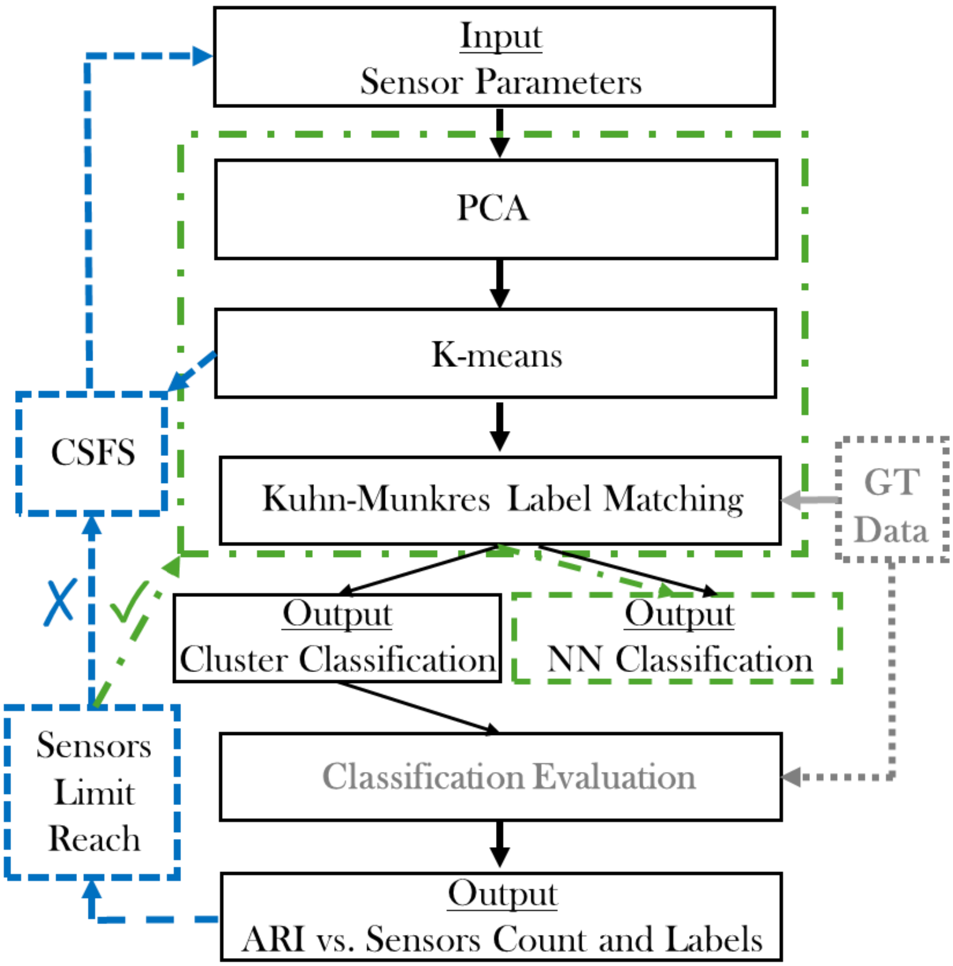

Scheme S1: Flowchart of the ACFSA process. Black boxes represent the core clustering steps, and blue boxes depict the backward feature selection stages. The final classifier steps are enclosed by a green dashed line, and their output is shown in a separate dashed green box. Optional steps are depicted with a dotted line and grey color. PCA: Principal component analysis, GT: Ground truth, ARI: Adjusted Rand index, NN Classification: Nearest neighbor Classification, CSFS: Chi-squared feature selection.

1.1. Input

For every analyte, the measurements of the sensor responses are randomly concatenated, one from each sensor type, to create the sensor measurement vectors. Each measurement vector is labeled as its analyte and kept as ground truth (GT) in the memory. Additionally, a stopping condition can be defined. For example, a minimal number of sensors or required accuracy conditions are optional user inputs that may terminate the iteration scheme, instead of the default

choice described. Unless stated otherwise, it is presumed by default that the minimal sensor number for the scheme stopping condition is one, which is the minimal sensor number overall in the AFSCA scheme. Additionally, no minimal accuracy condition is given.

The steps described below assume that the remaining data dimension, after feature reduction, is greater than 2. For the particular case where only one sensor remains, see the 1D ACFSA section afterward. Altering these conditions into a higher number of sensors or a coarser desired accuracy may improve the computational effort required by the ACFSA, as it could possibly terminate the iterative procedure earlier.

1.2. The Clustering Algorithm

PCA: The sensors' measurement vectors are transformed using principal component analysis.[6] The two main principal components (PC) are considered for subsequent analysis, and the other PC data are discarded. At this point, it is recommended to examine the latent values of PC1 and PC2, normalized by the latent values of all the principal components (PCs). If, indeed, the explained variability by the first two PCs is close to 1, eliminating other PCs is justified. If not, the current AFSCA methodology might be adjusted by considering a higher dimensional PC space as input to the next step.

***K*-means:** Using prior knowledge of K clusters (number of analytes), a *k*-means clustering[7,8] is conducted on the data in the 2D PC space. *k*-means aims to divide a set of n observations into K clusters. Each observation is assigned to the cluster whose mean is closest to it. Since there is an inherent randomness in the *k*-means algorithm that depends on a starting point, *k*-means is repeated ten times. Among the different repetitions, the clustering solution with the minimal within-cluster sum of point-to-centroid distances is selected. This results in class labels to the data.

1.3. The Classification Evaluation

Kuhn-Munkres label matching: To assess the *k*-means clustering success, label matching between the *k*-means labels and the GT labels (*the analytes*) is conducted using the Hungarian algorithm, like the label matching presented in Faran et. al.[11] Once the labels are matched, the *k*-means cluster labels are altered correspondingly. The new labels are referred to as the Kuhn-Munkres labels.

ARI calculation: The problem in hand is a multiclass classification task, and hence its classification merit is measured by the Adjusted Rand Index (ARI).[8] This metric measures the accuracy of determining whether a link belongs within a cluster or not, measured versus the ground truth, and a score between 0 and 1 is provided. ARI values close to 1 indicate immaculate accuracy corresponding to the ground truth, and 0 on the contrary.

Clustering output: The calculated ARI index, along with the current number of features and their labels, are extracted and kept in memory as output. Nevertheless, the labels and ARI information do not propagate backward in Scheme S1 and hence do not affect the feature subsequent feature selection process.

Intercluster distance: Each *k*-means cluster average distance from one another, $\langle D \rangle$, is calculated. Then, all the different cluster average distances are averaged again over all the clusters. The result is kept in memory and is abbreviated as the intercluster distance.

The clustering visualization figure: The data are scattered in the PC1 and PC2 space. Based on their ground-truth analyte label, they attain a different marker and different color. For the sake of data representation, 95% confidence eclipses are calculated for each *k*-means cluster, based on its Kuhn-Munkres label. This eclipse is calculated and drawn based on a Gaussian distribution assumption of the clusters, with main axes computed by performing another PCA within the same cluster data.[15] The eclipse colors follow the label ground-truth data color, depicted before. This data visualization enables a 2D intuitive clustering success measure, where the original ground truth is marked points, and the eclipses are the clustering scheme result.

1.4. Nearest Neighbor Classifier

Voronoi diagram construction: Concerning the data in memory, each cluster transformed measurements data are averaged in both the PC axes, yielding a 2D value per cluster. Using these values as cluster centers and assigning them their corresponding Kuhn-Munkres labels, a first nearest neighbor (Voronoi) diagram is constructed. This divides the 2D PC space into decision zones with labels corresponding to the original data labels,[13] which are to be used to classify new data drawn from the same distribution.

Classifier error calculation: The classifier estimated error for each analyte class can be calculated by assuming the experimental data follows Gaussian distributions, with means and

variances derived from the experiment. Since PCA is a linear transformation, it preserves the Gaussian nature of the data in the PC1 and PC2 space. Each *k*-means data cluster is represented by its 2D Gaussian mean in the PC space, and the variances are taken from the PCA uncertainty ellipses along their main axes. The classification error is estimated by assuming equal prior probabilities for the new data being from any of the five analytes' Gaussians. The error is calculated by numerically integrating the overlap of each Gaussian with the classifier Voronoi tiles other than its original tile. The average of these integrals results over five analytes gives the overall classification error.

1.5. Backward Feature Selection Algorithm

Sensor or Accuracy Limit Condition: If the minimal sensor number condition or required accuracy is met, the ACFSA is stopped.

Chi-squared Feature Selection: In the first iteration of the ACFSA, all the original input data are considered as features. Otherwise, the features are the remaining original sensor data that were not previously eliminated by the previous iterations. A chi-squared test follows, considering the *k*-means labels as the response variable, and the remaining feature data as the predictors.[14] The features are ranked based on their Chi-squared test scores. The lowest ranked feature data is then discarded, and the remaining features are considered as input data for the ACFSA subsequent iterations.

1.6. Nearest Neighbor Final Classifier

Voronoi diagram construction: Once the ACFSA stopping condition is met, the last iteration PCA transformation matrix, the transformed measurements data, the remaining features (sensors), and the Kuhn-Munkres cluster labels are used to construct the final Voronoi classifier diagram. With the stopping condition being one remaining sensor, see below the ACFSA scheme is activated on the data. The classifier error is calculated as described before.

Using the classifier: Assuming a new sensor data measurement is conducted, the sensors selected as remaining features are solely considered (*the classifier input)*. The examined analyte is unknown. Using the ACFSA result, one seeks to identify the solution analyte. For a 2D final classifier, the data is transformed using the last iteration PCA transformation matrix. This yields a single 2D PC values pair. Based on the Voronoi diagram, the value pair falls within a certain zone

and attains the zone label. This label is the predicted analyte of the new measurement *(the classifier output)*. For 1D, the measured values are assigned a zone label based on the raw data, as PCA is not required for one dimension.

1.7. 1D ACFSA

If only one sensor remains after feature selection by the ACFSA, the final iteration of the scheme is initiated. The PCA step in Scheme S1 is skipped. *K*-means clustering and Kuhn-Munkres label matching are performed with more sensors, but 2D data visualization is skipped. The ARI index and intercluster distances are calculated in 1D. Final classifier decision points are set midway between cluster means, with the leftmost and rightmost clusters assigned values of -20 and 20, respectively. These values are significantly distant from the leftmost and rightmost cluster means, correspondingly, compared to any other two neighboring clusters (theoretically, they should be minus plus infinity). Each zone, defined by adjacent decision points, receives a Kuhn-Munkres label based on the *k*-means clustering matching with ground truth, yielding the final classifier. The estimated error is calculated based on 1D overlap.

1.8. Working Point Selection

Using the resulting ARI and classifier error versus iteration number, the ACFSA working point selection figure is constructed. Since the ACFSA is a single feature backward feature selection algorithm (BFSA) elimination scheme, the remaining iteration number corresponds to the number of sensors used as features. Therefore, the x-axis is labeled as the number of sensors. This figure illustrates the classification accuracy achieved with varying numbers of sensors, allowing experimentalists to select the optimal number of sensors to achieve their desired accuracy, thereby minimizing experimental effort.

2. Time Complexity Analysis

The time complexity of the ACFSA is detailed below, excluding the Voronoi classifier creation, as it is optional for the final classifier interest. Let $\boldsymbol{n}$ be the number of features, $\boldsymbol{m}$ the number of samples per feature, $\boldsymbol{k}$ the number of clusters, and $\boldsymbol{t}$ the iterations for *k*-means convergence. Despite decreasing feature count per iteration, the complexity remains dominated by the quadratic sum of an arithmetic progression in $\boldsymbol{n}$.

Combining these phases, the overall complexity is:

$O(n^3 \cdot m + n^4)$ (PCA phase over n iterations)

+ $O(k \cdot m \cdot t \cdot n)$ (k-means clustering over n iterations)

+ $O(n \cdot k^3 \cdot m)$ (Kuhn-Munkres algorithm over n iterations)

+ $O(n \cdot m^2)$ (ARI calculation over n iterations)

+ $O(n^2 \cdot m)$ (Chi-squared feature selection over n iterations)

Dominant Term Analysis:

Case 1: $n, m \gg k, t, n$ **and** m **are of similar order of magnitude**

When n and m are much larger than k and t, and n and m are of similar order of magnitude, the complexity is dominated by the PCA phase. The overall complexity is $O(n^4 + n^3 \cdot m)$.

Case 2: $n \gg m, k, t$

When n is much larger than m, k, and t, the complexity remains dominated by the PCA phase. The overall complexity is $O(n^4 + n^3 \cdot m)$.

Case 3: $m \gg n, k, t$

When m is much larger than n, k, and t, the complexity is dominated by the ARI calculation. The overall complexity is $O(n \cdot m^2 + n \cdot k^3 \cdot m)$.

Conclusion:

The algorithm's complexity is heavily influenced by the number of features (n), primarily due to the PCA operation performed at each iteration. This dependency renders the method time-consuming for datasets with a large number of features. Future work should focus on optimizing the algorithm’s time complexity to enhance its applicability to larger datasets.

3. ACFSA Implementation with Simulated Data

We created an artificial dataset by fitting a Gaussian distribution to the original input sensor data for each analyte. Then, the standard deviation of the respective Gaussians was multiplied by a factor of four, and fifty samples were withdrawn from these distributions as a new dataset. The latter was conducted to examine the ACFSA potential result for real-world scenarios where unknown samples may be analyzed and for data with greater variability and additional measurement repetitions (Figure S13). The ARI accuracy remained high at around 90% across all sensors, demonstrating the robustness of our classification approach. The apparent partial overlap between the clusters of nickel (Ni^{2+}) and lead (Pb^{2+}) reduces the ARI compared to the real experimental data (Figure S13a). Nevertheless, five repetition measurements and an averaged measurement standard deviation of approximately 3% for a screening experiment is a common experimental scenario; hence, the 100% ARI for all the sensors obtained for the original dataset is reasonable.

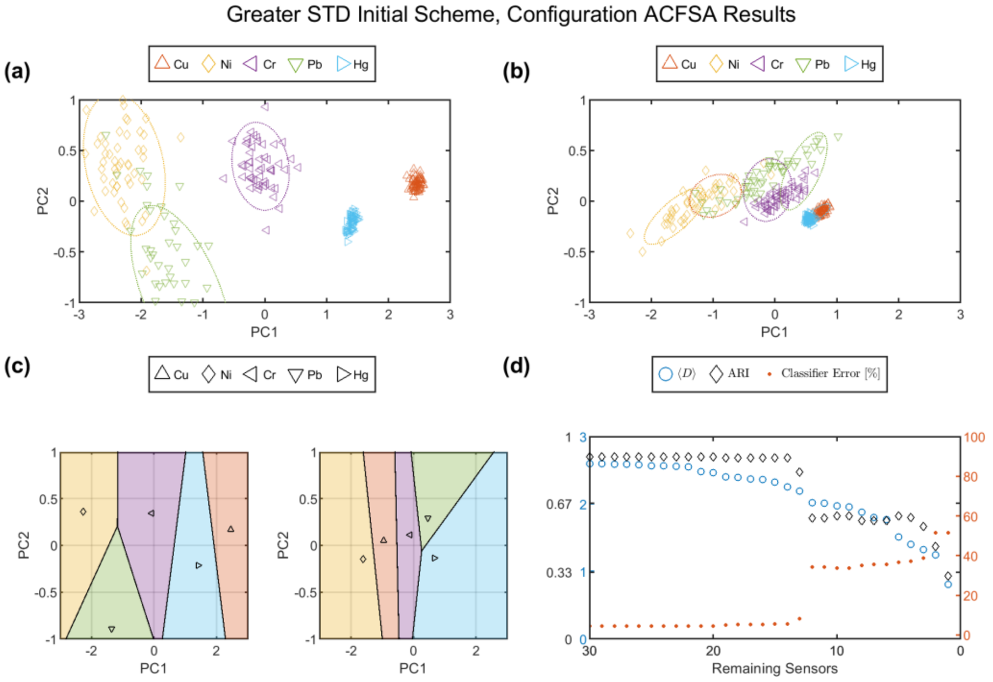

Figure S13: The ACFSA result for all the SWCNT-peptide sensor data for the artificial dataset, containing more samples and a standard deviation (STD) increased fourfold for each cluster. 2D principal component representations of the data are depicted in a) and b), for all the sensor data and two remaining sensors, respectively. PC1 and PC2 each explain 70%, 6%, and 94%, 5.5% of

the data variability for the two configurations, respectively. The markers and colors of the analyte data are described in the legend. The data are compared versus their 95% uncertainty ellipses, depicted in dashed lines with similar colors to their enclosed analyte data, which represent the *k*-means result. c) The Voronoi classifier is presented in the 2D principal components space for the first scheme iteration (left) and for the remaining two sensors' iteration (right). Each tile center matches the respective analyte as in the legend, and the tile colors follow the analyte colors of a) and b). The average intercluster distance (blue circles), the adjusted Rand index (black diamond, comparing the ground truth data and clustering labels match), and the Voronoi classifier error (orange dots) are shown in d) versus the scheme iteration number. Each point data was created given a stopping condition of a required minimal number of a single sensor.

4. ACFSA with Randomized Feature Selection

Furthermore, a randomized feature selection scheme was examined, instead of the Chi-squared method in the ACFSA (Figures S14 and S15). This test examines the Chi-squared single feature elimination performance compared with a randomized feature selection reference. The randomized selection resulted in six required sensors for an ARI of 1 (100%), while the Chi-squared employed by the algorithm resulted in only two sensors. This is reasonable since selecting sensors based on a rational statistical test is expected to yield better results than a randomized process, which further strengthens the ACFSA.

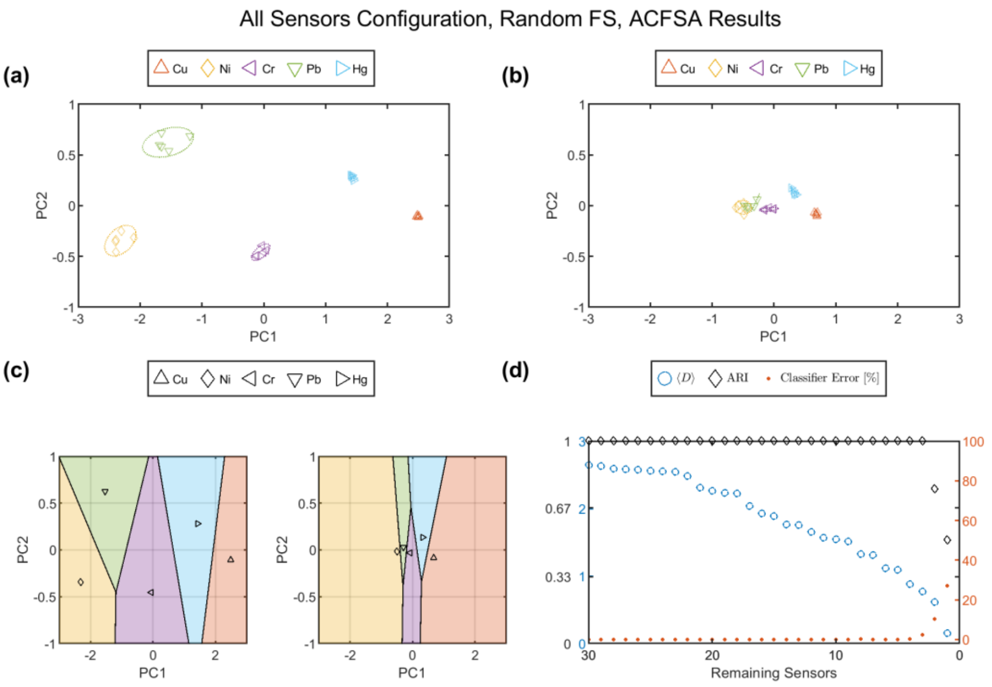

Figure S14: The ACFSA result for all the SWCNT-peptide sensors, with a random feature elimination scheme, instead of the Chi-squared method. 2D principal component representations of the data are depicted in a) and b), for all the sensor data and two remaining sensors, respectively. PC1 and PC2 each explain 89%, 4.5%, and 95%, 4% of the data variability for the two configurations, respectively. The markers and colors of the analyte data are described in the legend. The data are compared versus their 95% uncertainty ellipses, depicted in dashed lines with similar colors to their enclosed analyte data, which represent the *k*-means result. c) The Voronoi classifier is presented in the 2D principal components space for the first scheme iteration (left) and for the remaining two sensors' iteration (right). Each tile center matches the respective analyte as in the legend, and the tile colors follow the analyte colors of a) and b). The average intercluster distance (blue circles), the adjusted Rand index (black diamond, comparing the ground truth data and clustering labels match), and the Voronoi classifier error (orange dots) are shown in d) versus the scheme remaining iteration number. Each point data was created given a stopping condition of a required minimal number of a single sensor.

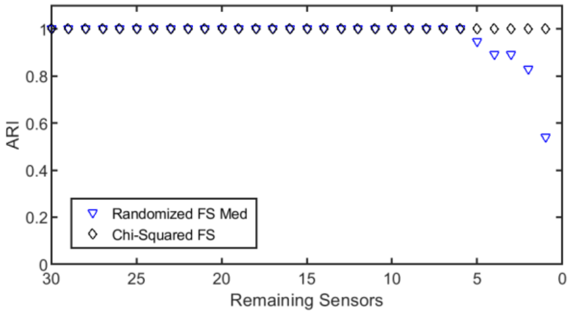

Figure S15: ARI versus the remaining iteration number for all sensors included configuration. The original ACFSA result (with Chi-squared feature selection) is depicted using black diamonds, while the median ARI of the randomized feature selection over 10 repetitions is shown with blue triangles.

5. ACFSA Implementation with Experimental Data

For all the original dataset configurations, five samples were considered for each sensor, and the number of clusters considered was also five, reflecting the desired number of analytes to be detected. The measurements' vectors were constructed, resulting in five vectors for the five analyte classes, in the size of the total sensor number considered. The optimal sensor sets for different input data subsets of SWCNT-peptides selected by the ACFSA are shown in Table S1.

Table S1: ACFSA-selected sensor sets for different input data subsets of SWCNT-peptides.

Library	# (Selected sensors)	Sensors selected[a]	Classification error
All SWCNT-peptides	2	SWCNT-Gly-(6,5)<br>SWCNT-GlyOx-(6,5)	0.02%
non-oxidized SWCNT-peptides	2	SWCNT-Cys-(6,5)<br>SWCNT-Gly-(6,5)	0.19%
oxidized SWCNT-peptides	9	SWCNT-ArgOx-(6,5)<br>SWCNT-GluOx-(6,5)<br>SWCNT-CysOx-(6,5)<br>SWCNT-GlyOx-(6,5)<br>SWCNT-GluOx-(7,5)<br>SWCNT-CysOx-(7,5)<br>SWCNT-GlyOx-(7,5)<br>SWCNT-ArgOx-(9,4)<br>SWCNT-GluOx-(9,4)	0.35%
(6,5)-chirality SWCNT-peptides	2	SWCNT-Cys-(6,5)<br>SWCNT-Gly-(6,5)	0.19%
(9,4)-chirality SWCNT-peptides	2	SWCNT-Glu-(9,4)<br>SWCNT-Gly-(9,4)	0.04%
(7,5)-chirality SWCNT-peptides	4	SWCNT-GluOx-(7,5)<br>SWCNT-CysOx-(7,5)<br>SWCNT-GlyOx-(7,5)<br>SWCNT-Glu-(7,5)	0.3%

[a]Sensors are listed in the order of their survival if the elimination were to continue beyond the stopping condition. The first sensor listed would be the last to remain.

We observe an inconsistency in the sensor elimination by comparing the remaining sensor set of the (6,5)-SWCNT-peptides with the result of all 30 SWCNT-peptides. With the input data of all 30 SWCNT-peptide sensors, the ACFSA chose SWCNT-Gly-(6,5) and SWCNT-GlyOx-(6,5) as the surviving sensors with an ARI of 1 (100%) and a classification error of 0.02%, both of which belong to the subsets of only (6,5) chirality SWCNT-peptides. However, the algorithm chooses a sensor set of two different sensors, SWCNT-Gly-(6,5) and SWCNT-Cys-(6,5), with a classification error of 0.19% (Table S1). This discrepancy stems from the iterative Chi-squared feature selection, where different initial sensor sets lead to varying elimination sequences and classification outputs. Consequently, the ACFSA may converge to different outcomes depending on the initial starting conditions, highlighting the importance of a comprehensive screening dataset to ensure robust results.

In the following Figures, we show the ACFSA results for the different data subsets for comparison. Figure S16 includes all the SWCNT-peptide sensors. Figures S17 and S18 consider only the oxidized and non-oxidized sensors as input, respectively. Finally, Figures S19, S20, and S21 correspond to the ACFSA results for the (6,5), (7,5), and (9,4) SWCNT-peptide chiralities, respectively.

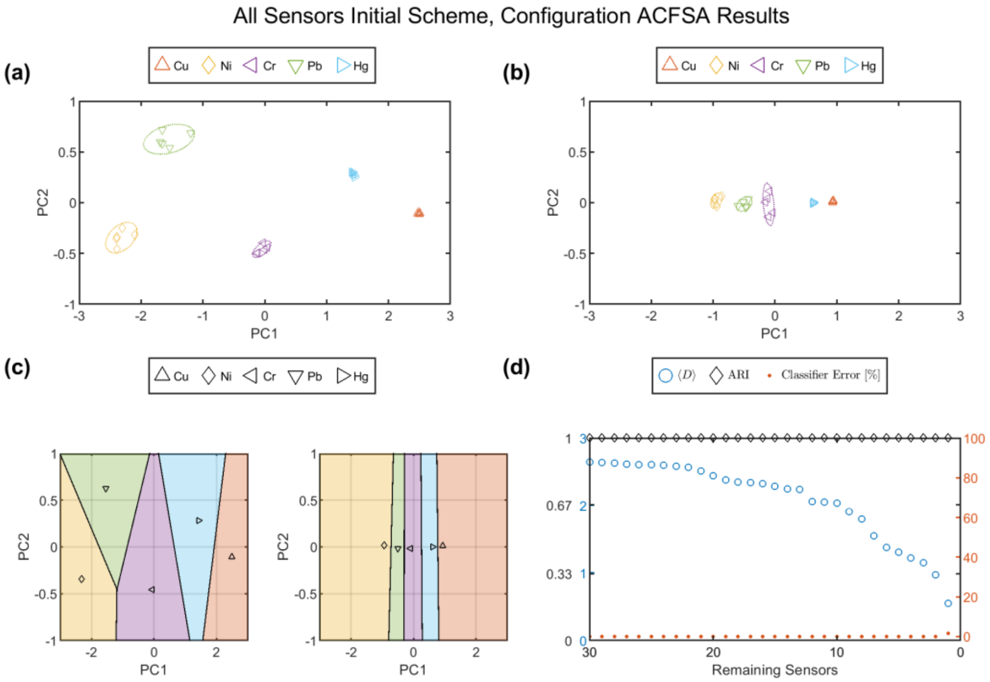

Figure S16: The ACFSA results for all the SWCNT-peptide sensors. 2D principal component representations of the data are depicted in a) and b), for all the sensor data and two remaining sensors, respectively. PC1 and PC2 each explain 89%, 4.5%, and 99.5%, 0.5% of the data variability for the two configurations, respectively. The markers and colors of the analyte data are described in the legend. The data are compared versus their 95% uncertainty ellipses, depicted in dashed lines with similar colors to their enclosed analyte data, which represent the *k*-means result. c) The Voronoi classifier is presented in the 2D principal components space for the first scheme iteration (left) and for the remaining two sensors' iteration (right). Each tile center matches the respective analyte as in the legend, and the tile colors follow the analyte colors of a) and b). The average intercluster distance (blue circles), the adjusted Rand index (black diamond, comparing the ground truth data and clustering labels match), and the Voronoi classifier error (orange dots) are shown in d) versus the scheme iteration number. Each point data was created given a stopping condition of a required minimal number of a single sensor.

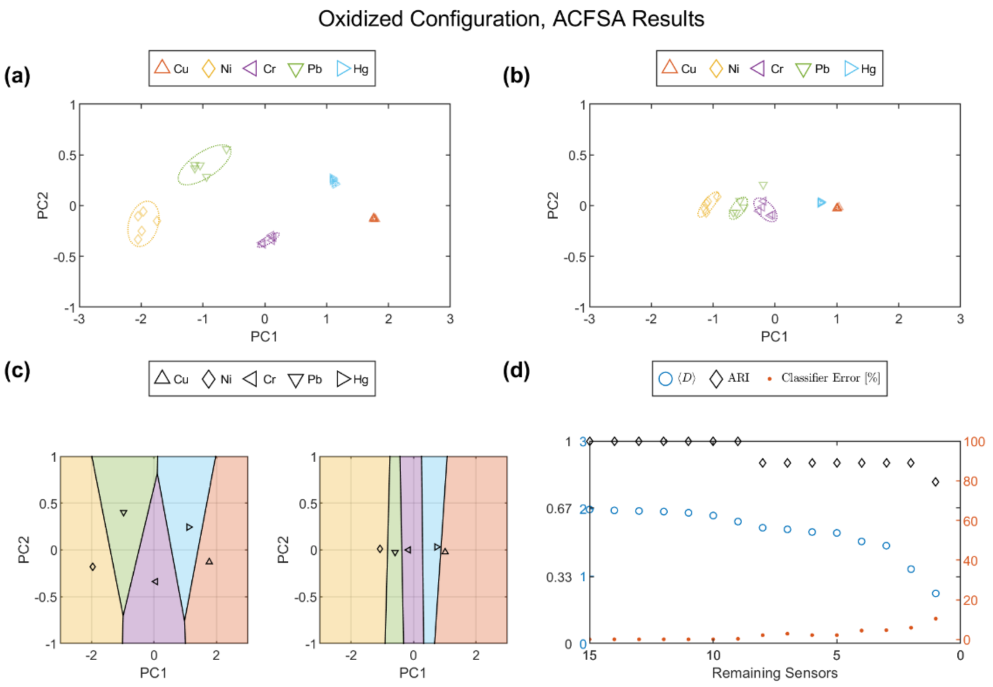

Figure S17: The ACFSA results for the oxidized SWCNT-peptide sensors. 2D principal component representations of the data are depicted in a) and b), for all the sensor data and two remaining sensors, respectively. PC1 and PC2 each explain 90%, 5%, and 99.3%, 0.6% of the data variability for the two configurations, respectively. The markers and colors of the analyte data are described in the legend. The data are compared versus their 95% uncertainty ellipses, depicted in dashed lines with similar colors to their enclosed analyte data, which represent the *k*-means result. c) The Voronoi classifier is presented in the 2D principal components space for the first scheme iteration (left) and for the remaining two sensors' iteration (right). Each tile center matches the respective analyte as in the legend, and the tile colors follow the analyte colors of a) and b). The average intercluster distance (blue circles), the adjusted Rand index (black diamond, comparing the ground truth data and clustering labels match), and the Voronoi classifier error (orange dots) are shown in d) versus the scheme iteration number. Each point data was created given a stopping condition of a required minimal number of a single sensor.

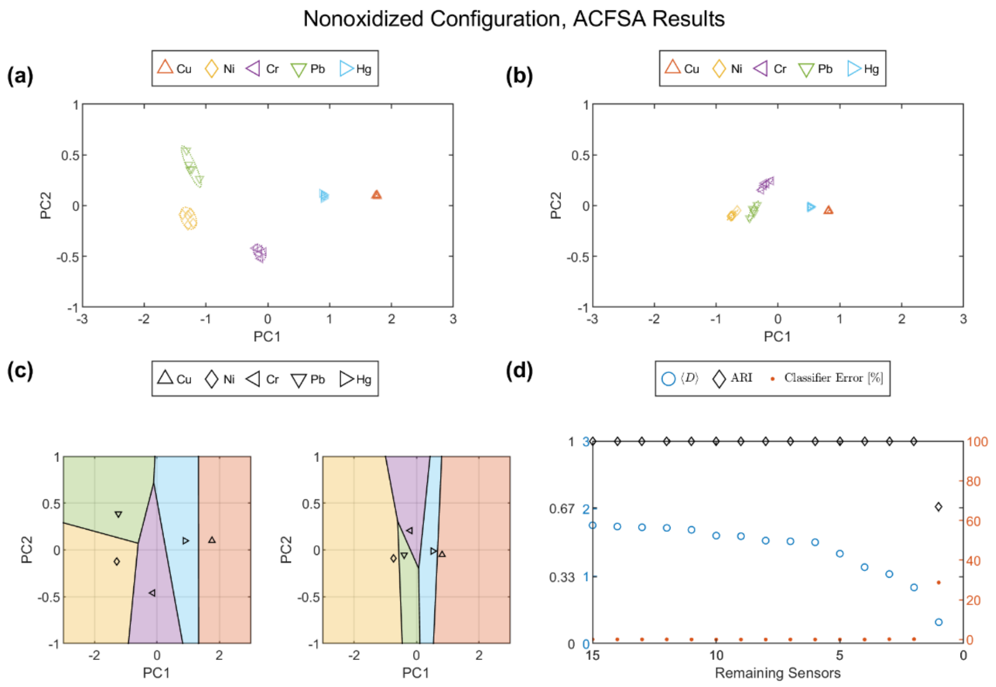

Figure S18: The ACFSA results for the non-oxidized SWCNT-peptide sensors. 2D principal component representations of the data are depicted in a) and b), for all the sensor data and two remaining sensors, respectively. PC1 and PC2 each explain 90%, 4%, and 96%, 3% of the data variability for the two configurations, respectively. The markers and colors of the analyte data are described in the legend. The data are compared versus their 95% uncertainty ellipses, depicted in dashed lines with similar colors to their enclosed analyte data, which represent the *k*-means result. c) The Voronoi classifier is presented in the 2D principal components space for the first scheme iteration (left) and for the remaining two sensors' iteration (right). Each tile center matches the respective analyte as in the legend, and the tile colors follow the analyte colors of a) and b). The average intercluster distance (blue circles), the adjusted Rand index (black diamond, comparing the ground truth data and clustering labels match), and the Voronoi classifier error (orange dots) are shown in d) versus the scheme iteration number. Each point data was created given a stopping condition of a required minimal number of a single sensor.

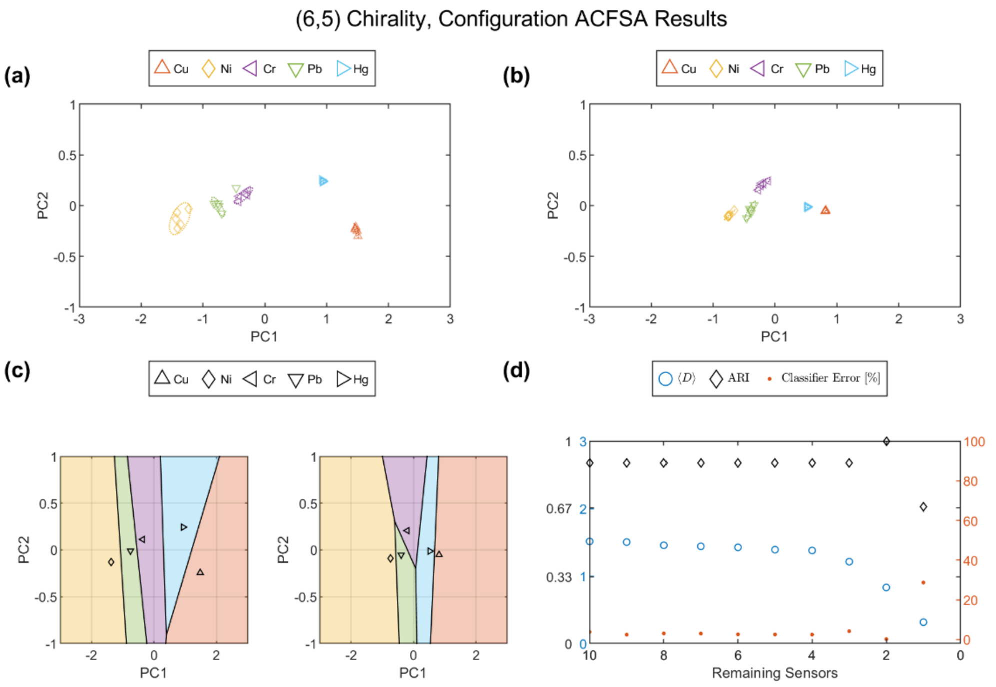

Figure S19: The ACFSA results for the (6,5) chirality SWCNT-peptide sensors. 2D principal component representations of the data are depicted in a) and b), for all the sensor data and two remaining sensors, respectively. PC1 and PC2 each explain 93%, 2.5%, and 96.5%, 3% of the data variability for the two configurations, respectively. The markers and colors of the analyte data are described in the legend. The data are compared versus their 95% uncertainty ellipses, depicted in dashed lines with similar colors to their enclosed analyte data, which represent the *k*-means result. c) The Voronoi classifier is presented in the 2D principal components space for the first scheme iteration (left) and for the remaining two sensors' iteration (right). Each tile center matches the respective analyte as in the legend, and the tile colors follow the analyte colors of a) and b). The average intercluster distance (blue circles), the adjusted Rand index (black diamond, comparing the ground truth data and clustering labels match), and the Voronoi classifier error (orange dots) are shown in d) versus the scheme iteration number. Each point data was created given a stopping condition of a required minimal number of a single sensor.

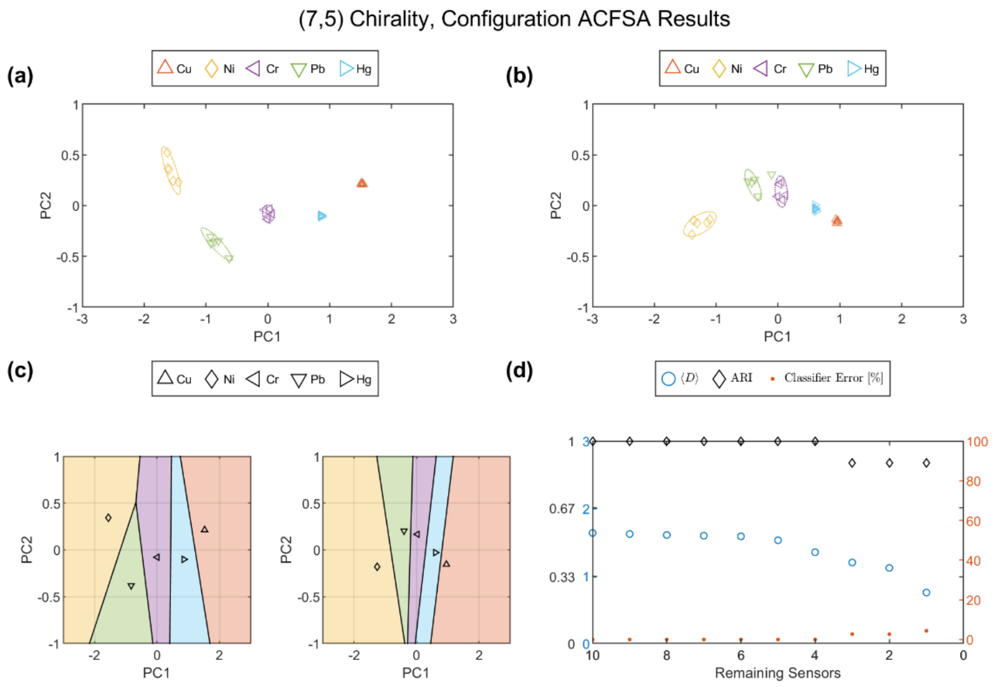

Figure S20: The ACFSA results for the (7,5) chirality SWCNT-peptide sensors. 2D principal component representations of the data are depicted in a) and b), for all the sensor data and two remaining sensors, respectively. PC1 and PC2 each explain 91%, 5%, and 95.5%, 4.5% of the data variability for the two configurations, respectively. The markers and colors of the analyte data are described in the legend. The data are compared versus their 95% uncertainty ellipses, depicted in dashed lines with similar colors to their enclosed analyte data, which represent the *k*-means result. c) The Voronoi classifier is presented in the 2D principal components space for the first scheme iteration (left) and for the remaining two sensors' iteration (right). Each tile center matches the respective analyte as in the legend, and the tile colors follow the analyte colors of a) and b). The average intercluster distance (blue circles), the adjusted Rand index (black diamond, comparing the ground truth data and clustering labels match), and the Voronoi classifier error (orange dots) are shown in d) versus the scheme iteration number. Each point data was created given a stopping condition of a required minimal number of a single sensor.

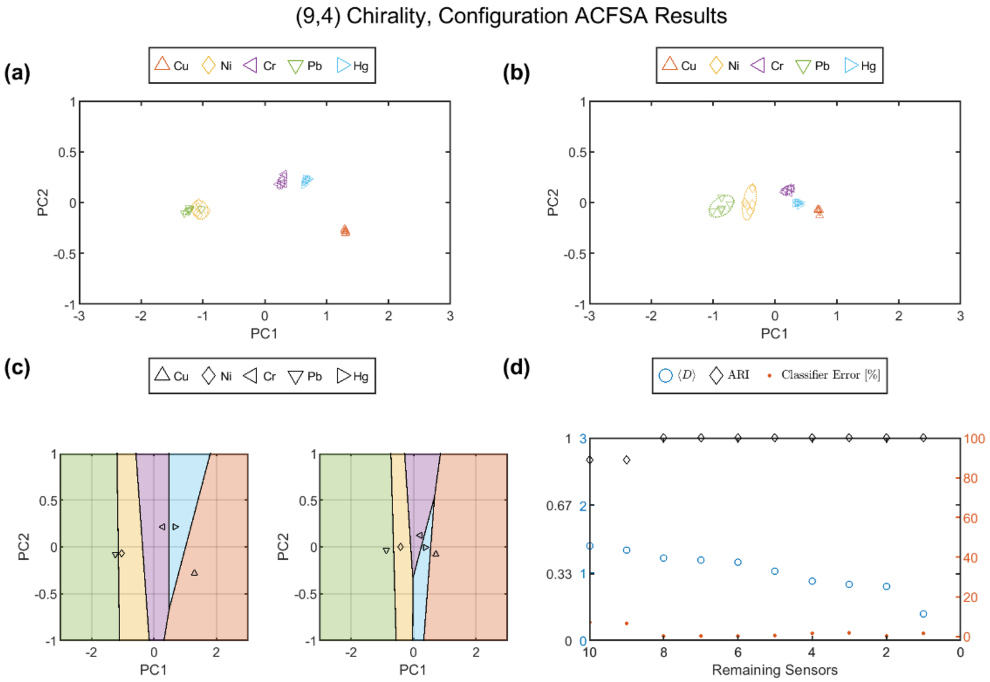

Figure S21: The ACFSA results for the (9,4) chirality SWCNT-peptide sensors. 2D principal component representations of the data are depicted in a) and b), for all the sensor data and two remaining sensors, respectively. PC1 and PC2 each explain 90.5%, 3.5%, and 98%, 2% of the data variability for the two configurations, respectively. The markers and colors of the analyte data are described in the legend. The data are compared versus their 95% uncertainty ellipses, depicted in dashed lines with similar colors to their enclosed analyte data, which represent the *k*-means result. c) The Voronoi classifier is presented in the 2D principal components space for the first scheme iteration (left) and for the remaining two sensors' iteration (right). Each tile center matches the respective analyte as in the legend, and the tile colors follow the analyte colors of a) and b). The average intercluster distance (blue circles), the adjusted Rand index (black diamond, comparing the ground truth data and clustering labels match), and the Voronoi classifier error (orange dots) are shown in d) versus the scheme iteration number. Each point data was created given a stopping condition of a required minimal number of a single sensor.

6. Pearson correlation coefficients for the intercluster distance with the classifier error, and for the ARI with the classifier error

Apparently, the ARI and intercluster distance $\langle \boldsymbol{D} \rangle$ decrease with a decreasing number of sensors, and the classification error increases across almost all configurations (Figure 4 and Figures S16-S21). An exception is the (9,4) Chirality configuration (Figure S21), where eliminating sensors has improved accuracy, which can result from some sensors adding more noise to the system. Table S2 presents the Pearson correlation coefficients between $\langle \boldsymbol{D} \rangle$ or ARI, and the classification error, generally indicating a negative correlation.

Table S2: Pearson correlation coefficients for the intercluster distance with the classifier error $\rho(\langle D \rangle, error)$, and for the ARI with the classifier error ($\rho(ARI, error)$). For all sensor configurations, the ARI is 1 for all the iteration numbers, so the Pearson coefficient is not defined.

**Configuration**	$\boldsymbol{\rho(\langle D \rangle, error)}$	$\boldsymbol{\rho(ARI, error)}$
All Sensors	-0.5	-
Oxidized	-0.98	-0.9
Non-Oxidized	-0.73	-1
(6,5) Chirality	-0.8	-0.94
(7,5) Chirality	-0.95	-0.96
(9,4) Chirality	0.45	-0.97
Artificial Dataset	-0.93	-0.99
Random Feature Selection	-0.58	-0.99

7. The ACFSA algorithm on a binary response set

After collecting and processing the original data with the ACFSA algorithm, we explored its applicability on a binary response set, where only the trend of the fluorescence change (either turn-on or turn-off) is considered. This approach is based on the assumption that the trend of the response is preserved across all concentrations within the sensor's dynamic range. In this binary framework, sensors that exhibited an increase in fluorescence in response to the analyte were assigned a value of 1, while those with a decrease in fluorescence intensity were assigned a value of -1. This allowed us to construct a new classification PCA (Figure S22a) that, being independent of intensity, effectively removes concentration dependency. The results from this binary PCA analysis, supported by the original bar plots, demonstrate that as long as measurements remain above the detection threshold, the method can individually classify nickel and partly classify chromium and lead due to some overlap. However, consistent negative responses exhibited by all sensors for copper and mercury inhibit differentiability between these two metals, but our approach can successfully separate them from the other analytes. This analysis underscores that even with single-concentration measurements, a concentration-independent fluorescence-response trend allows for some classification capability, suggesting that with additional measurements across various concentrations, achieving full classification of all analytes is a promising possibility.

To further evaluate the classification potential of the binary PCA approach, we analyzed the data distribution using a Gaussian fit and a Voronoi diagram. Specifically, we fitted Gaussian distributions to the principal components (PC1 vs. PC2) for nickel and lead, adding 95% confidence ellipses (Figure S22b). Additionally, we constructed a Voronoi diagram for the binary classification, using the mean PCA coordinates of each analyte as tile centers. This visualization highlights the classification boundaries and provides a geometric interpretation of the separation between analytes. To quantify classification performance, we estimated the classification error for nickel and lead based on the overlap between their corresponding Gaussian distributions and the Voronoi tiles. The resulting classification errors were 3% and 28%, respectively, reflecting the limited but nontrivial distinguishability between these two metals. Still, while the separation is imperfect, it remains informative and improves upon random guessing.

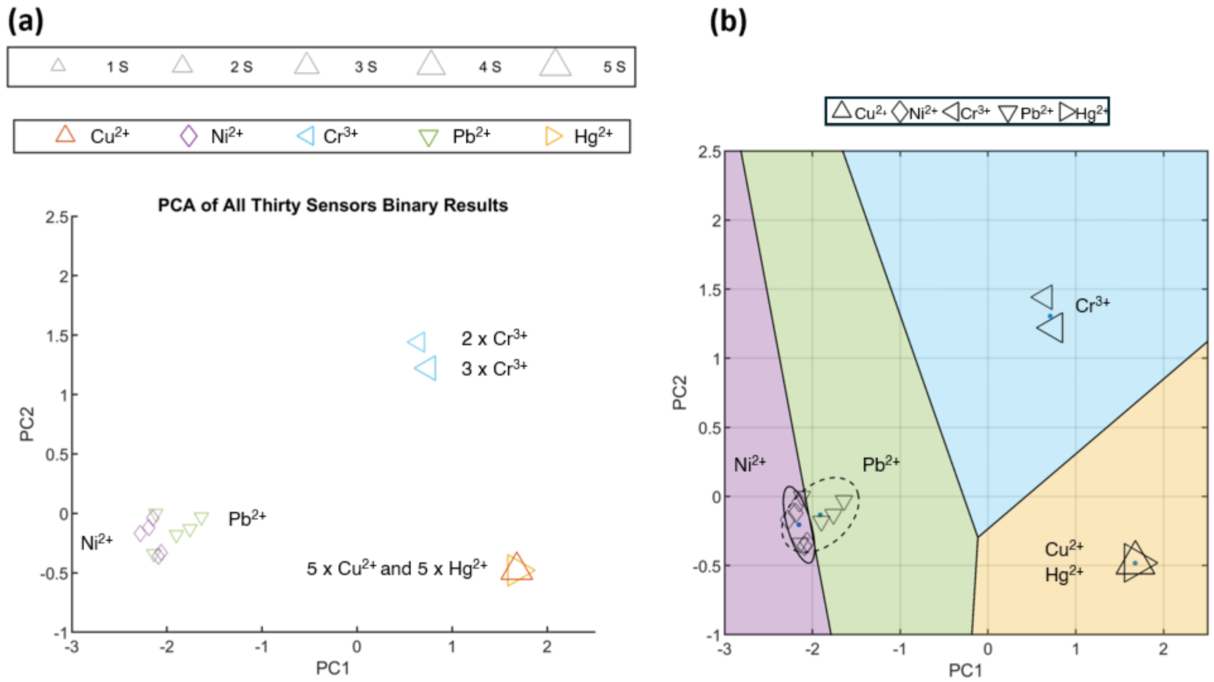

Figure S22: Binary principal component analysis and classifier. a) PCA of the 30 sensors based on binary fluorescence response trends (turn-on or turn-off). The color and shape of the data points represent different metal ions (legend in the second bar), while the size of the shapes corresponds to the number of overlapping data points (referenced by the gray triangle scale in the first bar). The number of data points and the metal-ions are listed next to the corresponding marker (For example, 2 x Cr^{3+} indicates two overlapping Cr^{3+} datapoints at the same PCA position). b) The Voronoi classifier in the 2D principal components space for all 30 sensors binary data, with the same color scheme, analyte shape, and size coding of the data in a). Blue points show the cluster centers that correspond to each tile. The 95% uncertainty ellipses of the clustering of Nickel and Lead are depicted in continuous and dashed black lines, respectively.

Section IV: Limit of detection and sensor response in Serum and Mineral Water

Limit of detection for SWCNT-Gly-(6,5)

To further explore the response of sensors to different concentrations and identify detection limits, we performed an analysis on SWCNT-Gly-(6,5), the sensor selected by the algorithm as the most effective for classification purposes. The concentration-dependent responses for all tested metal ions are shown in Figure S23, revealing detection limits of $3.0x10^{-9}$ M for copper, $7.2x10^{-5}$ M for chromium, $7.1x10^{-7}$ M for mercury, $8.1x10^{-6}$ M for lead, and $3.6x10^{-5}$ M for nickel. While these results indicate variability in detection limits for the classification of analytes, it is important to note that this analysis does not imply SWCNT-Gly is the most sensitive sensor for a specific analyte, but rather demonstrates its utility for classification tasks across varying concentrations. Importantly, these findings show that the fingerprint patterns are preserved for certain analytes even when concentration varies, highlighting the potential to extend this approach to larger datasets spanning a broader range of concentrations for classification and sensor selection.

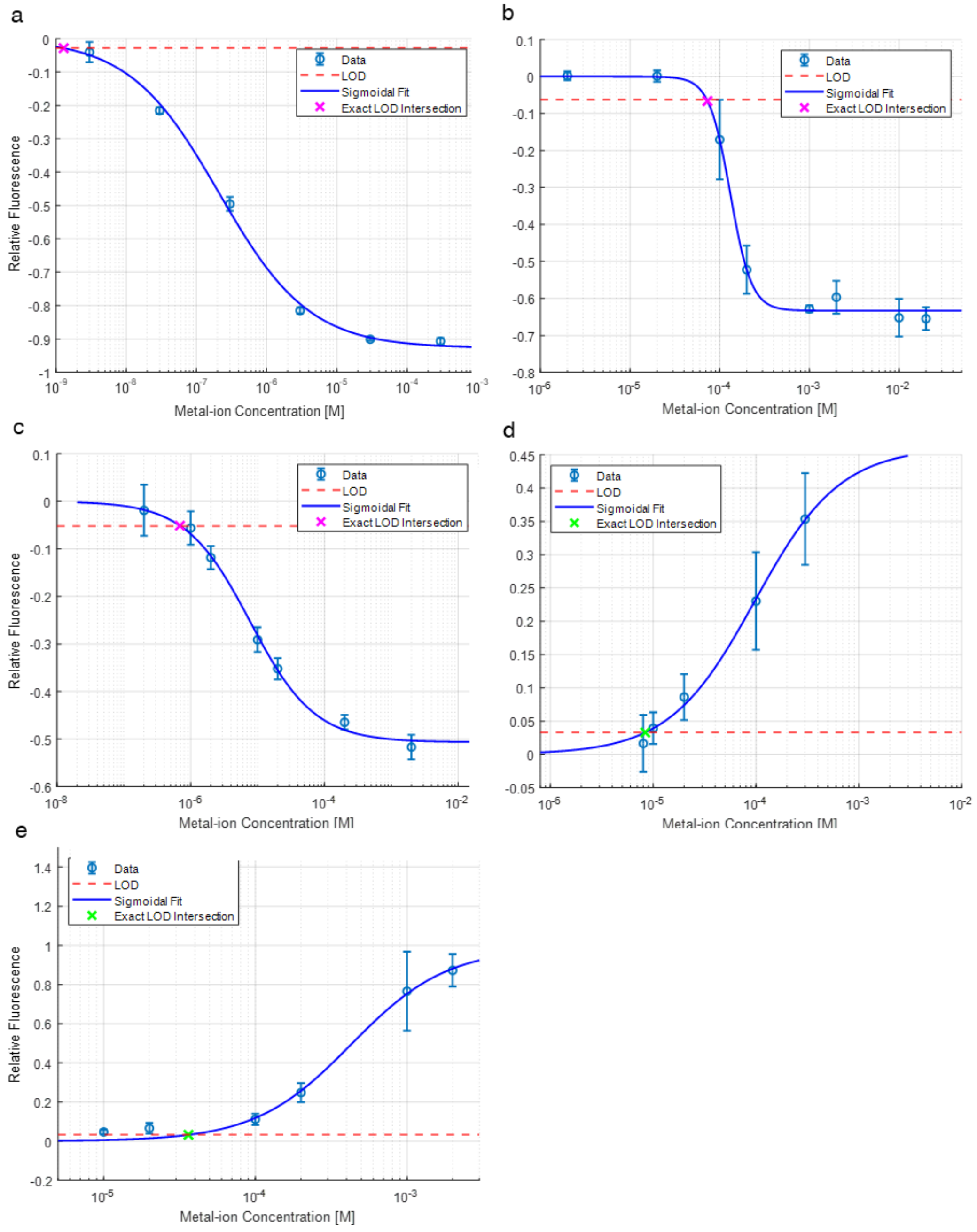

Figure S23: Relative fluorescent response of the sensor SWCNT-Gly-(6,5) vs. a range of metal-ion concentrations. Measured in triplicates. Data points with error bars and sigmoidal fit of the data (blue), 3 times the STD value of the sensor alone (dashed red) and, intersection marked with a green X. Limit of detection for a) Copper: $3.0x10^{-9}$ M, b) Chromium: $7.2x10^{-5}$ M, c) Mercury: $7.1x10^{-7}$ M, d) Lead: $8.1x10^{-6}$ M, and e) Nickel: $3.6x10^{-5}$ M.

Sensor performance in mineral water and serum

To assess the potential functionality of these sensors in different environments, additional experiments measuring the response of the SWCNT-Gly-(6,5) sensor to the analytes were conducted in serum and in commercial mineral water (Figure S24). Both environments produced distinct fingerprinting patterns, although they differed from those observed in water, probably due to the interaction of both the SWCNTs and metal-ions with other serum components or with the dissolved minerals or gases in mineral water. These results suggest that SWCNT-Gly, and potentially other sensors as well, could maintain their fingerprinting capabilities across diverse matrices. However, variations in the content and pH of each environment may necessitate adjustments to the peptide library and require the generation of new fingerprinting datasets to account for matrix-specific factors.

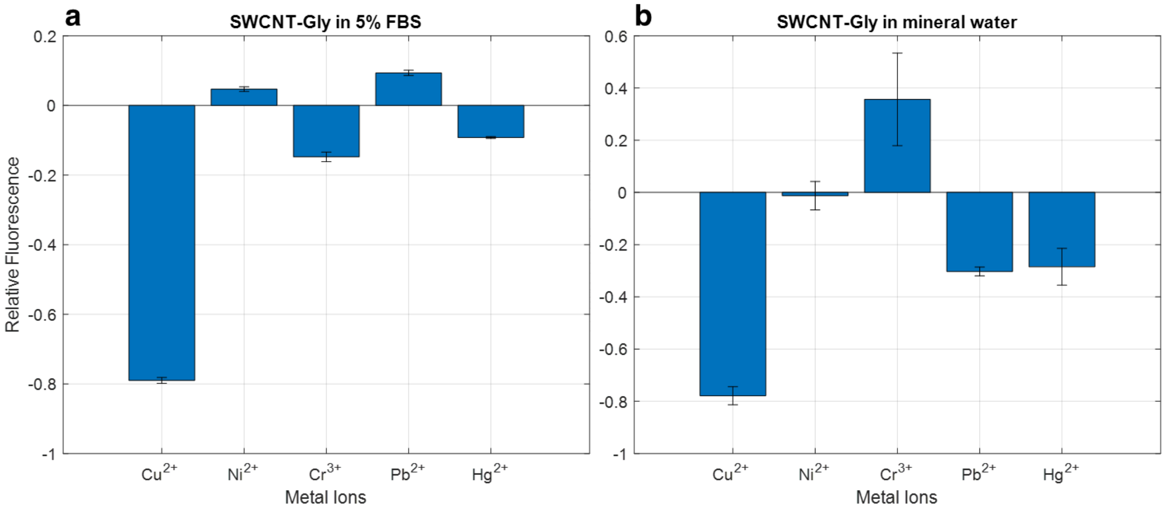

Figure S24: Relative fluorescence response of the sensor SWCNT-Gly-(6,5) to the five metal-ions. Cu^{2+}, Ni^{2+}, Cr^{3+}, Pb^{2+}, and Hg^{2+} at 300 μM, in a) fetal bovine serum and b) commercial mineral water. N=5.

Section V: Sensor stability and batch variations

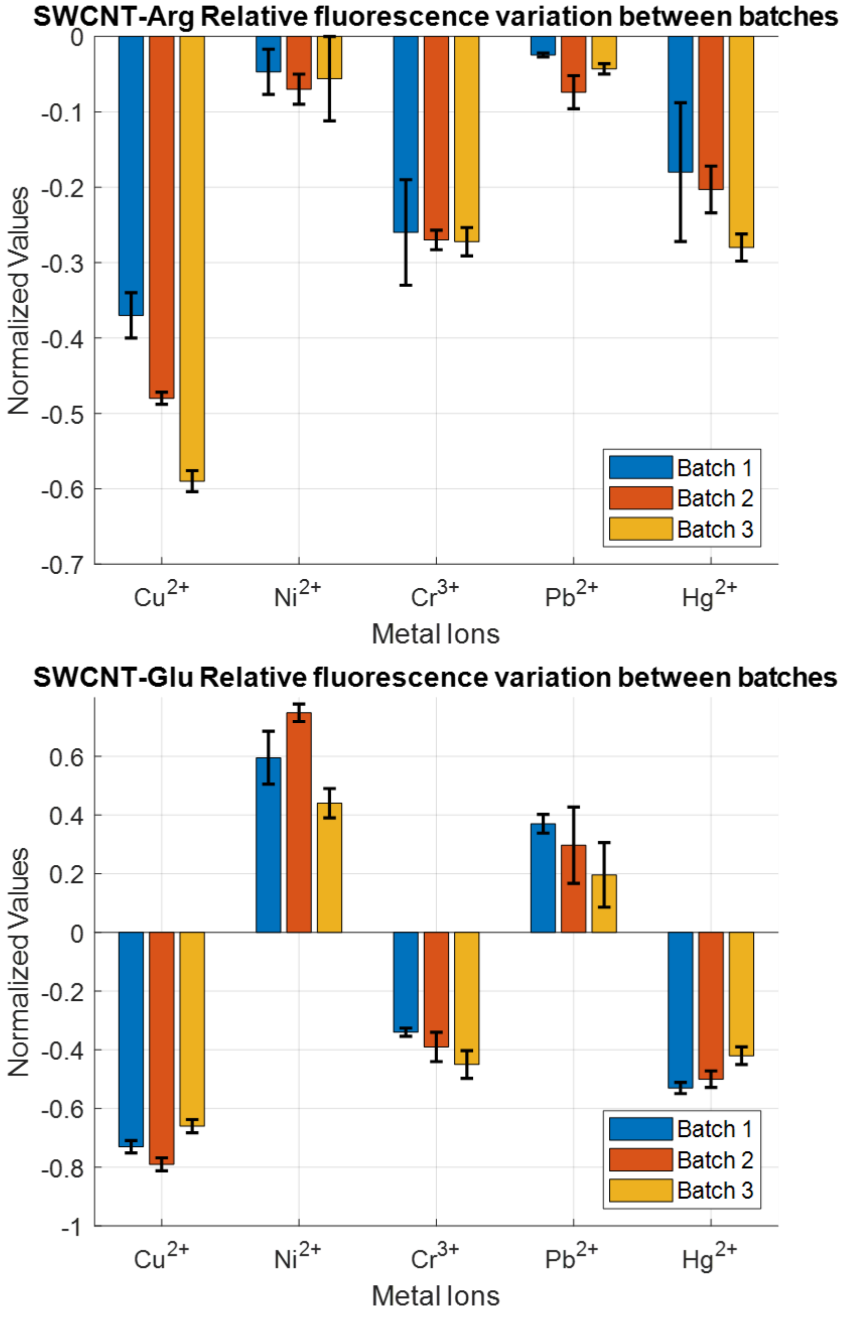

Figure S25: Batch-to-batch sensor variation. Normalized fluorescence values of SWCNT-Arg and SWCNT-Glu sensors across three independent batches for the five metal-ions, Cu^{2+}, Ni^{2+}, Cr^{3+}, Pb^{2+}, and Hg^{2+} at 300 µM. N=3.

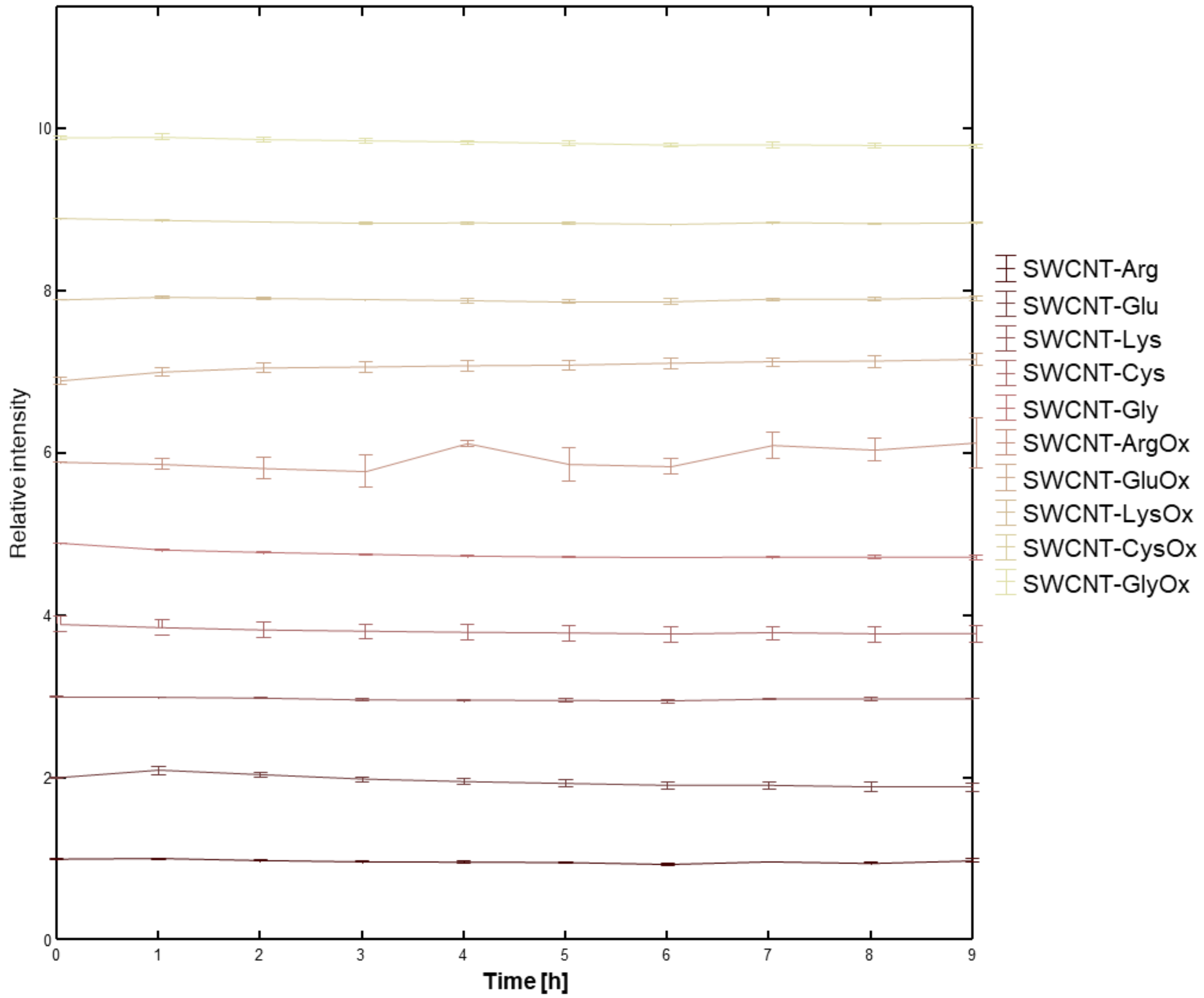

Figure S26: Short-term sensor stability. Relative fluorescence intensity of the 10 sensors, offset clarity. The sensors retain their fluorescence emission for 9 hours under continuous laser irradiation, with only minor drift observed in SWCNT-ArgOx after 3 hours. N=3.

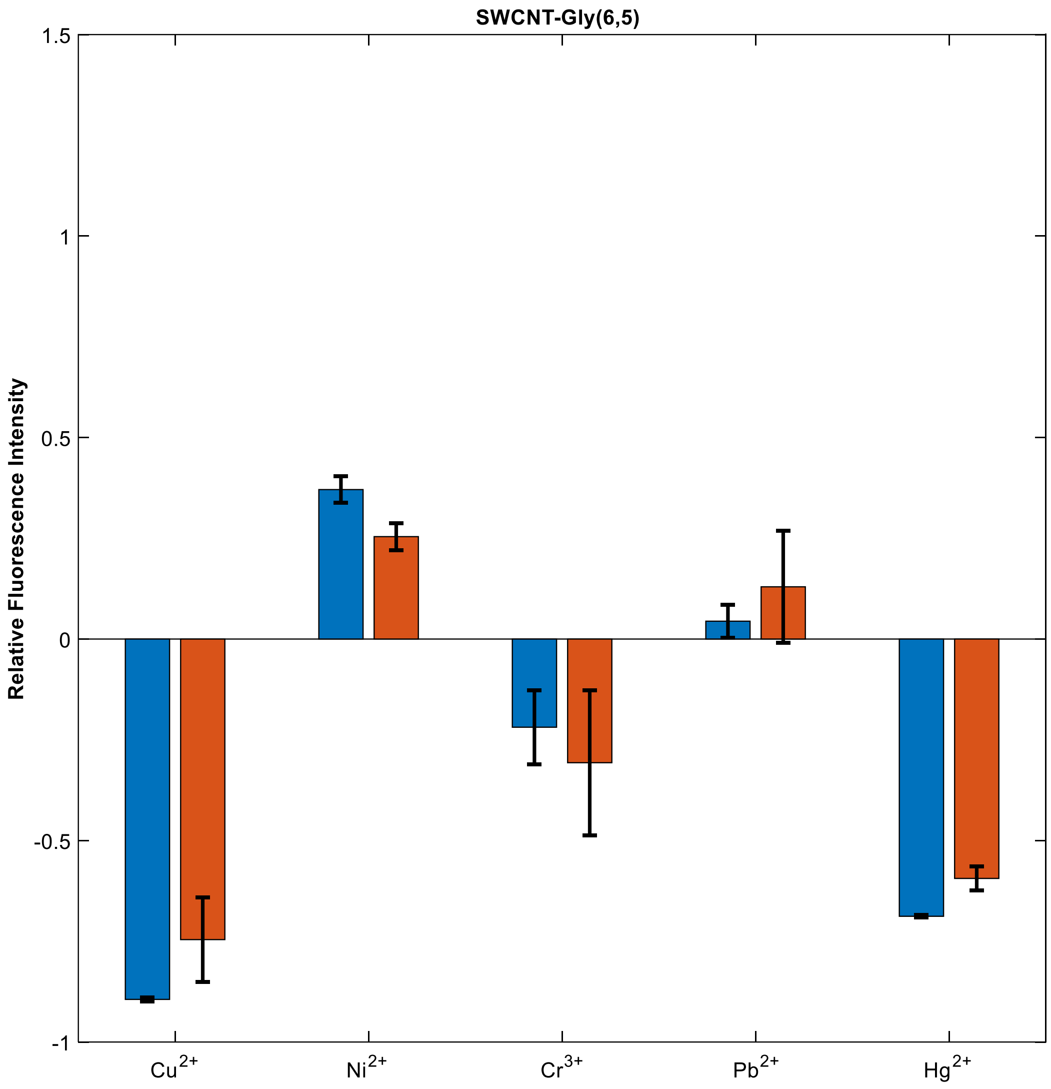

Figure S27: SWCNT-Gly response after 6 months. Relative fluorescence response of the same sensor SWCNT-Gly to the five metal-ions at 300 µM, before (blue) and after 6 months (orange). N=5.

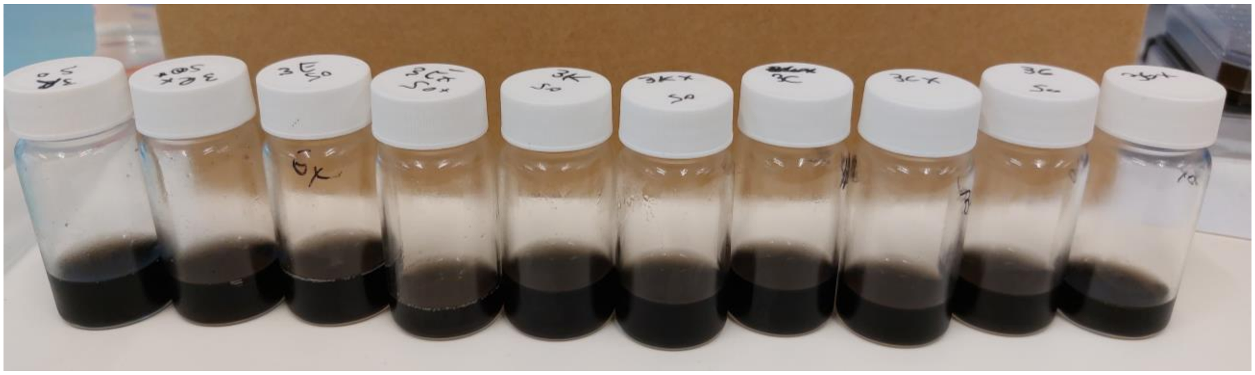

Figure S28: Suspension stability after 6 months. The Suspensions show no aggregates when stored at 50 mg L-1 and 4°C. From left to right: SWCNT-Arg, SWCNT-ArgOx, SWCNT-Glu, SWCNT-GluOx, SWCNT-Lys, SWCNT-LysOx, SWCNT-Cys, SWCNT-CysOx, SWCNT-Gly, SWCNT-GlyOx.

References

(1) Too, J.; Abdullah, A. R. A New and Fast Rival Genetic Algorithm for Feature Selection. *J. Supercomput.* **2021**, *77*, 2844–2874.

(2) Cai, J.; Luo, J.; Wang, S.; Yang, S. Feature Selection in Machine Learning: A New Perspective. *Neurocomputing* **2018**, *300*, 70–79.

(3) Chandrashekar, G.; Sahin, F. A Survey on Feature Selection Methods. *Comput. Electr. Eng.* **2014**, *40*, 16–28.

(4) Khalid, S.; Khalil, T.; Nasreen, S. A Survey of Feature Selection and Feature Extraction Techniques in Machine Learning. In *2014 Science and Information Conference*; IEEE, 2014; pp 372–378.

(5) Jovic, A.; Brkic, K.; Bogunovic, N. A Review of Feature Selection Methods with Applications. In *2015 38th International Convention on Information and Communication Technology, Electronics and Microelectronics (MIPRO)*; IEEE, 2015; pp 1200–1205.

(6) Jolliffe, I. T. Principal Component Analysis: A Beginner's Guide — I. Introduction and Application. *Weather* **1990**, *45*, 375–382.

(7) James, M. Some Methods for Classification and Analysis of Multivariate Observations. *Proc. fifth Berkeley Symp. Math. Stat. Probab.* **1967**, *1*, 281–297.

(8) Steinhaus, H. Sur La Division Des Corp Materiels En Parties. *Bull. Acad. Pol. Sci. IV* **1956**, *IV*, 801–804.

(9) Kuhn, H. W. The Hungarian Method for the Assignment Problem. *Nav. Res. Logist. Q.* **1955**, *2*, 83–97.

(10) Munkres, J. Algorithms for the Assignment and Transportation Problems. *J. Soc. Ind. Appl. Math.* **1957**, *5*, 32–38.

(11) Faran, M.; Ray, D.; Nag, S.; Raucci, U.; Parrinello, M.; Bisker, G. A Stochastic Landscape Approach for Protein Folding State Classification. *J. Chem. Theory Comput.* **2024**, *20*, 5428–5438.

(12) William M . Rand. Objective Criteria for the Evaluation of Clustering Methods. *J. Am. Stat. Assoc.* **1971**, *66*, 846–850.

(13) Okabe, A.; Boots, B.; Sugihara, K. Nearest Neighbourhood Operations with Generalized Voronoi Diagrams: A Review. *Int. J. Geogr. Inf. Syst.* **1994**, *8*, 43–71.

(14) Liu, H.; Setiono, R. Discretization of Ordinal Attributes and Feature Selection. **1995**, 1–18.

(15) Du, Q.; Fowler, J. E. Low-Complexity Principal Component Analysis for Hyperspectral Image Compression. *Int. J. High Perform. Comput. Appl.* **2008**, *22*, 438–448.
